# Potent, Selective Pyrrolopyrimidine PDE11A4 Inhibitors with Improved Pharmaceutical Properties

**DOI:** 10.1021/acsmedchemlett.5c00756

**Published:** 2026-01-30

**Authors:** Shams ul Mahmood, Rama Krishna Boddu, Jeremy Eberhard, Charles S. Hoffman, John Gordon, Dennis Colussi, Wayne Childers, Elvis Amurrio, Marie Danaher, Michy P. Kelly, David P. Rotella

**Affiliations:** † Department of Chemistry and Biochemistry, Sokol Institute of Pharmaceutical Life Sciences, 8087 Montclair State University, Montclair, New Jersey 07043, United States; ‡ Biology Department, 6019 Boston College, Chestnut Hill, Massachusetts 02467, United States; § Moulder Center for Drug Discovery, 15493 Temple University, Philadelphia, Pennsylvania 19140, United States; ∥ Department of Neurobiology, University of Maryland School of Medicine, Center for Research on Aging, 12264 University of Maryland School of Medicine, Baltimore, Maryland 21201, United States

**Keywords:** PDE11A4, phosphodiesterase inhibitor, pharmaceutical properties, cell-based activity, LLPS activity

## Abstract

Previous work demonstrated target engagement with an orally bioavailable, potent, selective PDE11A4 inhibitor in the mouse hypothalamus. This compound was limited by low aqueous solubility, stimulating the need for alternative leads with improved pharmaceutical properties to carry out efficacy studies. This paper outlines optimization of a pyrrolopyrimidine hit leading to a potent, selective PDE11A4 inhibitor with improved pharmaceutical properties and promising activity in cell-based models of enzyme activity.

## Graphical abstract

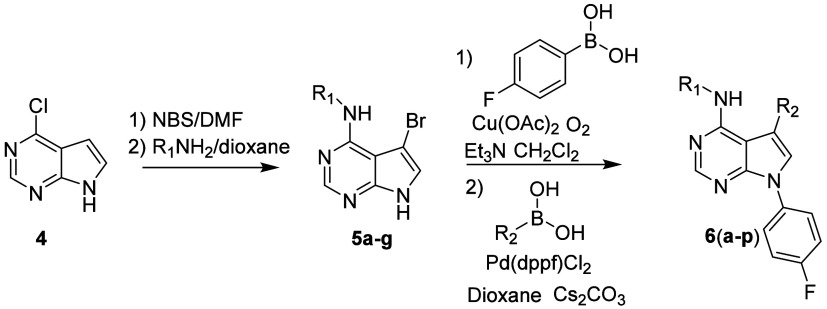

A member of the mammalian phosphodiesterase family, PDE11A4 is expressed and is functionally relevant in rodent and human brain.^1^ PDE11A4 expression increases with age in both the human and rodent hippocampus,^2,3^ a brain region critical for memory storage and retrieval. Preventing or reversing this age-related increase in PDE11A4 protein in mice using genetic approaches rescues age-related cognitive decline of select memories, suggesting inhibition of this dual substrate phosphodiesterase could be a target for treatment of memory disorders.^3–5^ Interestingly, these age-related increases in PDE11A4 protein do not disperse throughout neurons but, rather, cluster into trails of spherical droplets due to liquid–liquid phase separation.^6^ Recent work from our group showed that an orally bioavailable, potent, selective PDE11A4 inhibitor (Figure 1, **1**) engaged the enzyme in mouse hypothalamus to elevate cyclic adenosine monophosphate (cAMP) in a PDE11A4-dependent manner.^10^ Our group also showed that **1** reversed PDE11A4 liquid–liquid phase separation (LLPS) in the hippocampus of old mice and attenuated expression of neuroinflammatory markers.^7^ Continued optimization and investigation of **1** was limited by structure–activity and pharmaceutical property constraints in the pyrazolopyridine chemotype. In the latter category, the low aqueous solubility of **1** did not allow doses of >30 mg/kg to establish a more complete pharmacodynamic effect for cAMP increases and efficacy studies.

**Figure 1:**
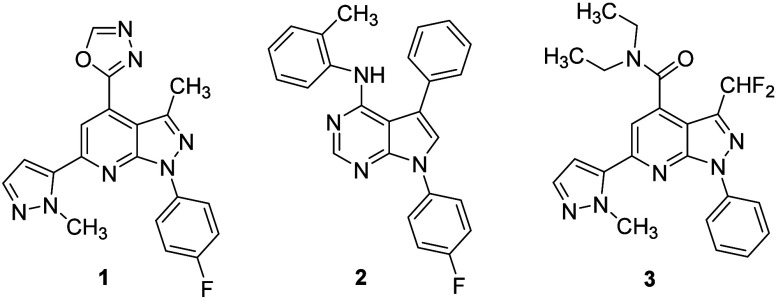
PDE11A4 inhibitors

To address these issues, we chose to explore another PDE11A4 chemotype identified in a previous high-throughput screen^8^ and selected pyrrolopyrimidine **2** (Figure 1). This scaffold offered three different vectors for exploration to address the necessary improvements in pharmaceutical properties and potency. At the outset, we made no assumptions regarding the potential for structure–activity overlaps between **1**, **2**, and **3**,^9^ a metabolically unstable, potent and selective PDE11A4 inhibitor that provided the basis for identification of **1**. This paper reports promising results in our ongoing effort to optimize **2** as a PDE11A4 inhibitor with appropriate properties for *in vivo* evaluation to attempt to validate the role of the enzyme in age-related memory disorders.

Initially we chose to retain the 4-fluorophenyl substituent on the pyrrole nitrogen in **2** to explore variations of the aniline and 3-aryl moieties to improve the potency and aqueous solubility. The synthesis of these derivatives is outlined in Scheme 1. Bromination of chloropyrimidine **4** was followed by displacement using various amines in refluxing dioxane in good to excellent yield. Chan-Lam substitution on the pyrrole nitrogen installed the 4-fluorophenyl group in a modest yield. Suzuki coupling furnished the initial set of analogs (**6a**–**p**) to evaluate structure–activity at these two sites.

**Scheme 1:**
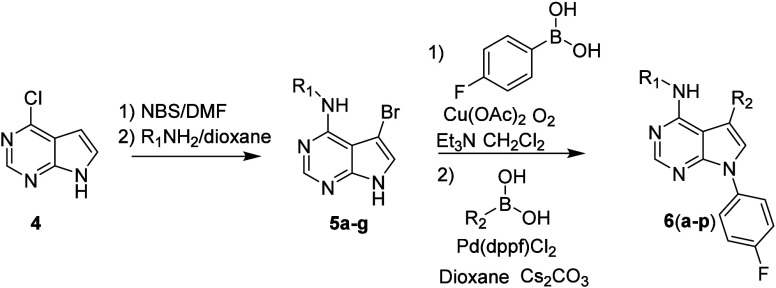
Synthesis of Aniline and 3-Substituted Pyrrole PDE11A4 Inhibitors

Biochemical evaluation was carried out using human PDE11A4 with cAMP as the substrate as described.^8,10^ The results are provided in Table 1 using **1** as a positive control. Replacement of the aniline phenyl with 4-aminopyridine (**6a**), 3-aminopyridine (**6b**), or benzylamine (**6b**) decreased potency relative to the parent aniline (**6d**). Chiral α-methylbenzylamines displayed a substantial stereoselective preference for the R-enantiomer (**6e**) versus the S-enantiomer (**6f**), with the (R)-isomer preferred with potency comparable to that of **6d**. Attention was focused next on the pyrrole 3-phenyl substituent where heterocycles were prioritized to improve lipophilicity and activity. The 3-pyridyl (**6g**), 4-pyridyl (**6h**), and 5-pyrimidinyl (**6i**) derivatives were less potent than the phenyl parent **6d**. More promising results were observed in a small set of five-membered pyrazole heterocycles, where *N*-methyl-4-pyrazolyl **6j** was preferred compared to either the 5-(**6k**) or 3-substituted (**6l**) regioisomers, leading to a PDE11A4 inhibitor with a measurable increase in potency (100 nM) relative to **6d**. Further exploration of nitrogen substitution in the 4-pyrazole showed that alkylation of the nitrogen was preferred (**6m**), as were nonpolar substitutions (**6o**, **6p**), compared to a more polar analog **6n**. This data indicated the aniline substituent in **2** prefers a limited range of nonpolar substitutions and the 3-substituent on the pyrrole ring offers the ability to explore more polar heterocycles.

**Table 1:**
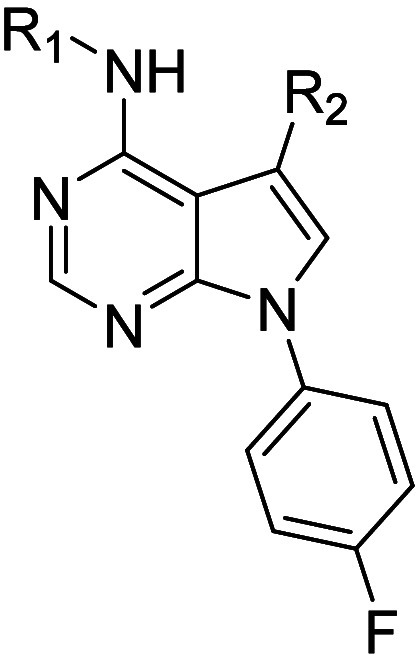

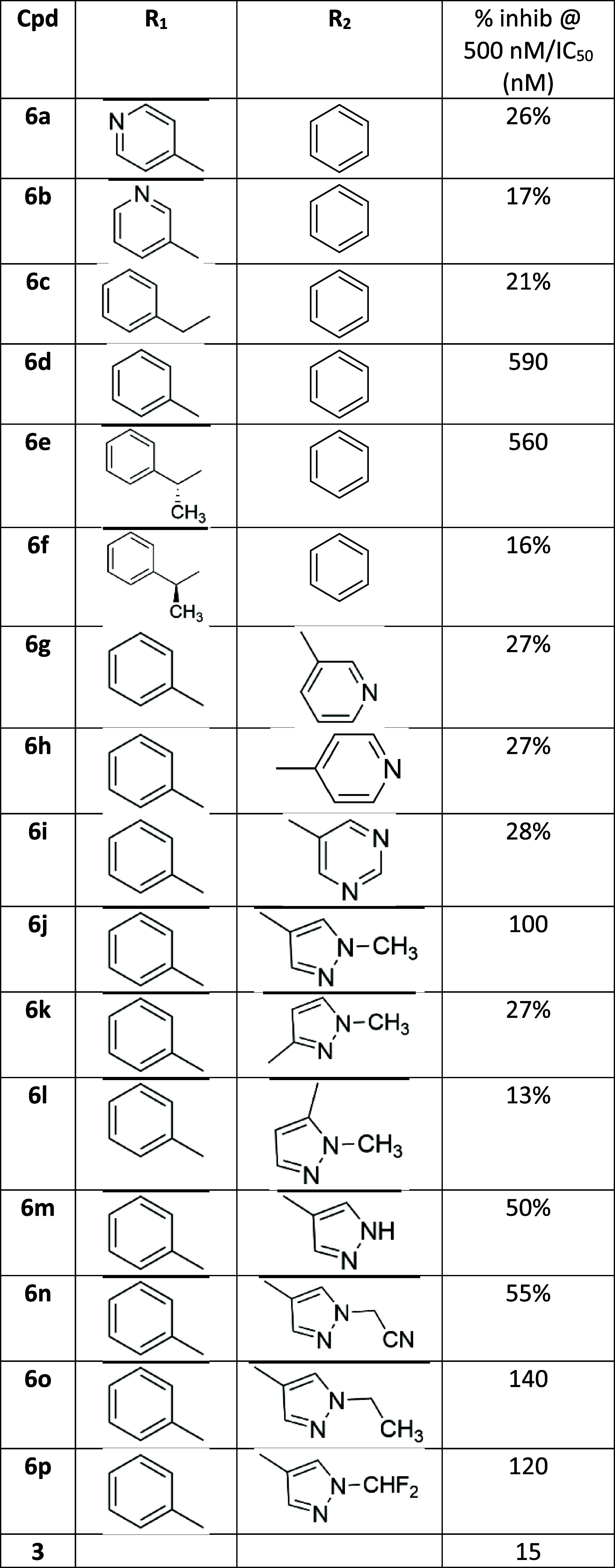
Aniline and 3-Phenyl Structure Activity Results^a^

a IC_50_ values average of 3 independent determinations; % inhibition average of 2 independent determinations.

With this structure–activity information in hand, attention turned to functionalization of the pyrrole nitrogen, initially retaining the aniline and phenyl moieties in **2**. Using Chan-Lam chemistry, a variety of heteroaromatic groups were added in a moderate yield (Scheme 2). We chose to explore sp^3^-based substituents to increase the structural diversity and offer the possibility for subsequent functionalization. Using Mitsunobu chemistry Boc-protected saturated azacycles were appended then deprotected with HCl in dioxane. The basic nitrogen in **13** (derived from **12a**) was functionalized as indicated in Scheme 2 to furnish amide, sulfonamide, and alkylated analogs.

**Scheme 2:**
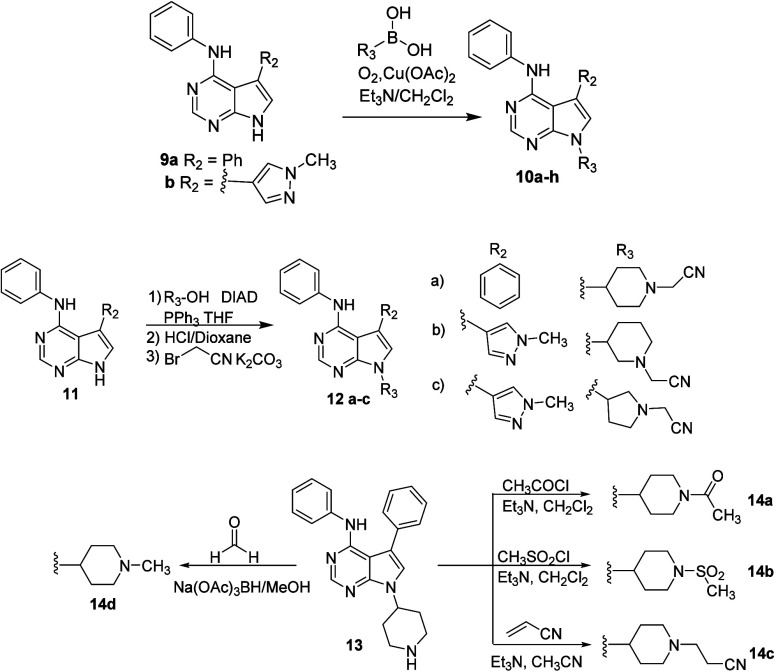
Synthesis of Pyrrole Nitrogen-Substituted PDE11A4 Inhibitors

The data in Table 2 show that heterocycles on the pyrrole ring can increase the potency. For example, 2-, 3-, and 4-pyridyl moieties (**10a**–**c**, respectively) all enhance potency compared to phenyl, with the 3- and 4-isomers being comparable to each other and slightly better than the 2-isomer. A 5-pyrimidinyl derivative **10d** was marginally less active compared to the other pyridyl analogs. Similar results were obtained with *N*-methyl-3- and 4-pyrazoles (**10e**–**f**), with the 4-isomer preferred. Further exploration of nitrogen substituents in the 4-pyrazole isomer (difluoromethyl) **10g** and cyanomethyl (**10h**) had little or no effect relative to the *N*-methyl variation.

**Table 2:**
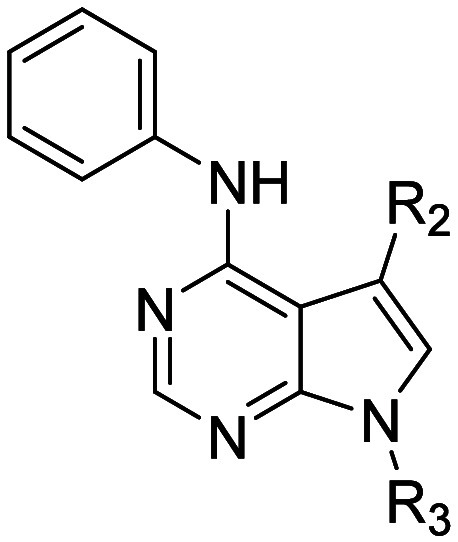

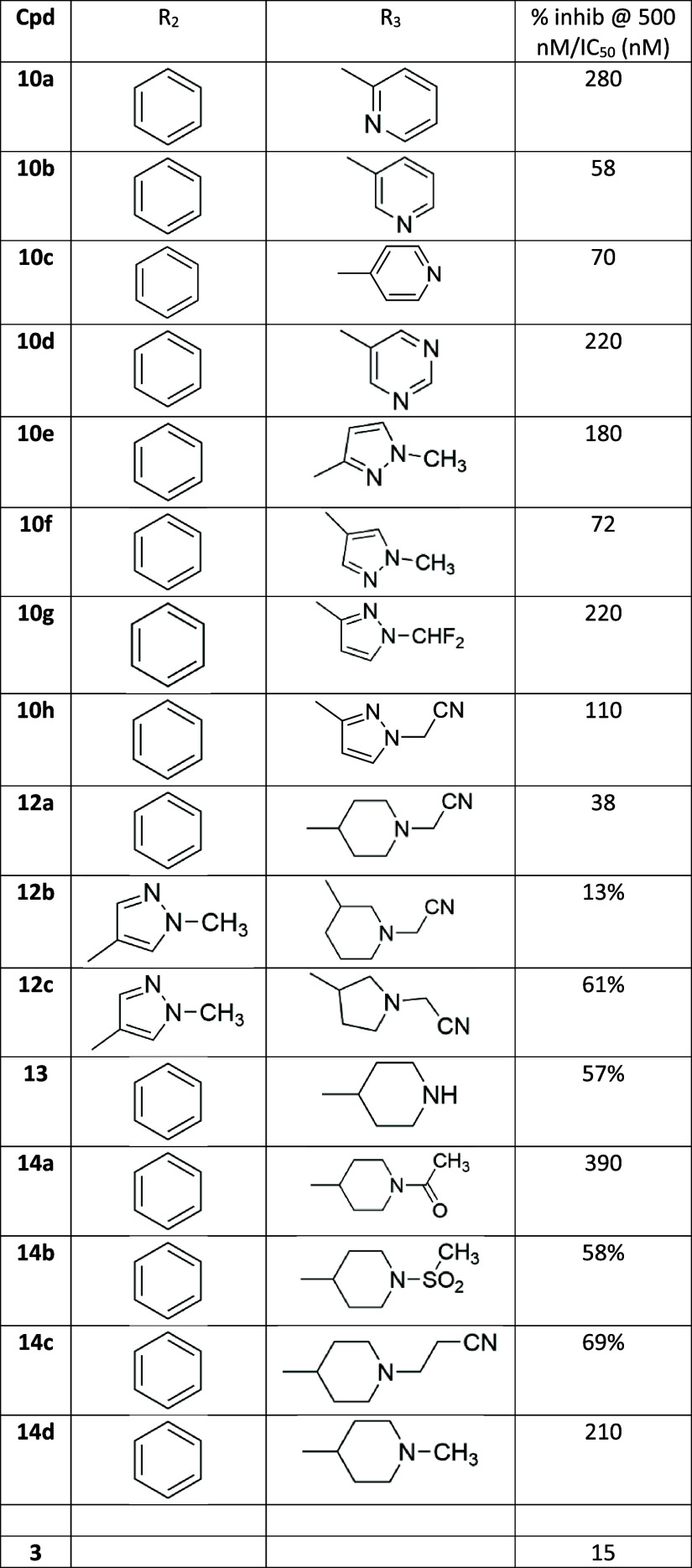
Pyrrole Ring Structure-Activity^a^

a IC_50_ values average of 3 independent determinations; % inhibition average of 2 independent determinations.

Among the saturated azacycles (**12–14**), unsubstituted 4-piperidine **13** was similar to 4-fluorophenyl derivative **6d**. It was slightly less potent compared to the 3- and 4-pyridyl isomers **10b** and **10c**. Substitutions on the piperidine nitrogen had a significant effect on PDE11A4 inhibition. *N*-methylation (**14d**) improved potency somewhat, and acetylation (**14a**) furnished moderate potency; sulfonamide **14b** was weaker, and cyanomethylation provided a significant boost in potency (**12a**, IC_50_ 38 nM), within 2-fold of **1**. Homologation of **12a** to **14c** resulted in a substantial decrease in PDE11A4 inhibition. With a preferred cyanomethyl nitrogen substituent identified, racemic 3-piperidinyl (**12b**) and 3-pyrrolidinyl (**12c**) were found to be substantially less active.

These results led us to explore 4-piperidine derivatives in combination with substitutions on the aniline. Synthesis of these analogs proceeded using a methodology analogous to that outlined previously (Scheme 3) to furnish the aniline analogs **15a**-**h**.

**Scheme 3:**
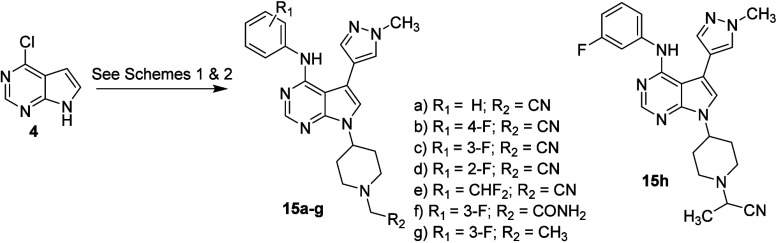
Synthesis of Substituted Aniline-4-piperidinyl Analogs

As shown in Table 3, combining the preferred 4-substituted piperidine with *N*-methyl-4-pyrazolyl and an unsubstituted aniline (**15a**) led to a small decrease in PDE11A4 potency (IC_50_ = 72 nM) compared to phenyl-substituted **12a**. However, potency was restored in the 3-fluoro analog **15c** (IC_50_ 33 nM) and reduced in the 4-F, 2-F, and 3-CHF_2_ derivatives **15b**, **d**, and **e**, respectively. Substitution of a primary amide (**15f**) for the nitrile resulted in a modest drop in potency (IC_50_ 85 nM), while addition of an α-methyl moiety in nitrile analog **15h** substantially reduced activity. Extension of the *N*-methyl moiety in **14d** to ethyl (**15g**) also led to a decreased PDE11A4 potency. This data suggests a binding pocket for the pyrrole nitrogen substituent with limited steric and polar interactions.

**Table 3:**
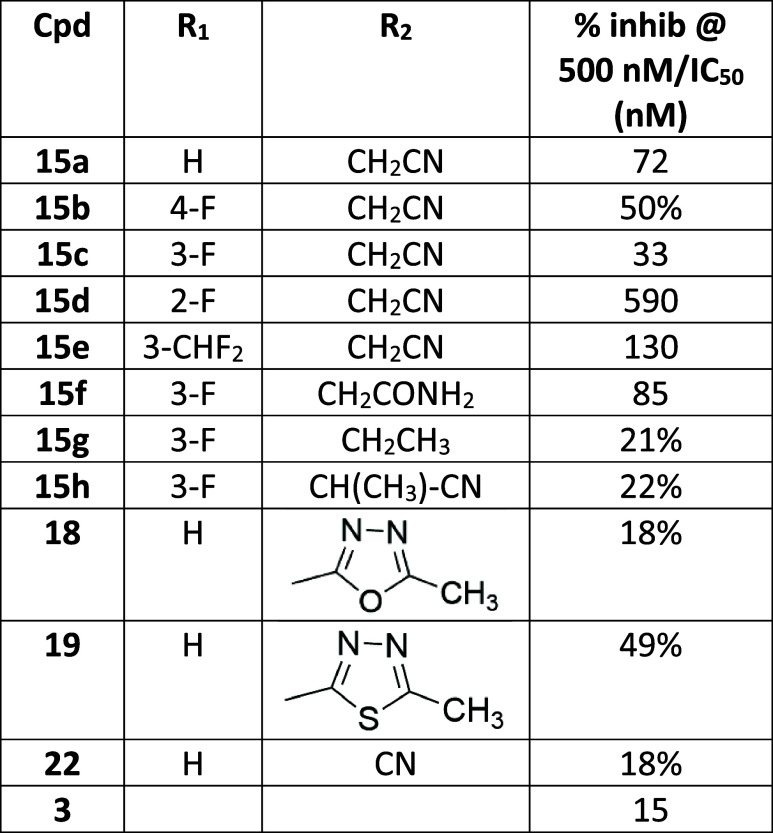
PDE11A4 Potency Compared to Phenyl-Substituted **12a** ^a^

a IC_50_ values average of 3 independent determinations; % inhibition average of 2 independent determinations.

These encouraging results provided an opportunity to explore structure–activity in the diazole heterocycle to further improve potency. Beginning with chloropyrrolopyrimidine ester **16**, aniline addition was followed by Mitsunobu condensation to introduce a protected 4-piperidine, and hydrazide formation gave **17a** and **b** (Scheme 4). Acid-mediated cyclization, deprotection piperidine of the piperidine nitrogen, and alkylation with bromoacetamide provided methyl oxadiazole **18**. The corresponding thiadiazole **19** was prepared as shown in Scheme 4, and a nitrile analog **22** was prepared using analogous chemistry. Unfortunately, none of these modifications improved the potency (Table 3).

**Scheme 4:**
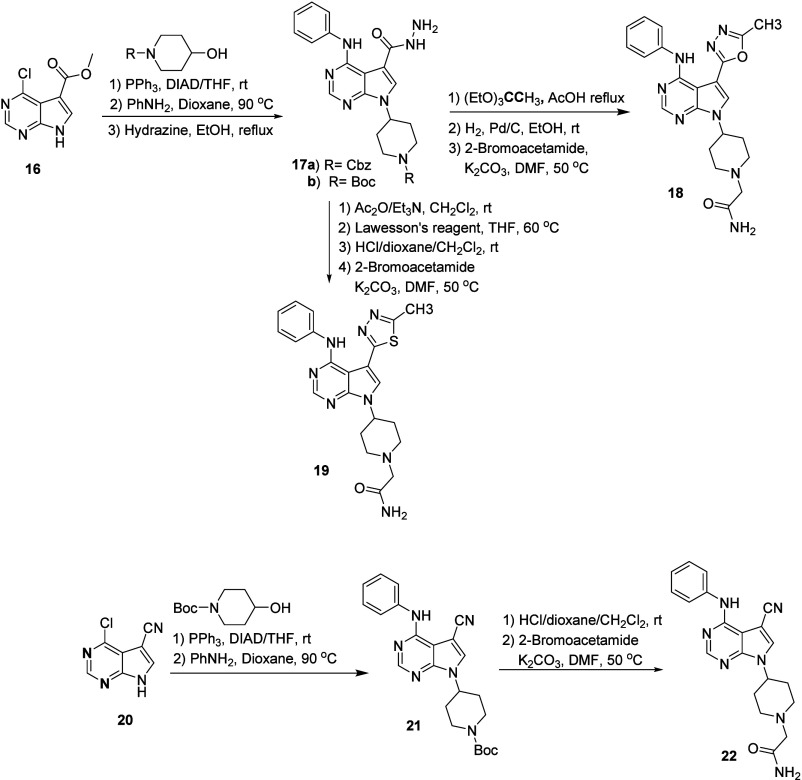
Synthesis of Diazole and Nitrile-Substituted Analogs

With this biochemical potency information in hand, we explored a subset of the most active compounds for PDE selectivity, *in vitro* pharmaceutical properties, and cell-based activity. Selectivity against a panel of key pharmacologically relevant and/or structurally related phosphodiesterases, PDEs 3, 4, 5, 6 and 10, showed that all of these candidates display excellent selectivity for PDE11A4, a trait shared by the original hit **2** (Table 4). The heterocyclic analogs **10b** and **10f** have single-digit micromolar (5–8 μM) aqueous solubility at pH 7.4, a measurable improvement compared to **1** (0.9 μM)^10^ and comparable to **2**. All of these compounds have reduced microsomal stability compared to **2**, with half-lives between 11 and 25 min. We were pleased to note the substantial increase in aqueous solubility displayed by piperidine **15c** (115 μM), and among this group of PDE11A4 inhibitors, **15c** was also the most stable in mouse liver microsomes. Metabolite identification studies using **15c** showed that the cyanomethyl moiety underwent oxidative degradation and was the primary site for CYP-mediated metabolism. Notably, amide **15f** displayed both good aqueous solubility and high microsomal stability.

**Table 4:** PDE Selectivity and Pharmaceutical Property Screen of PDE11A4 Inhibitors^a^

**Cpd**	**PDE3A (cAMP)**	**PDE4D3**	**PDE5A**	**PDE6C**	**PDE10 (cAMP)**	**Aq**. **Sol**. **(μM) pH 7**.**4**	**Mouse liver microsome t**1/2**(min)**
**2** ^8,9^	0	0	0	0	0	3	45
**10b**	8	45	25	12	15	4.8	19
**10f**	5	12	12	10	0	7.7	16
**12a**	0	28	22	15	0	6.0	22
**15a**	0	28	22	12	5	181	14
**15c**	0	5	5	10	0	115	25
**15f**	0	0	10	5	0	180	>60

a PDE selectivity % inhibition @ 500 nM; avg of 2 independent determinations.

The promising activity, preliminary PDE selectivity, and excellent aqueous solubility of piperidine **15c** made it a suitable candidate for cell-based evaluation. Mouse HT22 hippocampal neuronal cells that do not endogenously express mouse PDE11A4 (mPDE11A4) were transfected to either mimic the age-related overexpression of mPDE11A4 that occurs in the brain or express green fluorescent protein (GFP) as a negative control. The difference between these two vehicle-treated groups represents PDE11A4-mediated catalytic activity. Tadalafil (**23**, Figure 2) was used as a positive control because it is a known PDE11 inhibitor,^9,10^ and **15c** demonstrated concentration-dependent reduction in both cAMP and cGMP activity (Figure 2). Cyanomethylpiperidine **15c** reduced both cAMP-PDE11A4 activity (EC_50_ 10.2 μM) and cGMP-PDE11A4 activity (EC_50_ 10.4 μM) in a concentration-dependent manner similar to **3** ^10^ and **1**.^9^ Importantly, the ability of **15c** to inhibit PDE11A4 activity was not due to an inhibitor-induced loss of PDE11A4 protein expression (Figure 2C). Next, we tested the ability of **15c** to reverse the aging-like LLPS of mPDE11A4 in HT22 neuronal cells and LLPS of human PDE11A4 (hPDE11A4) in human SH-SY5Y neuronal cells. **15c** reversed aging-like LLPS of both mPDE11A4 (Figure 3A) and hPDE11A4 (Figure 3B) in a concentration-dependent manner, again with 10 μM **15c** and 10 μM **23** eliciting equivalent effect sizes. Further, **15c** blocked the exacerbation of aging-like PDE11A4 LLPS caused by the global kinase inhibitor staurosporine in both HT22 (Figure 3C) and SH-SY5Y (Figure 3D) neuronal cells. Thus, **15c** exhibits the ability to reverse both basal aging-like PDE11A4 LLPS and the disease-like exacerbation thereof.

**Figure 2:**
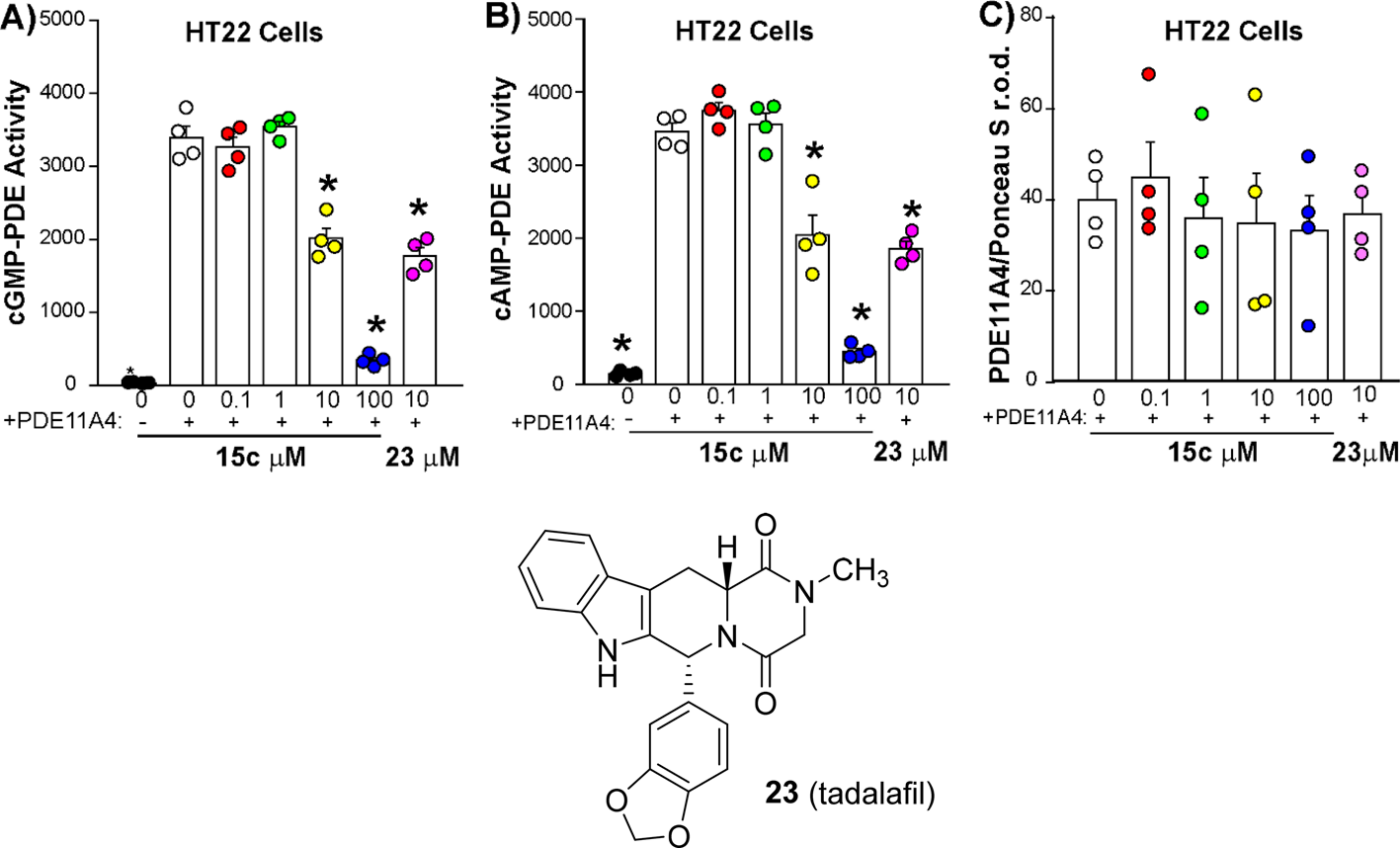
**15c inhibits PDE11A4 in a mammalian cell model of age-related overexpression**. In mouse HT22 cells transfected with PDE11A4 (+), **15c** and the positive control **23** both reduced A) cAMP-PDE11A4 activity F­(6,21) = 119.19, *P* < 0.0001 and B) cGMP-PDE11A4 activity (failed equal variance; H(6) = 24.72, *p* < 0.0001; *n* = 4 biological replicates/group) without changing C) PDE11A4 protein expression. *Post hoc*: *vs PDE11A4 + 0 μM −167, *p* < 0.002–0.0002.

**Figure 3:**
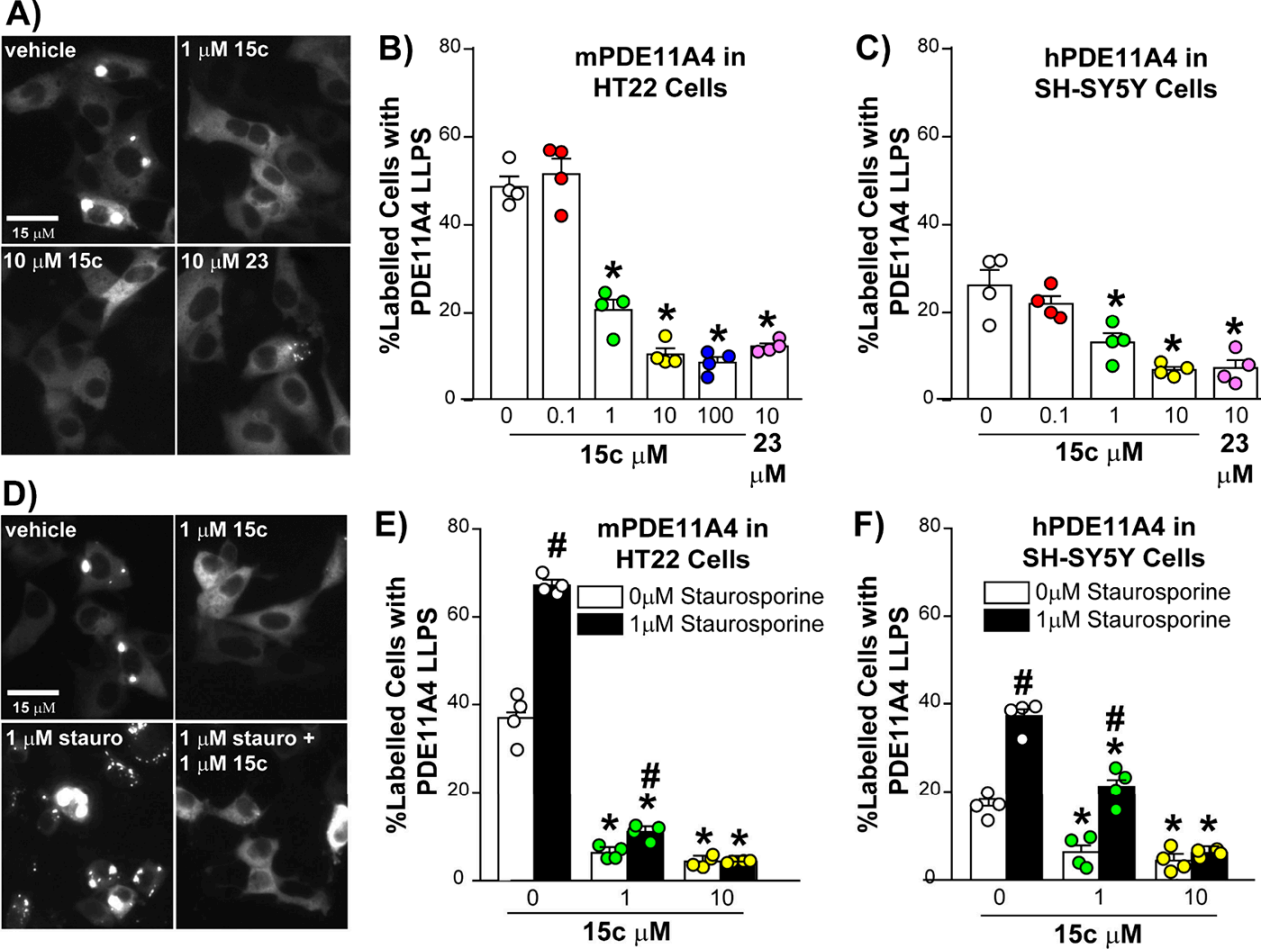
**15c reverses aging- and disease-like LLPS of both human and mouse PDE11A4 in neuronal cells**. A) Widefield microscopy images of mouse HT22 neuronal cells transfected with GFP-tagged mouse PDE11A4 (mPDE11A4) and treated with vehicle, **15c**, or **23**. Quantification shows **15c** reversed aging-like LLPS of B) mPDE11A4 in HT22 neuronal cells (F­(5,18) = 85.18, *P* < 0.0001) and C) hPDE11A4 in human SH-SY5Y neuronal cells (F­(4,15) = 16.14, *P* < 0.0001). D) Images of mPDE11A4-transfected HT22 cells treated with vehicle, **15c**, the broad kinase inhibitor staurosporine that exacerbates PDE11A4 LLPS,^7^ or both **15c** plus staurosporine. Quantification reveals **15c** blocks exacerbated LLPS of both E) mPDE11A4 (F­(2,18) = 79.47, *P* < 0.0001) and F) hPDE11A4 (F­(2,18) = 19.88, *P* < 0.0001). *Post hoc*: *vs PDE11A4+ 0 μM −167, *p* < 0.002–0.0002; #vs 0 μM staurosporine, *p* = 0.0151–0.0002.

This research achieved two of the goals needed to address the weaknesses of **1**. In a distinct pyrrolopyrimidine chemotype **2**, a new PDE11A4 inhibitor **15c** was identified with potency within 2-fold of **1**, while maintaining an excellent preliminary phosphodiesterase selectivity profile. Incorporation of an sp^3^-based piperidine fragment contributed to a marked improvement in aqueous solubility and enabled functionalization that provided an improved potency. While a solution to the metabolic stability of **15c** was identified (**15f**), this reduced the potency. Critically, **15c** demonstrated not only the ability to inhibit PDE11A4 catalytic activity in an *in vitro* model of age-related PDE11A4 overexpression, it also potently reversed PDE11A4 LLPS. Interestingly, **15c** was significantly more potent in the LLPS assay than the cell-based assay. One potential explanation is that inhibitors perturb the interaction network that nucleates PDE11A4 condensates such that modest target engagement is sufficient to cross the LLPS phase boundary, whereas higher occupancy is required to measurably suppress enzymatic activity. Consistent with this model, direct manipulation of PDE11A4 homodimerization bidirectionally regulates LLPS without altering catalytic activity; stabilizing homodimerization increases LLPS and attenuates inhibitor efficacy, and LLPS modulation is unaffected by manipulation of cAMP or cGMP signaling.^6,7^ These properties make **15c** a useful tool compound to study the effects of PDE11A4 activity from both an enzymatic and biophysical perspective. These findings are also consistent with our previous study in which oral dosing of **1** reversed PDE11A4 LLPS in multiple regions of the aged mouse brain at a dose that did not significantly inhibit the enzyme in that brain region.^7^ Together, these results highlight the potential to develop PDE11A inhibitors with scaffold-dependent functional selectivity, enabling preferential modulation of the condensate assembly and spatial signaling independent of maximal catalytic inhibition. Ongoing work is continuing to explore solutions that may provide additional useful tool compounds to test the hypothesis that selective inhibition of PDE11A4 is a target for age-related memory declines. These results will be reported in due course.

*Safety statement*. No unexpected or unusually high safety hazards were encountered

## Supplementary Material





## Notes

Experimental details for synthetic chemistry, pharmaceutical property, cell-based assays and LLPS experiments (PDF)
NMR spectra and HPLC chromatograms for all final compounds (PDF)

## ⊥

SAI Life Sciences, Hyderbad India

## Abbreviations

PDE11A4: phosphodiesterase type 11, isoform A4
LLPS: liquid–liquid phase separation
cAMP: cyclic adenosine monophosphate
DIAD: di-isopropyl azo dicarboxylate
cGMP: cyclic guanosine monophosphare
GFP: green fluorescent protein
