# Correction to “Potent, Selective Pyrrolopyrimidine PDE11A4 Inhibitors with Improved Pharmaceutical Properties”

**DOI:** 10.1021/acsmedchemlett.6c00150

**Published:** 2026-03-26

**Authors:** Shams ul Mahmood, Rama Krishna Boddu, Jeremy Eberhard, Charles S. Hoffman, John Gordon, Dennis Colussi, Wayne Childers, Elvis Amurrio, Marie Danaher, Michy P. Kelly, David P. Rotella

The structure of compound **1** shown in Figure 1 of the original manuscript was inadvertently incorrect. A corrected version of Figure 1 is shown below. This correction does not change any of the data, conclusions or anything else in the manuscript.

**Figure 1:**
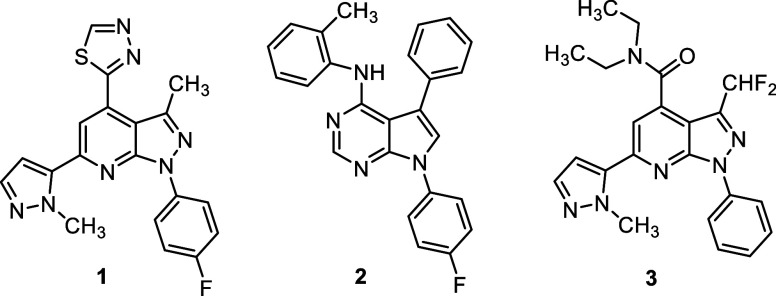
PDE11A4 inhibitors.

