# Supplementary material for: Potent, Selective Pyrrolopyrimidine PDE11A4 Inhibitors with Improved Pharmaceutical Properties
Source: ACS Med Chem Lett. 2026 Jan 30;17(2):547–53. doi: 10.1021/acsmedchemlett.5c00756 (PMC12907953; doi:10.1021/acsmedchemlett.5c00756)
Supplement: Supplementary file 1 [file ml5c00756_si_001.pdf]

Supporting Information

Potent, Selective Pyrrolopyrimidine PDE11A4 Inhibitors with Improved Pharmaceutical Properties

Shams ul Mahmood[1,2], Rama Krishna Boddu[1,2], Jeremy Eberhard[3], Charles S. Hoffman[3], John Gordon[4], Dennis Colussi[4], Wayne Childers[4], Elvis Amurrio[5], Marie Danaher[5], Michy P. Kelly[5,6],David P. Rotella[*] [1,2]

[1] Department of Chemistry and Biochemistry, [2] Sokol Institute of Pharmaceutical Life Sciences, Montclair State University, [3] Biology Department, Boston College, [4] Moulder Center for Drug Discovery, Temple University, [5] Department of Neurobiology, University of Maryland School of Medicine, [6] Center for Research on Aging, University of Maryland School of Medicine

Corresponding author email: rotellad@montclair.edu

Author email:

Shams ul Mahmood: mahmoods@montclair.edu

Rama Krishna Boddu: bodduramakrishna87@gmail.com

Jeremy Eberhard: eberharj@bc.edu

Charles S. Hoffman: charles.hoffman@bc.edu

John Gordon: john.gordon@temple.edu

Dennis Colussi: dennis.colussi@temple.edu

Wayne Childers: wayne.childers@temple.edu

Elvis Amurrio: eamurrio@som.umaryland.edu

Marie Danaher: mdanaher@som.umaryland.edu

Michy P. Kelly: michy.kelly@som.umaryland.edu

Experimental Section

All reagents and solvents were used as received from commercial suppliers. All reactions were carried out under a nitrogen atmosphere unless otherwise stated. Compounds were analyzed by analytical TLC (silica gel G UV254), HPLC (CEM LC, Restek C18 5 μM column, 150 mm x 4.6 mm, eluting with an isocratic 80% acetonitrile/20% water plus 0.1%

formic acid) over 6 minutes with UV detection at 254 nm. Flash chromatography was carried out using a Teledyne Isco Rf200+ automated system. Proton NMR were obtained on a Bruker 400 MHz instrument using $CDCl_3$ and/or CD_3OD. All compounds were at least 90% pure by HPLC and/or NMR analysis except for **10b** (85%) and **22** (86%).

6a-p

To a solution of 4- chloro-pyrrolopyrimidine (5 g, 32.5 mmol) in 50 ml DMF, NBS (7 g, 39.0 mmol) was added at room temperature and stirred for overnight. The reaction mixture was poured into ice cold water and the precipitates that formed were collected by filtration, washed with water and dried to provide product (90% yield) that was used without further purification.

Synthesis of aryl/heteroaryl intermediates 5a-g.

3-Bromo-4-chloro-pyrrolopyrimidine described above (1 eq.) was suspended in 1,4-dioxane, and the respective anilines (3 eq.) were added. The reaction was heated at 90°C overnight. The reaction mixture was poured into ice cold water and the precipitate that formed was collected by filtration washed with water and dried. Yield 85-90%

General procedure for synthesis of 6a-p.

a) Chan-Lam coupling

The respective phenyl substituted pyrrolopyrimidines **5a-g** (1 eq.) and appropriate boronic acid or ester (2 eq.) were dissolved in methylene chloride in a round bottom flask. To this mixture triethylamine (3 eq.) was added dropwise and oxygen was bubbled into the solution for five minutes. Anhydrous copper acetate (1.5 eq.) added and stirred at room temperature under an oxygen balloon overnight. The reaction mixture diluted with methylene chloride and washed with two portions each water, dilute aqueous ammonia solution, brine and dried with Na_2SO_4 and evaporated. The crude product was purified by automated flash chromatography using an appropriate ethyl acetate:hexane system. Yields 40-50%. These intermediates underwent Suzuki coupling using the following general procedure.

b) Suzuki coupling

3-Bromo pyrrolopyrimidines (1 eq) and Boronic acids/esters (2.5 eq.) were suspended in dioxane/water (4:1) and Cs_2CO_3 (5 eq.) was added to the reaction mixture under N_2 environment. After flushing under N_2 for five minutes, Pd(dppf)Cl_2 (0.2 eq.) was added followed by a N_2 flush for 5 minutes. Then reaction mixture was stirred at 80°C overnight and was diluted with ethyl acetate then washed twice with water and brine. The organic extract was dried with Na_2SO_4 and concentrated. The crude product was purified by automated flash chromatography using an appropriate ethyl acetate/hexane system. Yield 50-60%

6a: ^{1}H NMR (400 MHz, DMSO-*d6*) *δ* 12.2 (s, 1H), 8.04-8.01 (m, 4H), 7.87-7.83 (m, 4H), 7.47-7.40 (m, 6H),7.31-7.30 (m, 2H).

6b: ^{1}H NMR (400 MHz, $CDCl_3$) *δ* 8.40 (s, 1H), 7.97 (s, 1H), 7.74-7.70 (m, 2H), 7.68-7.64 (m, 1H), 7.58-7.56 (m, 4H), 7.51-7.47 (m, 2H), 7.31-7.27 (m, 5H).

6c: ^{1}H NMR (400 MHz, $CDCl_3$) *δ* 8.53 (s, 1H), 7.76-7.72 (m, 2H), 7.56-7.54 (m, 2H), 7.48-7.22 (m, 12H), 5.53 (br, 1H), 4.87-4.85 (d, *J*= 5.6 Hz, 2H).

6d: ^{1}H NMR (400 MHz, $CDCl_3$) *δ* 8.61 (s, 1H), 7.77-7.75 (m, 2H), 7.68-7.54 (m, 7H), 7.38-7.27 (m, 3H), 7.18 (br, 1H), 7.13-7.11 (m, 1H).

6e: ^{1}H NMR (400 MHz, $CDCl_3$) *δ* 8.49 (s, 1H), 7.74-7.71 (m, 2H), 7.59-7.52 (m, 2H), 7.50-7.47 (m, 3H), 7.37-7.30 (m, 3H), 7.28-7.21 (m, 1H), 5.61-5.57 (m, 1H), 5.51-5.49 (m, 1H), 1.55-1.53 (d, *J*= 7.6 Hz, 3H).

6f: ^{1}H NMR (400 MHz, $CDCl_3$) *δ* 8.50 (s, 1H), 7.74-7.70 (m, 2H), 7.59-7.56 (m, 2H), 7.54-7.47 (m, 3H), 7.36-7.30 (m, 7H), 7.28-7.21 (m, 1H), 5.60-5.57 (m, 1H), 5.51-5.49 (m, 1H), 1.54-1.53 (d, *J*= 6.8 Hz, 3H).

6g: ^{1}H NMR (400 MHz, $CDCl_3$) *δ* 9.27 (br, 1H), 8.58 (br, 1H), 8.54 (s, 1H), 8.11 br, 1H), 7.79 (br, 3H), 7.58-7.56 (m, 4H), 7.34-7.30 (m, 2H), 7.26-7.22 (m, 2H), 7.09-7.07 (t, *J*= 7.2 Hz), 1H).

6h: ^{1}H NMR (400 MHz, $CDCl_3$) *δ* 8.81 (br, 2H), 8.61 (s, 1H), 7.76-7.73 (m, 2H), 7.63-7.61 (m, 4H), 7.45-7.41 (m, 3H), 7.39-7.30 (m, 2H), 7.16-7.13 (t, *J*= 7.2 Hz, 1H), 6.97 (br, 1H).

6i: ^{1}H NMR (400 MHz, DMSO-*d6*) *δ* 9.17 (s, 1H), 9.05 (s, 2H), 8.56 (s, 1H), 8.49 (s, 1H), 8.21 (s, 1H), 8.00-7.97 (m, 2H), 7.58-7.56 (d, *J*= 8 Hz, 1H), 7.52-7.48 (t, *J*= 8.4 Hz, 2H), 7.33-7.31 (t, *J*= 7.2 Hz, 2H), 7.08-7.04 (t, *J*= 7.2 Hz, 1H).

6j: ^{1}H NMR (400 MHz, $CDCl_3$) δ 8.57 (s, 1H), 7.76-7.70 (m, 3H), 7.63-7.60 (m, 3H), 7.41-7.37 (t, *J*= 15.6 Hz, 2H), 7.31-7.29 (m, 4H), 7.16-7.14 (t, *J*= 7.2 Hz, 1H), 4.07 (s, 3H).

6k: ^{1}H NMR (400 MHz, DMSO-d_6) δ 12.28 (s, 1H), 8.43 (s, 1H), 8.34 (s, 1H), 8.04-7.90 (m, 5H), 7.49-7.42 (m, 4H), 7.13-7.10 (m, 1H), 6.89-6.89 (d, *J*= 2 Hz, 1H), 4.10 (s, 3H).

6l: ^{1}H NMR (400 MHz, $CDCl_3$) δ 8.62 (s, 1H), 7.76-7.73 (m, 3H), 7.57-7.54 (m, 2H), 7.40-7.30 (m, 7H), 7.15-7.13 (t, *J*= 7.2 Hz, 1H), 6.52 (br, 1H), 3.96 (s, 3H).

6m: ^{1}H NMR (400 MHz, DMSO-d_6) δ 13.26 (s, 1H), 8.46 (s, 1H), 8.17 (s, 1H), 7.97-7.94 (m, 2H), 7.89 (s, 1H), 7.83 (s, 1H), 7.75 (s, 1H), 7.66-7.64 (d, *J*= 7.6 Hz, 2H), 7.48-7.44 (t, *J*= 8.2 Hz, 2H), 7.39-7.35 (t, *J*= 8.0 Hz, 2H), 7.10-7.06 (t, *J*= 7.6 Hz, 1H).

6n: ^{1}H NMR (400 MHz, DMSO-d_6) δ 8.40 (s, 1H), 8.24 (s, 1H), 7.98-7.94 (m, 3H), 7.91 (s, 1H), 7.86 (s, 1H), 7.70-7.69 (d, *J*= 7.6, 2H), 7.49-7.44 (m, 2H), 7.39-7.35 (t, *J*= 7.6 Hz, 2H), 7.10-7.07 (t, *J*= 7.2 Hz, 1H), 5.66 (s, 2H).

6o: ^{1}H NMR (400 MHz, DMSO-d_6) δ 8.41 (s, 1H), 8.18 (s, 1H), 7.96-7.94 (m, 2H), 7.82-7.82 (d, *J*= 2.8 Hz, 2H), 7.78 (s, 1H), 7.69-7.67 (m, 2H), 7.48-7.44 (m, 2H), 7.40-7.36 (m, 2H), 7.10-7.06 (t, *J*= 7.2 Hz, 1H), 4.32-4.27 (q, *J*= 7.2 Hz, 2H), 1.52-1.48 (t, *J*= 7.2 Hz, 3H).

6p: ^{1}H NMR (400 MHz, $CDCl_3$) δ 8.51 (s, 1H), 8.11 (s, 1H), 7.95 (s, 1H), 7.74-7.70 (m, 2H), 7.64-7.52 (m, 2H), 7.40-7.37 (m, 3H), 7.31-7.22 (m, 3H), 7.15-7.10 (m, 2H).

Synthesis of 10a-h

Intermediates **9a** and **9b** were synthesized as described above beginning with 3-bromo-4-chloropyrrolopyrimidine followed by aniline displacement and Suzuki coupling. Chan-Lam coupling was then carried out as described above to furnish the target compounds. Compounds were purified by automated flash chromatography eluting with ethyl acetate/hexane mixtures.

10a: ^{1}H NMR (400 MHz, $CDCl_3$) δ 8.88-8.86 (d, *J*= 8.4 Hz, 1H), 8.67 (s, 1H), 8.57-8.55 (m, 1H), 8.20 (s, 1H), 7.98-7.93 (m, 1H), 7.71-7.68 (m, 2H), 7.63-7.54 (m, 5H), 7.39-7.35 (m, 2H), 7.31-7.26 (m, 1H), 7.13-7.09 (m, 2H).

10b: ^{1}H NMR (400 MHz, $CDCl_3$) δ 8.60 (s, 1H), 8.50 (s, 1H), 7.69-7.54 (m, 8H), 7.39-7.31 (m, 4H), 7.17-7.09 (m, 2H), 3.80 (br, 1H).

10c: ^{1}H NMR (400 MHz, $CDCl_3$) δ 8.66 (s, 1H), 7.67-7.55 (m, 9H), 7.49 (s, 1H), 7.39-7.35 (m, 3H), 7.31 (s, 1H), 7.15-7.08 (m, 1H), 3.78 (br, 1H).

10d: ^{1}H NMR (400 MHz, $CDCl_3$) δ 9.31 (br, 1H), 8.68 (s, 1H), 7.69-7.63 (m, 4H), 7.61-7.58 (m, 4H), 7.40-7.36 (m, 4H), 7.31 (s, 1H), 7.15-7.11 (m, 2H).

10e: ^{1}H NMR (400 MHz, $CDCl_3$) δ 8.68 (br, 1H), 7.65-7.63 (m, 2H), 7.60-7.57 (m, 4H), 7.52-7.48 (m, 2H), 7.37-7.31 (m, 3H), 7.23 (br, 1H), 7.12-7.09 (m, 2H), 3.95 (br, 3H).

10f: ^{1}H NMR (400 MHz, $CDCl_3$) δ 8.65 (s, 1H), 8.21 (s, 1H), 7.86 (s, 1H), 7.67-7.58 (m, 5H), 7.55-7.51 (m, 1H), 7.38-7.34 (m, 2H), 7.31 (s, 2H), 7.27 (s, 1H), 7.12-7.08 (m, 2H), 4.05 (s, 3H), 3.45 (br, 1H).

10g: ^{1}H NMR (400 MHz, $CDCl_3$) δ 8.68 (m, 2H), 8.29 (s, 1H), 7.67-7.54 (m, 7H), 7.46 (s, 1H), 7.39-7.35 (t, *J*= 7.6 Hz, 2H), 7.31-7.31 (d, *J*= 2.8 Hz, 1H), 7.15-7.08 (m, 2H).

10h: ^{1}H NMR (400 MHz, $CDCl_3$) δ 8.61 (s, 1H), 8.47 (s, 1H), 8.00 (s, 1H), 7.66-7.55 (m, 7H), 7.39-7.35 (m, 2H), 7.31 (s, 1H), 7.29-7.09 (m, 3H), 5.21 (s, 2H).

Synthesis of 12a-c, 13, 14a-d

General procedure for Mitsunobu reaction

The appropriate pyrrolopyrimidine (1 eq.) and N-Boc protected azacyclic alcohol (1.5 eq.) and PPh_3 (1.5 eq.) were dissolved in dry THF then cooled to 0°C. A solution of diisopropyl azodicarboxylate in dry THF (1.5 eq.) was added dropwise at 0°C and stirred for five minutes at that temperature. The reaction was then warmed to room temperature and stirred overnight. The reaction was quenched by pouring into ice, followed by extraction with three portions of ethyl acetate. The organic extracts were washed with two portions of water and brine, dried with Na_2SO_4 and evaporated. The mixture was purified by automated flash chromatography eluting with an appropriate ethyl acetate/hexane system. Yield 90-92%

4M HCl/dioxane was added to a methylene chloride solution of the Boc-protected azacycles above and stirred at room temperature for 2 hours. The solvent was removed by rotary evaporation and the residual solid washed with ether, and dried under vacuum pump to get the product quantitatively.

13: ^{1}H NMR (400 MHz, CD_3OD) δ 8.34 (s, 1H), 7.72-7.68 (m, 3H), 7.60-7.49 (m, 7H), 7.45-7.43 (m, 1H), 5.21 (m, 1H), 3.71-3.68 (m, 2H), 3.37-3.35 (m, 3H), 2.60-2.52 (m, 2H), 2.43-2.39 (m 2H).

The deprotected azacycle HCl salts were suspended in acetonitrile at room temperature and K_2CO_3 (5 eq.) was added. To this suspension bromoacetonitrile (2 eq.) was added and the reaction was refluxed for 3 hours. The reaction was poured into ice water and extracted with three portions of methylene chloride. The collected organic extract was washed with two portions of brine, dried using Na_2SO_4 and concentrated. Purification by automated flash chromatography using 100% ethyl acetate or 5% methanol/methylene chloride furnished the target compounds. Yield 45-50%

12a: ^{1}H NMR (400 MHz, $CDCl_3$) δ 8.56 (s, 1H), 7.61-7.56 (m, 6H), 7.52-7.50 (m, 1H), 7.37-7.31 (m, 2H), 7.12-7.07 (m, 3H), 4.88-4.83 (m, 1H), 3.64 (s, 2H), 3.06-3.03 (m, 2H), 2.75-2.68 (m, 2H), 2.90-2.11 (m, 4H).

12b: ^{1}H NMR (400 MHz, $CDCl_3$) δ 8.52 (s, 1H), 7.73 (s, 1H), 7.65-7.62 (m, 2H), 7.59 (s, 1H), 7.40-7.36 (m, 2H), 7.31 (s, 1H), 7.23 (s, 1H), 7.17 (s, 1H), 7.13-7.10 (m, 1H), 5.07-5.06 (m, 1H), 4.08 (s, 3H), 3.67-3.66 (m, *J*= 5.2, 2H), 3.15-3.12 (m, 1H), 2.87-2.84 (m, 1H), 2.75-2.70 (m, 1H), 2.18-2.09 (m, 1H), 1.99-1.89 (m, 4H).

12c: ^{1}H NMR (400 MHz, $CDCl_3$) δ 8.52 (s, 1H), 7.73 (s, 1H), 7.64-7.59 (m, 3H), 7.40-7.31 (m, 2H), 7.26 (s, 1H), 7.14-7.12 (t, *J*= 7.2 Hz, 1H), 5.69-5.63 (m, 1H), 4.07 (s, 3H), 3.81-3.80 (d, *J*= 3.6 Hz, 2H), 3.26-3.21 (m, 1H), 3.15-3.06 (m, 2H), 2.83-2.76 (m, 1H), 2.74-2.67 (m, 1H), 2.21-2.15 (m, 1H).

14a

Acetyl chloride (100 mg, 0.24 mmol) was added dropwise into a suspension of **13** (100 mg, 0.24 mmol) and triethylamine (125 mg, 1.2 mmol) in methylene chloride (5 mL) at 0°C. The reaction was warmed to room temperature and stirred for 4 hours. The reaction was diluted with methylene chloride and washed with two portions of 5% $NaHCO_3$ solution, water and brine then dried with Na_2SO_4 and concentrated. Purification by automated flash chromatography eluting with 100% ethyl acetate furnished the desired compound in 57% yield.

^{1}H NMR (400 MHz, $CDCl_3$) δ 8.56 (s, 1H), 7.60-7.55 (m, 6H), 7.52-7.49 (m, 1H), 7.37-7.31 (m, 2H), 7.10-7.07 (m, 3H), 5.06-5.02 (m, 1H), 4.96-4.91 (m, 1H), 4.07-4.03 (m, 1H), 3.42-3.35 (m, 1H), 2.86-2.79 (m, 1H), 2.30-2.20 (m, 5H), 2.07-1.90 (m, 2H).

14b

Methanesulfonyl chloride (85 mg, 0.74 mmol) was added dropwise into a suspension of **13** (100 mg, 0.24 mmol) and triethylamine (125 mg, 1.2 mmol) in methylene chloride (5 mL) at 0°C. The reaction was warmed to room temperature and stirred for 4 hours. The reaction was diluted with methylene chloride and washed with two portions of 5% $NaHCO_3$ solution, water and brine then dried with Na_2SO_4 and concentrated. Purification by automated flash chromatography eluting with 100% ethyl acetate furnished the desired compound in 55% yield.

^{1}H NMR (400 MHz, $CDCl_3$) δ 8.55 (s, 1H), 7.60-7.58 (m, 5H), 7.52-7.51 (m, 2H), 7.38-7.34 (m, 2H), 7.12-7.10 (m, 3H), 5.01-4.95 (m, 1H), 4.10-4.07 (m, 2H), 3.05-2.99 (m, 2H), 2.92 (s, 3H), 2.32-2.19 (m, 4H).

14c

Acrylonitrile (26 mg, 0.49 mmol) was added dropwise to a suspension of **13** (100mg, 0.24 mmol) and triethylamine (125 mg, 1.2 mmol) in acetonitrile and stirred at room temperature overnight. The reaction mixture was diluted with water and extracted with three portions of methylene chloride. The collected organic extract was washed with brine and dried with Na_2SO_4. The product was obtained following automated flash chromatography eluting with 5% methanol/methylene chloride. Yield 45%

^{1}H NMR (400 MHz, $CDCl_3$) δ 8.56 (s, 1H), 7.61-7.56 (m, 5H), 7.51-7.49 (m, 2H), 7.37-7.31 (m, 2H), 7.16 (s, 1H), 7.10-7.07 (m, 2H), 4.87-4.82 (m, 1H), 3.15-3.12 (m, 2H), 2.84-2.81 (t, *J*= 7.2 Hz, 2H), 2.63-2.59 (t, *J*= 6.8 Hz, 2H), 2.47-2.41 (m, 2H), 2.20-2.13 (m, 4H).

14d

To a mixture of **13** (100 mg, 0.24 mmol) in methanol, paraformaldehyde (1 ml) and two drops acetic acid were added respectively under N_2 at room temperature. After 30 minutes, $Na(OAc)_3BH$ (371 mg, 1.7 mmol) was added and stirred overnight. The mixture

was poured into brine and extracted with three portions of methylene chloride, dried with sodium sulfate and evaporated. The mixture was purified by automated flash chromatography eluting with 5% methanol in methylene chloride. Yield 50%

^{1}H NMR (400 MHz, $CDCl_3$) δ 8.56 (s, 1H), 7.61-7.55 (m, 5H), 7.51-7.49 (m, 1H), 7.37-7.31 (m, 2H), 7.16 (s, 1H), 7.10-7.06 (m, 2H), 5.00-4.97 (m, 1H), 4.89-4.80 (m, 1H), 3.10-3.07 (m, 2H), 2.42 (s, 3H), 2.33-2.32 (m, 3H), 2.19-2.16 (m, 3H).

15a-h

15a-e were synthesized as described above (see procedures for **9** and **12a** using appropriately substituted anilines and N-methyl-4-pyrazole boronates**.**

15a: ^{1}H NMR (400 MHz, $CDCl_3$) δ 8.52 (s, 1H), 7.72 (s, 1H), 7.63-7.62 (m, 2H), 7.57 (s, 1H), 7.40-7.36 (m, 2H), 7.28 (s, 1H), 7.14-7.10 (m, 1H), 7.04 (s, 1H), 4.86-4.80 (m, 1H), 4.07 (s, 3H), 3.64 (s, 2H), 3.06-3.03 (m, 2H), 2.74-2.68 (m, 2H), 2.21-2.15 (m, 4H).

15b: ^{1}H NMR (400 MHz, $CDCl_3$) δ 8.48 (s, 1H), 7.71 (s, 1H), 7.58-7.55 (m, 3H), 7.08-7.02 (m, 4H), 4.83-4.78 (m, 1H), 4.07 (s, 3H), 3.63 (s, 2H), 3.05-3.02 (m, 2H), 2.73-2.67 (m, 2H), 2.20-2.13 (m, 4H).

15c: ^{1}H NMR (400 MHz, $CDCl_3$) δ 8.55 (s, 1H), 7.82-7.80 (m, 3H), 7.31-7.25 (m, 2H), 7.13-7.11 (m, 1H), 7.04 (s, 1H), 6.81-6.76 (m, 1H), 4.83 (br, 1H), 4.09 (s, 3H), 3.64 (s, 2H), 3.06-3.03 (m, 2H), 2.71 (br, 2H), 2.20-2.12 (m, 4H).

15d: ^{1}H NMR (400 MHz, $CDCl_3$) δ 8.80-8.75 (m, 1H), 8.54 (s, 1H), 7.68 (s, 1H), 7.62 (s, 1H), 7.47 (s, 1H), 7.22-7.18 (m, 1H), 7.12-7.06 (m, 2H), 7.03-6.97 (m, 1H), 4.84-4.80 (m, 1H), 4.06 (s, 3H), 3.63 (s, 2H), 3.04-3.01 (m, 2H), 2.73-2.66 (m, 2H), 2.21-2.15 (m, 4H).

15e: ^{1}H NMR (400 MHz, $CDCl_3$) δ 8.51 (s, 1H), 7.92 (s, 1H), 7.71 (s, 1H), 7.66-7.64 (m, 1H), 7.58 (s, 1H), 7.44-7.40 (t, *J*= 7.6 Hz, 1H), 7.27 (s, 1H), 7.23-7.21 (m, 1H), 7.04 (s, 1H), 6.82-6.54 (t, *J*= 56.4 Hz, 1H), 4.84-4.76 (m, 1H), 4.06 (s, 3H), 3.61 (s, 2H), 3.03-3.00 (m, 2H), 2.71-2.65 (m, 2H), 2.20-2.13 (m, 4H).

15f was synthesized as described for **12a** using an appropriately substituted aniline and 2-bromoacetamide. The mixture was purified by automated flash chromatography eluting with 5% methanol/methylene chloride. 50% yield.

^{1}H NMR (400 MHz, $CDCl_3$) δ 8.51 (s, 1H), 7.82-7.78 (m, 1H), 7.70 (s, 1H), 7.57 (s, 1H), 7.31-7.22 (m, 3H), 7.18-7.06 (m, 2H), 6.78-6.73 (m, 1H), 6.37 (br, 1H), 4.82-4.78 (m, 1H), 4.06 (s, 3H), 3.19-3.14 (m, 4H), 2.58 (br, 2H), 2.20-2.17 (m, 4H).

15g was synthesized using a reductive amination procedure identical to that described above for **14d** using acetaldehyde. The product was purified by automated flash chromatography eluting with 5% methanol/methylene chloride, 45% yield.

^{1}H NMR (400 MHz, $CDCl_3$) δ 8.51 (s, 1H), 7.87-7.83 (m, 1H), 7.70 (s, 1H), 7.58 (s, 1H), 7.31-7.24 (m, 2H), 7.20 (s, 1H), 7.11-7.09 (m, 1H), 6.80-6.76 (m, 1H), 5.05 (br, 1H), 4.08 (s, 3H), 3.63 (br, 2H), 3.06-3.05 (d, J= 6.8 Hz, 2H), 2.83 (br, 4H), 2.28 (br, 2H), 1.52-1.48 (t, J= 6.8 Hz, 3H).

15h was synthesized as described above using 3-fluoroaniline, N-methyl-4-pyrazole boronate and 2-bromopropionitrile using methodology identical to that described for **12a**.

^{1}H NMR (400 MHz, $CDCl_3$) δ 8.54 (s, 1H), 7.87-7.77 (m, 1H), 7.70 (s, 1H), 7.56 (s, 1H), 7.35-7.24 (m, 2H), 7.13-7.10 (m, 1H), 7.05 (s, 1H), 6.80-6.75 (m, 1H), 4.85-4.84 (m, 1H), 4.07 (s, 3H), 3.80-3.79 (q, J= 7.2 Hz, 1H), 3.19-3.17 (m, 1H), 3.02-3.00 (m, 1H), 2.80-2.79 (m, 1H), 2.50-2.47 (m, 1H), 2.26-2.15 (m, 4H), 1.58-1.56 (d, J= 7.2 Hz, 3H).

Synthesis of 18 and 19.

4-chloropyrrolo[3,2-d]pyrimidine ester **16** (1 eq.), the appropriate Boc- or Cbz-protected 4-piperidinols (1 eq.), and triphenylphosphine (1.5 eq.) were dissolved in THF and diisopropyl azodicarboxylate (1.5 eq.) in THF was added dropwise to the reaction mixture at 0 °C. After stirring at room temperature for 3h, the mixture was diluted with water and extracted with three portions of ethyl acetate. The combined organic extract was washed with brine, dried over anhydrous sodium sulfate, and concentrated under reduced pressure. Purification by automated flash chromatography using an ethyl acetate/ hexane system to afford the desired products in 55–65% yield.

The products of this reaction were (1 eq.) dissolved in dioxane and aniline (3 eq.) was added. The reaction mixture was heated to 90 °C overnight. The mixture was diluted with water and extracted with three portions of methylene chloride. The combined organic extract was washed with brine, dried over anhydrous sodium sulfate, and concentrated under reduced pressure. Purification by automated flash chromatography using an ethyl acetate/ hexane system afforded products in 90-95% yield.

These esters (1 eq.) were dissolved in ethanol, and hydrazine hydrate (10 eq.), was added. The reaction mixture was stirred at 100 °C for 5 hours. The mixture was cooled to room temperature, and the solvent was reduced to half its volume under reduced

pressure. The reaction mixture was diluted with cold water and the resulting solid was collected by filtration, washed with cold water, and dried to afford **17a** and **17b** in in 80-90% yield.

Compound **17a** (1 eq.) was dissolved in a mixture of acetic acid and triethyl orthoacetate (2:1, v/v), and the reaction mixture was stirred at 100 °C for 3 hours. Upon completion of the reaction (monitored by TLC), it was quenched with a saturated sodium bicarbonate solution and extracted with ethyl acetate (3 times). The combined organic layers were washed with water and brine, dried over anhydrous sodium sulfate, and concentrated under reduced pressure. The crude product was purified by automated flash column chromatography using a hexane/ethyl acetate sytem to afford the desired compound in 85% yield.

The above compound was dissolved in ethanol (10 vol) and 10% palladium on carbon was added. The reaction mixture was stirred under a hydrogen atmosphere (1 atm, balloon) at room temperature for 3 hours. After completion of the reaction, it was filtered through a pad of Celite and washed thoroughly with ethanol. The combined filtrates were concentrated under reduced pressure to afford the deprotected amine, which was used directly in the next step without purification.

The amine obtained above (1 eq.) was dissolved in DMF and potassium carbonate (2 eq.) and 2-bromoacetamide (2 eq.) were added at room temperature. The reaction mixture was stirred at 50 °C for 12 hours. Upon completion of the reaction, the mixture was diluted with water and extracted with three portions of ethyl acetate. The combined organic layers were washed with water (3 times) and brine, dried over anhydrous sodium sulfate, and concentrated under reduced pressure. The crude residue was purified by automated flash column chromatography using a dichloromethane/methanol (10%) to afford the desired product **18** in 25% yield.

^{1}H NMR (400 MHz, $CDCl_3$+CD_3OD) δ 8.38 (s, 1H), 7.97 (s, 1H), 7.85 (d, 2H, J = 11.2 Hz), 7.39 (t, 2H, J= 13.2, 6.4 Hz), 7.12 (t, 1H, J= 14, 6.4 Hz), 4.74-4.70 (m, 1H), 3.14-3.18 (m, 4H), 2.66 (s, 3H), 2.58-2.53 (m, 2H), 2.27-2.21 (m, 4H)

19

Compound **17b** (1 equiv) was dissolved in methylene chloride, triethylamine (1.5 eq.) was added and cooled to 0 ^{o}C, acetic anhydride (1 eq.) was added. The reaction mixture was stirred at room temperature for 1 hour. Upon completion of the reaction, it was quenched with a saturated sodium bicarbonate solution and extracted with ethyl acetate (3 times). The combined organic layers were washed with water and brine, dried over anhydrous sodium sulfate, and concentrated under reduced pressure. The crude product was purified by automated flash column chromatography using a hexane/ethyl acetate (90%) to afford the desired compound in 80% yield

The above compound (1 eq.) was dissolved in THF, and Lawesson's reagent (2 eq.) was added. The reaction mixture was stirred at 60 °C for 3 hours. Upon completion of the reaction, the mixture was diluted with water and extracted with ethyl acetate (3 times). The combined organic layers were washed with brine, dried over anhydrous sodium sulfate, and concentrated under reduced pressure. The crude product was purified by automated flash column chromatography using a hexane/ethyl acetate (50%) to afford the desired compound **19** in 80% yield.

^{1}H NMR (400 MHz $CDCl_3$+CD_3OD) δ 8.52-8.51 (m, 1H), 8.01-7.98 (m, 3H), 7.55-7.51 (m, 2H), 7.29-7.25 (m, 1H), 4.92-4.86 (m, 1H), 3.41-3.31 (m, 4H), 2.98 (s, 3H), 2.79-2.74 (m, 2H), 2.41-2.27 (m, 4H)

22

4-Chloro-3-cyanopyrrolopyrimidine **20** was treated with N-Boc-4-hydroxypiperidine and aniline as described for **17b**. Boc cleavage was carried out as described for the synthesis of **19**, followed by alkylation with 2-bromoacetamide. Work up and purification was carried out as described for the synthesis of **18** and **19** to furnish **22** in 78% yield**.**

22

^{1}H NMR (400 MHz $CDCl_3$+CD_3OD) δ 8.63-8.62 (m, 1H), 8.16 (s, 1H), 7.89-7.86 (m, 2H), 7.62-7.58 (m, 2H), 7.40-7.36 (m, 1H), 4.94-4.83 (m, 1H), 3.37-3.33 (m, 4H), 2.74 (t, 2H, J = 20.8, 11.2 Hz), 2.45-2.30 (m, 4H).

Cell biology methods

Plasmid generation. Plasmids were generated as previously described[2]. Briefly, Genscript (Piscataway, NJ) generated constructs expressing either EmGFP alone containing an A206Y mutation to prevent EmGFP dimerization[3] or the mouse *Pde11a* (NM_001081033) or human *Pde11a4* (NM_016953.4) sequence fused at the N-terminal with EmGFP. Note that mouse Mouse *Pde11a* is ~95% homologous and the same length as human *Pde11a4* and so the protein is referred to herein as PDE11A4 for clarity. These constructs were initially generated on a pUC57 backbone and then subcloned into a pcDNA3.1+ mammalian expression vector (Life Technologies; Waltham, MA).

Cell culture and transfection. As previously described [4, 5], cells were maintained in T-75 flasks in Dulbecco's Modified Eagle Medium (DMEM) with sodium pyruvate (GIBCO, Gaithersburg, MD or Corning, Manassas, VA), 1% Penicillin/Streptomycin (P/S); GE Healthcare Life Sciences; Logan, UT), and 10% fetal bovine serum (FBS; Atlanta

Biologicals), with incubators set to 37°C/5% CO_2. Cells were passaged at ~70% confluency using TrypLE Express (GIBCO; Gaithersburg, MD). The day before transfection, cells in DMEM+FBS+P/S were plated in 60 mm dishes or 24-well flat-bottom plates. The day of transfection, the media was replaced with Optimem (GIBCO) and cells were transfected with 0.375 µg of cDNA using 1 microliter Lipofectamine 2000 (Invitrogen; Carlsbad, CA) per mL of Optimem, as per the manufacturer's protocol. ~19 hours post-transfection, the Optimem/Lipofectamine solution was replaced with DMEM+FBS+P/S. Cells continued growing for five hours in the supplemented media and then were pharmacologically treated with DMSO, **15c**, **23** (Tocris, 6311) or staurosporine (medchem express, Hy15141) for 1 hour. Cells in the 60mm dishes were then harvested for measurement of PDE activity and cells in 24-well plates were fixed in 4% paraformaldehyde (Sigma-Aldrich) in 1x PBS and then stored in 1x PBS for quantification of LLPS. Over the course of experiments, cells were sporadically tested for yeast, fungal, and bacterial infections (Invitrogen; Cat#:C7028), with negative results always obtained.

PDE activity assay. As previously described [6], cells were harvested in PDE assay buffer (20mM Tris-HCL and 10mM $MgCl_2$) and homogenized using a tissue sonicator (output control: 7.5, duty cycle: 70, continuous). Samples were then held at 4 °C until processing. **T**otal protein levels were quantified using the DC Protein Assay Kit (Bio-Rad, Hercules, CA) according to the manufacturer's directions. 3 µg of each sample was then processed for cGMP- and/or cAMP-PDE activity using a radiotracer assay based on [7], with some adjustments [4, 8]. Briefly, samples were incubated with 35000-45000 disintegrations/minute of[^{3}H]-cAMP or[^{3}H]-cGMP for 10 minutes. The reaction was then quenched with 0.1M HCl and neutralized using 0.1M Tris. Snake venom was then added to the sample and incubated for 10 minutes at 37 °C. Samples were then run down DEAE A-25 Sephadex columns previously equilibrated in high salt buffer (20 mM Tris-HCl, 0.1% sodium azide, and 0.5M NaCl) and low salt buffer (20 mM Tris-HCl and 0.1% sodium azide). After washing the columns four times with 0.5 mL of low salt buffer, the eluate was mixed with 4 mL of scintillation cocktail, and then CPMs were read on a Beckman-Coulter liquid scintillation counter. 2 reactions not containing any sample lysate were also taken through the assay to assess background, which was subtracted from the sample CPMs. Data was expressed as CPMs/ug protein.

Western Blotting. As previously reported[7], 10μg of total protein was loaded onto 4-12% NuPAGE gels (Invitrogen, Waltham MA) and electrophoresed for one at 180 volts. GFP-transfected cell samples were included on all PDE11A4 blots as a negative control. Protein was transferred onto a 0.45μm nitrocellulose membrane for two hours at 100 mA. Nitrocellulose membranes were then washed twice in tris-buffered saline with 0.1% tween20 (TBS-T) before staining with Ponceau S to determine total protein loading. Note, Ponceau S was chosen over a housekeeping gene as a loading control based on the best-practice statement of the *Journal of Biological Chemistry*[4]. Images of the stained membranes were collected to later quantify the optical density of the total protein stain (i.e., spanning ~200kDa to 10kDa), and then the membranes were rinsed in TBS-T to remove the stain. PVDF membranes were first fixed in 8% acetic acid and then washed in TBS-T. Blots to be probed with our custom PDE11A4 antibody (chicken polyclonal; Aves, #1-8113a; 1:10,000) were blocked in 5% milk while those to be probed with GFP (rabbit polyclonal; Santa Cruz, #sc8334; 1:2000) were blocked in Superblock Blocking Buffer (ThermoFisher, Cat#37515), each with 0.1% Tween 20. Primary antibodies were incubated overnight at 4°C. The next day, membranes were washed 4 x 10 minutes with TBS-T and then incubated for 1 hour at room temperature with a secondary antibody (anti-chicken: Jackson Immunoresearch, 103-035-155 at 1:40 000; anti-rabbit: Jackson Immunoresearch, 111-035-144 at 1:10 000). Subsequently, membranes were washed 3 x 15 minutes in TBS-T. Finally, the membranes were immersed in SuperSignal West Pico Chemiluminescent Substrate (ThermoScientific, Waltham MA) as per manufacturer's directions. Following this step, membranes were wrapped in clear a plastic sheet protector, and exposed to film. Multiple film exposures were taken to ensure signals were within the linear range, and Ponceau S stain and western blot optical densities were quantified using Image J. To account for membrane-membrane variances in film exposure, antibody saturation, chemiluminescence reaction, etc. between blots, Western blot data were normalized to a control condition (e.g. EmGFP-PDE11A4 + vehicle) on each blot, as previously described [10-12].

Quantification of PDE11A4 LLPS. Images for quantification of PDE11A4 LLPS were collected using a Nikon Eclipse TE2000-E Inverted via a 10x/0.40 CS2 ∞/0.17/OFN25/A objective equipped with Photometrics CoolSNAP cf camera and CoolLED pE-300lite LED

illuminator. Representative images for each well were captured using MetaVue v6.2r6 software and saved as jpeg files. This Nikon microscope is located in the UMSOM Department of Neurobiology Imaging Core. As previously described [3,4,12], all images pertaining to an experiment were quantified by an experimenter blind to treatment using the same computer within the same position in the room, the same lighting conditions, and the same percent zoom. Images were loaded onto a gridded template to facilitate keeping track of count locations within the image, and an experimenter scored each image box by box, with cells along the top and left edges of the entire image not included to follow stereological best practices. Images were quantified in a counterbalanced manner such that 1 picture from each condition was evaluated before moving onto a 2nd image from that condition. The experimenter classified cells as exhibiting either diffuse labeling only or as having punctate spherical droplets (with/without diffuse labeling). Data are expressed as the % of the total number of labeled cells that exhibited these punctate spherical droplets (i.e. LLPS of the enzyme).

Pharmaceutical property evaluation

Aqueous solubility and mouse liver microsomal studies were carried out as described previously.[9,10]

Phosphodiesterase assays

Biochemical evaluation using human PDE11A4 and PDEs 3, 4, 5, 6 and 10 were carried out using appropriate substrates under conditions described previously.[9,10]

References:

1. Pathak, G.; Agostino, M. J.; Bishara, K.; Capell, W. R.; Fisher, J. L.; Hegde, S.; Ibrahim, B. A.; Pilarzyk, K.; Sabin, C.; Tuczkewycz, T.; et al. PDE11A negatively regulates lithium responsivity. *Mol. Psychiatry* **2017**, *22* (12), 1714-1724.
2. Jackson, R. J.; Howell, M. T.; Kaminski, A. The novel mechanism of initiation of picornavirus RNA translation. *Trends Biochem. Sci.* **1990**, *15* (12), 477-483.
3. Rybalkin, S. D.; Hinds, T. R.; Beavo, J. A. Enzyme assays for cGMP hydrolyzing phosphodiesterases. *Meth. Mol. Biol.* **2013**, *1020*, 51-62.
4. Fosang, A. J.; Colbran, R. J. Transparency Is the Key to Quality. *J. Biol.Chem.* **2015**, *290* (50), 29692-29694.

5. Patel, N. S.; Klett, J.; Pilarzyk, K.; Lee, D. I.; Kass, D.; Menniti, F. S.; Kelly, M. P. Identification of new PDE9A isoforms and how their expression and subcellular compartmentalization in the brain change across the life span. *Neurobiol. Aging* **2018**, *65*, 217-234.
6. Porcher, L.; Bruckmeier, S.; Burbano, S. D.; Finnell, J. E.; Gorny, N.; Klett, J.; Wood, S. K.; Kelly, M. P. Aging triggers an upregulation of a multitude of cytokines in the male and especially the female rodent hippocampus but more discrete changes in other brain regions. *J. Neuroinflamm.* **2021**, *18* (1), 219.
7. Pilarzyk, K.; Capell, W. R.; Porcher, L.; Rips-Goodwin, A.; Kelly, M. P. Biologic that disrupts PDE11A4 homodimerization in hippocampus CA1 reverses age-related cognitive decline of social memories in mice. *Neurobiol. Aging* **2023**, *131*, 39-51.
8. Sbornova, I.; van der Sande, E.; Milosavljevic, S.; Amurrio, E.; Burbano, S. D.; Das, P. K.; Do, H. H.; Fisher, J. L.; Kargbo, P.; Patel, J.; et al. The Sleep Quality- and Myopia-Linked PDE11A-Y727C Variant Impacts Neural Physiology by Reducing Catalytic Activity and Altering Subcellular Compartmentalization of the Enzyme. *Cells* **2023**, *12* (24) 2839.
9. Mahmood, S. U.; Lozano Gonzalez, M.; Tummalapalli, S.; Eberhard, J.; Ly, J.; Hoffman, C. S.; Kelly, M. P.; Gordon, J.; Colussi, D.; Childers, W.; et al. First Optimization of Novel, Potent, Selective PDE11A4 Inhibitors for Age-Related Cognitive Decline. *J. Med. Chem.* **2023**, *66* (21), 14597-14608.
10. Mahmood, S. U.; Eberhard, J.; Hoffman, C. S.; Colussi, D.; Gordon, J.; Childers, W.; Amurrio, E.; Patel, J.; Kelly, M. P.; Rotella, D. P. First Demonstration of In Vivo PDE11A4 Target Engagement for Potential Treatment of Age-Related Memory Disorders. *J. Med. Chem.* **2024**, *67* (19), 17774-17784.
11. Amurrio, E.; Patel, J. H.; Danaher, M.; Goodwin, M.; Kargbo, P.; Klimentova, E.; Lin, S.; Kelly, M. P. Age-Related Increases in PDE11A4 Protein Expression Trigger Liquid-Liquid Phase Separation (LLPS) of the Enzyme That Can Be Reversed by PDE11A4 Small Molecule Inhibitors. *Cells* **2025**, *14* (12) DOI: 10.3390/cells14120897.
12. Amurrio, E.; Patel, J.; Danaher, M.; Elzaree, L.; Fisher, J. L.; Greene, H.; Kargbo, P.; Kim, P.; Klimentova, E.; Lin, S.; et al. Molecular mechanisms regulating PDE11A4 age-related liquid-liquid phase separation (LLPS) and its reversal by selective, potent and orally-available PDE11A4 small molecule inhibitors both in vitro and in vivo. *bioRxiv* **2025**, 2025.2005.2020.654583.
