# Supplementary material for: Potent, Selective Pyrrolopyrimidine PDE11A4 Inhibitors with Improved Pharmaceutical Properties
Source: ACS Med Chem Lett. 2026 Jan 30;17(2):547–53. doi: 10.1021/acsmedchemlett.5c00756 (PMC12907953; doi:10.1021/acsmedchemlett.5c00756)
Supplement: Supplementary file 2 [file ml5c00756_si_002.pdf]

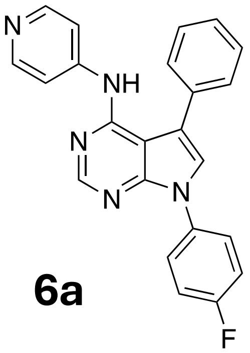

6a

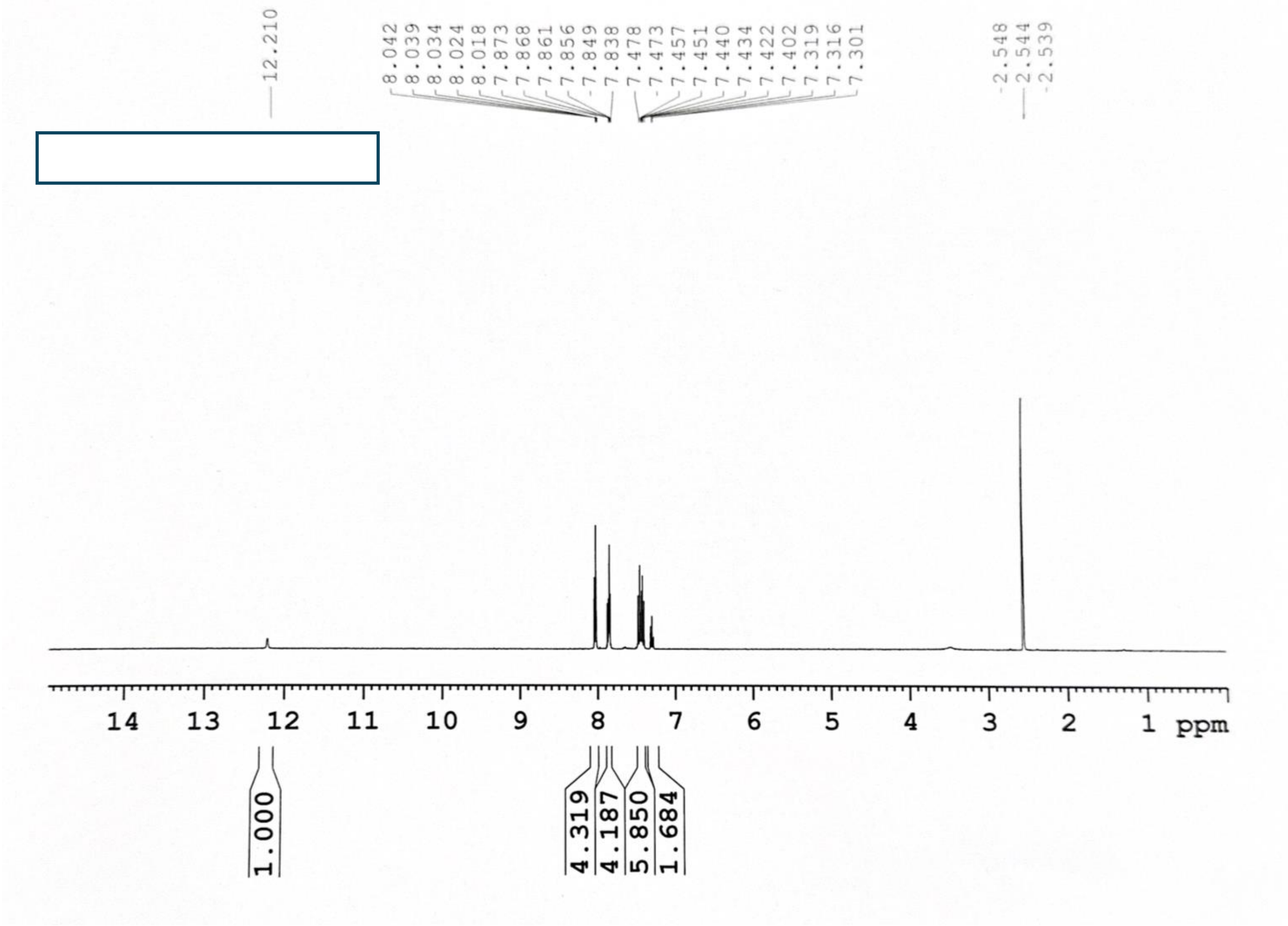

12.210
8.042
8.039
8.034
8.024
8.018
7.873
7.868
7.861
7.856
7.849
7.838
7.478
7.473
7.457
7.451
7.440
7.434
7.422
7.402
7.319
7.316
7.301
2.548
2.544
2.539
14 13 12 11 10 9 8 7 6 5 4 3 2 1 ppm
1.000
4.319
4.187
5.850
1.684

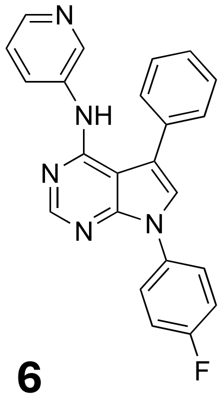
N
NH
N
N
N
F

6
b

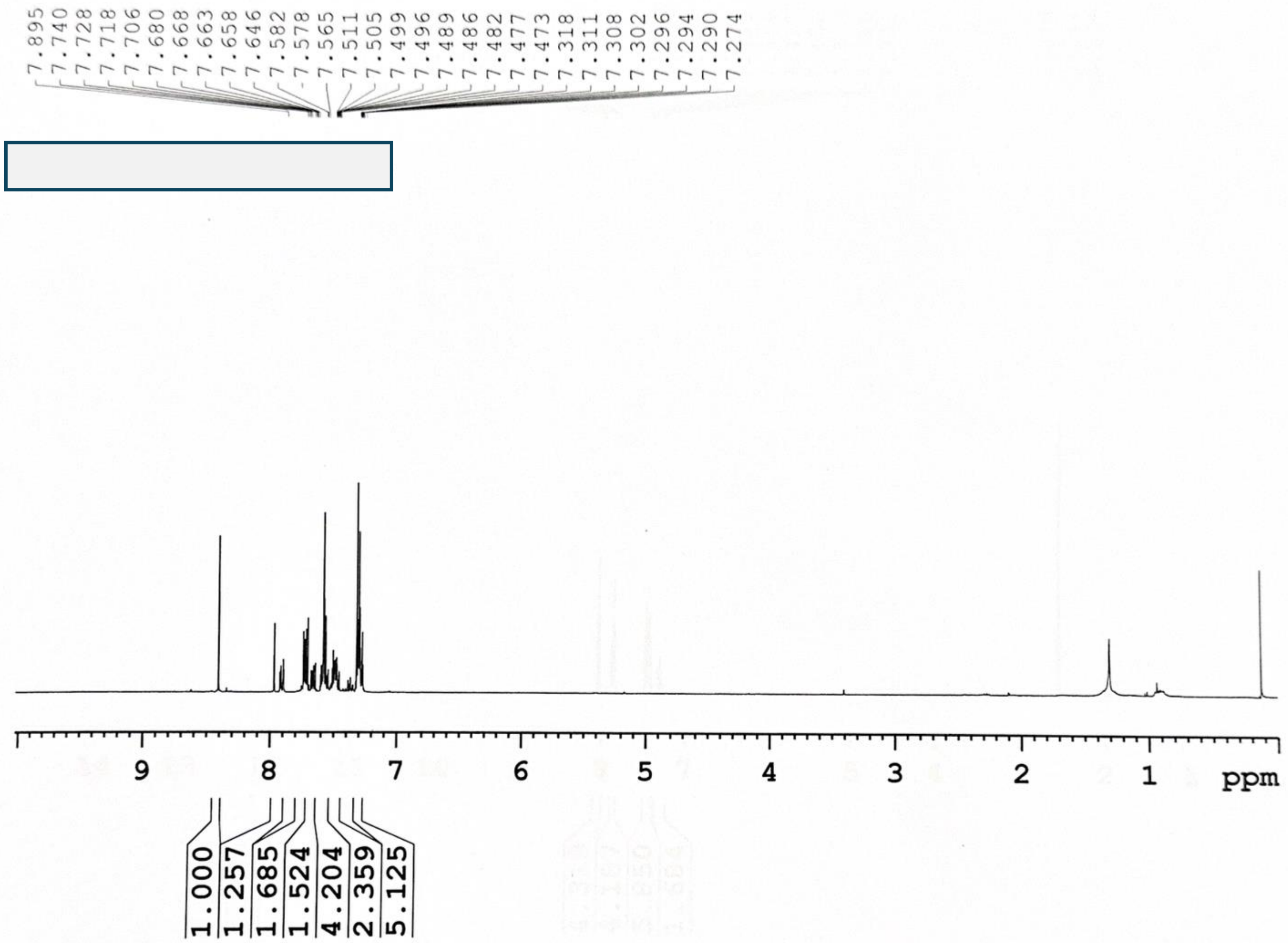
7.895
7.740
7.728
7.718
7.706
7.680
7.668
7.663
7.658
7.646
7.582
7.578
7.565
7.511
7.505
7.499
7.496
7.489
7.486
7.482
7.477
7.473
7.318
7.311
7.308
7.302
7.296
7.294
7.290
7.274
9
8
7
6
5
4
3
2
1
ppm
1.000
1.257
1.685
1.524
4.204
2.359
5.125

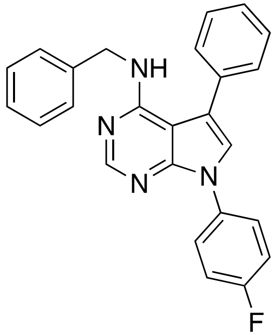
NH
N
N
N
F

6c

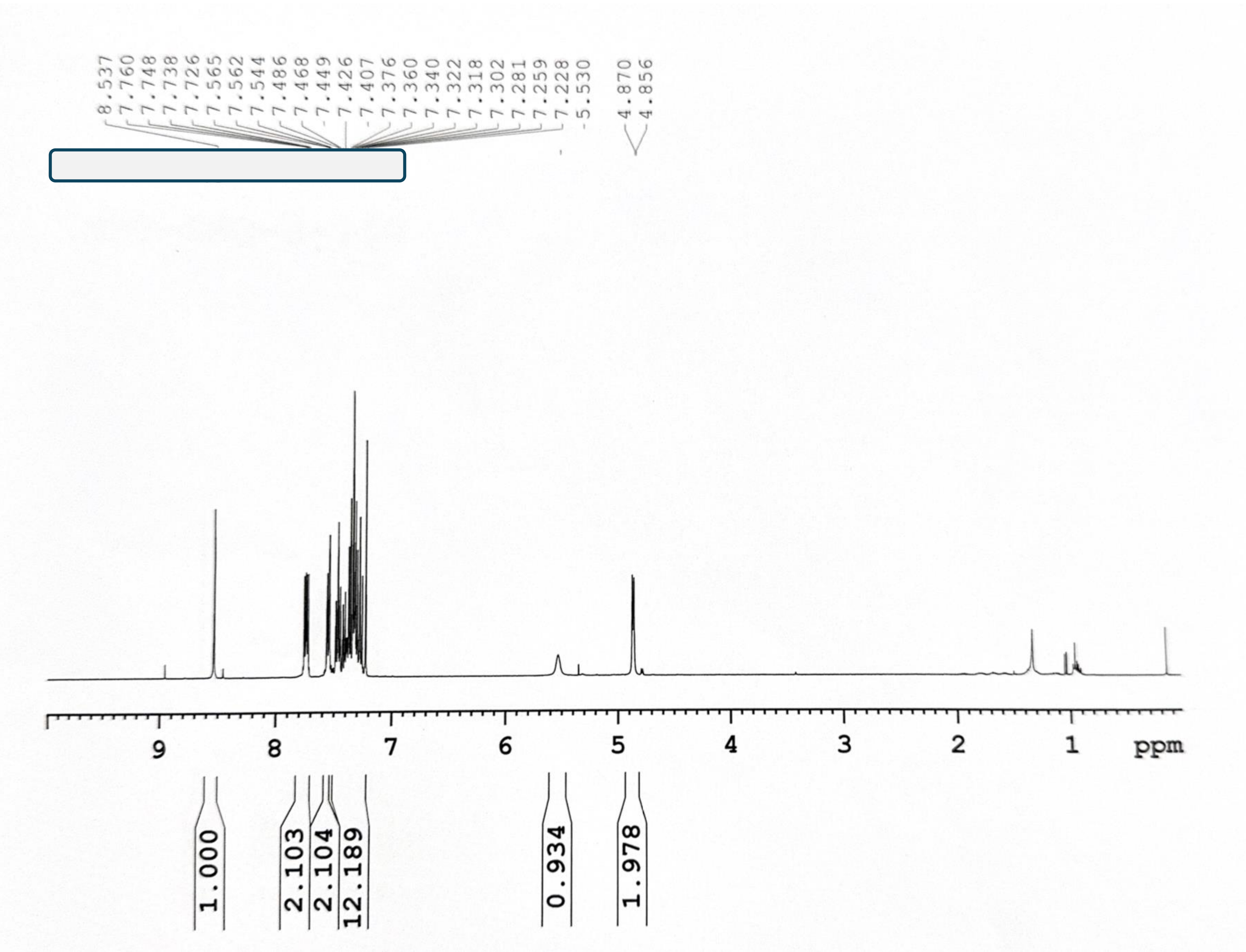
8.537
7.760
7.748
7.738
7.726
7.565
7.562
7.544
7.486
7.468
7.449
7.426
7.407
7.376
7.360
7.340
7.322
7.318
7.302
7.281
7.259
7.228
5.530
4.870
4.856
9
8
7
6
5
4
3
2
1
ppm
1.000
2.103
2.104
12.189
0.934
1.978

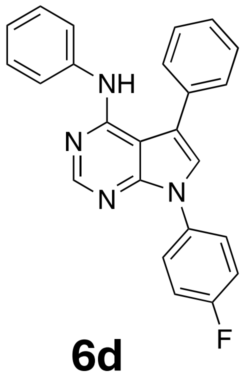
NH
N
N
N
F

6d

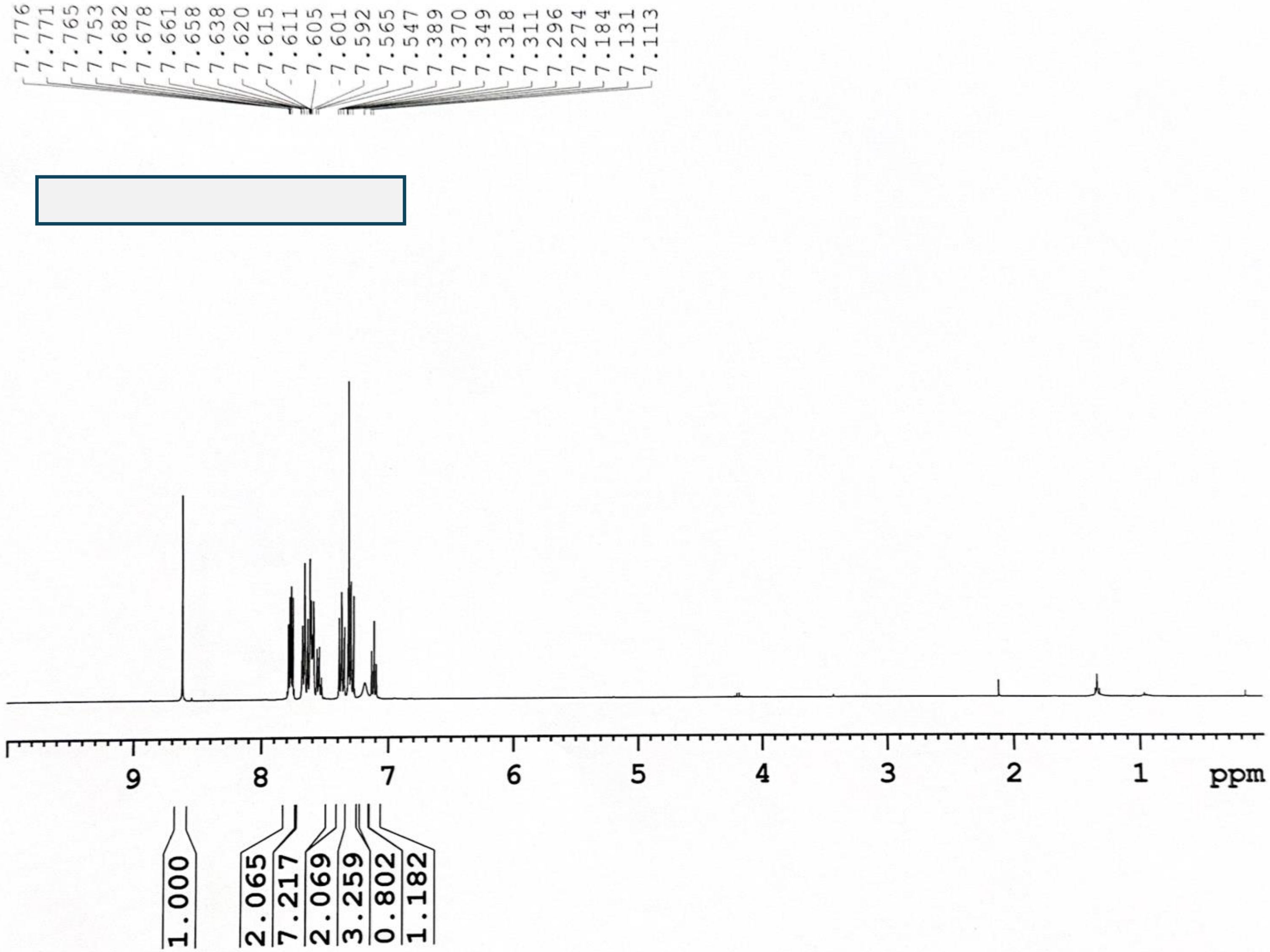
7.776
7.771
7.765
7.753
7.682
7.678
7.661
7.658
7.638
7.620
7.615
7.611
7.605
7.601
7.592
7.565
7.547
7.389
7.370
7.349
7.318
7.311
7.296
7.274
7.184
7.131
7.113
9
8
7
6
5
4
3
2
1
ppm
1.000
2.065
7.217
2.069
3.259
0.802
1.182

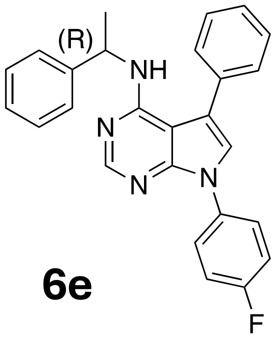
(R)
NH
N
N
N
F

6e

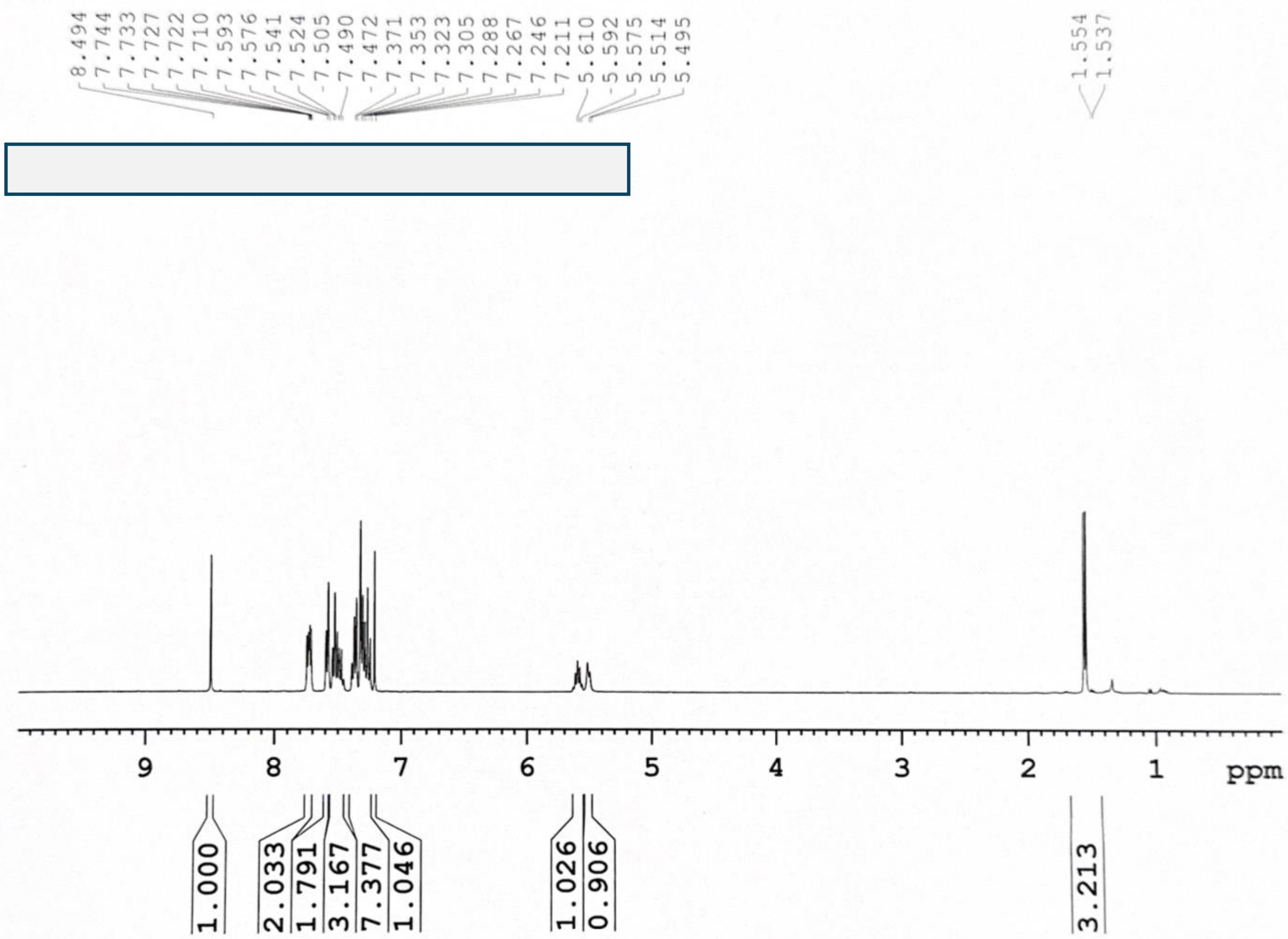
8.494
7.744
7.733
7.727
7.722
7.710
7.593
7.576
7.541
7.524
7.505
7.490
7.472
7.371
7.353
7.323
7.305
7.288
7.267
7.246
7.211
5.610
5.592
5.575
5.514
5.495
1.554
1.537
9
8
7
6
5
4
3
2
1
ppm
1.000
2.033
1.791
3.167
7.377
1.046
1.026
0.906
3.213

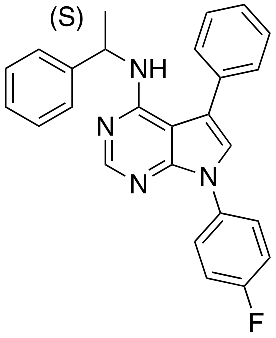
(S)
NH
N
N
N
F

6f

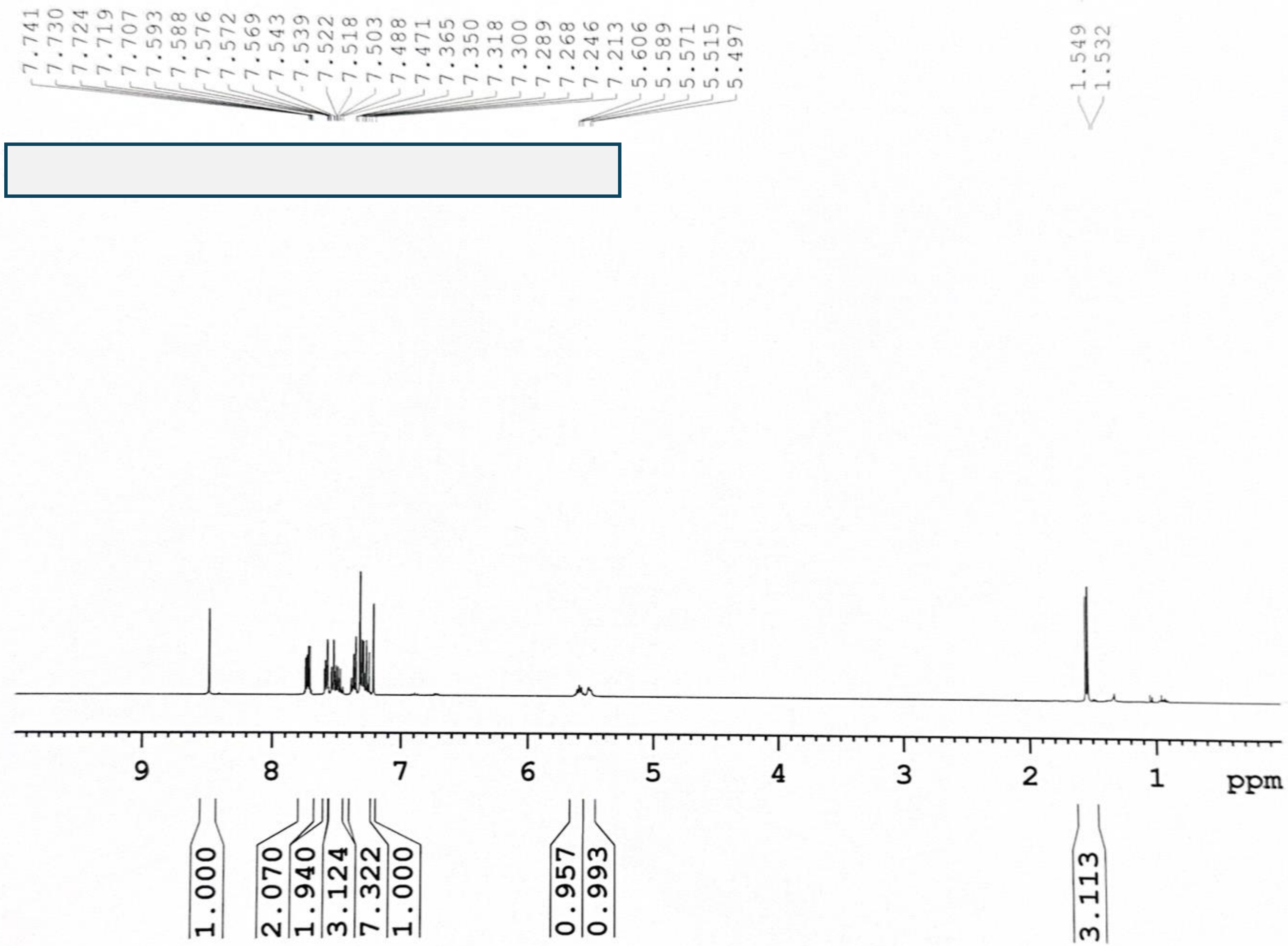
7.741
7.730
7.724
7.719
7.707
7.593
7.588
7.576
7.572
7.569
7.543
7.539
7.522
7.518
7.503
7.488
7.471
7.365
7.350
7.318
7.300
7.289
7.268
7.246
7.213
5.606
5.589
5.571
5.515
5.497
1.549
1.532
9
8
7
6
5
4
3
2
1
ppm
1.000
2.070
1.940
3.124
7.322
1.000
0.957
0.993
3.113

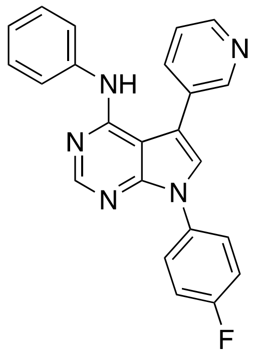
NH
N
N
N
N
N
F

6g

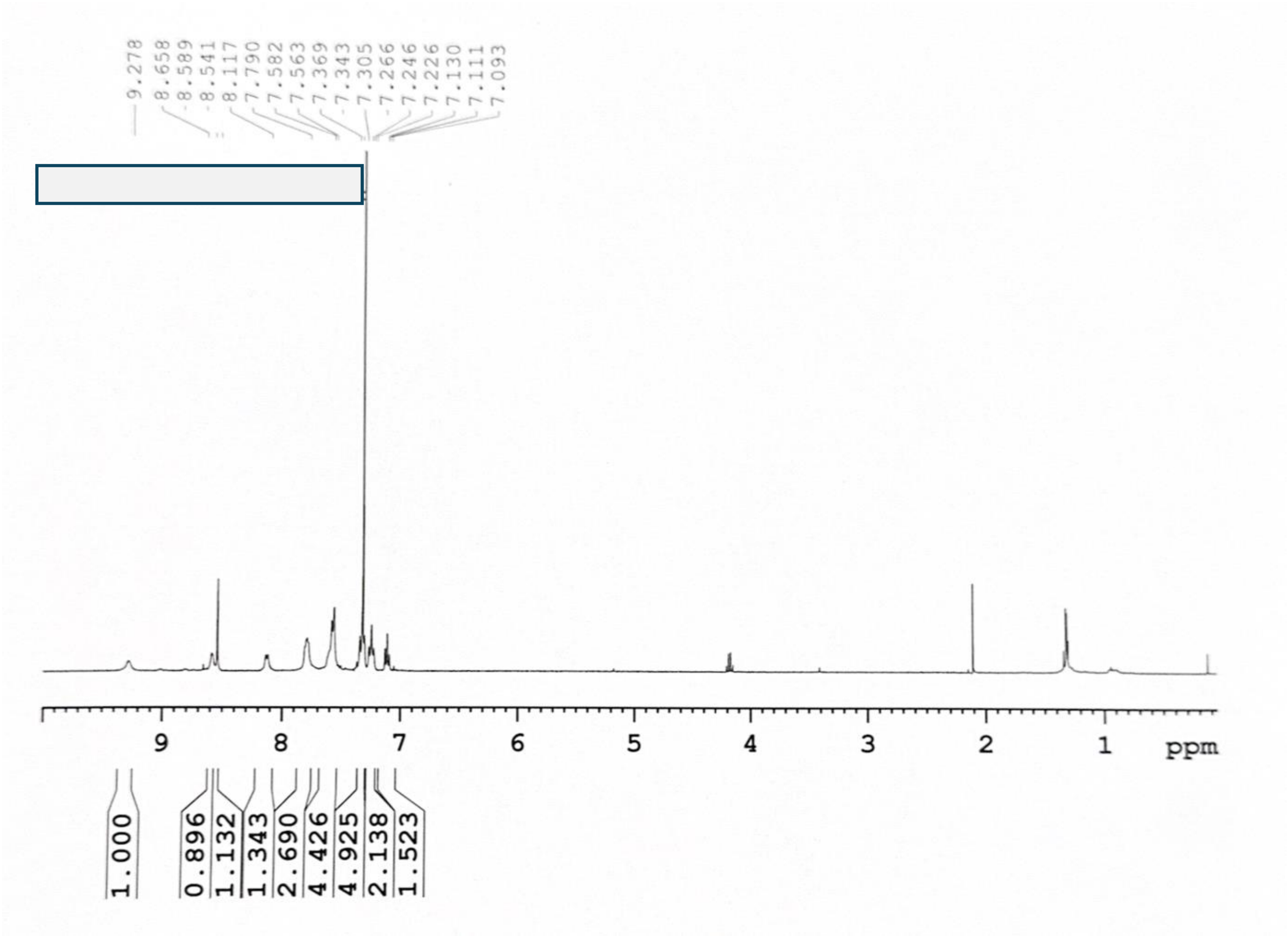
9.278
8.658
8.589
8.541
8.117
7.790
7.582
7.563
7.369
7.343
7.305
7.266
7.246
7.226
7.130
7.111
7.093
9
8
7
6
5
4
3
2
1
ppm
1.000
0.896
1.132
1.343
2.690
4.426
4.925
2.138
1.523

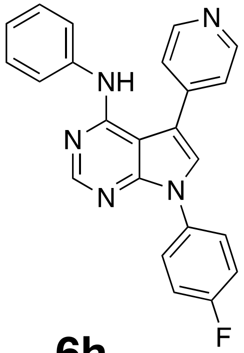
N
NH
N
N
N
F

6h

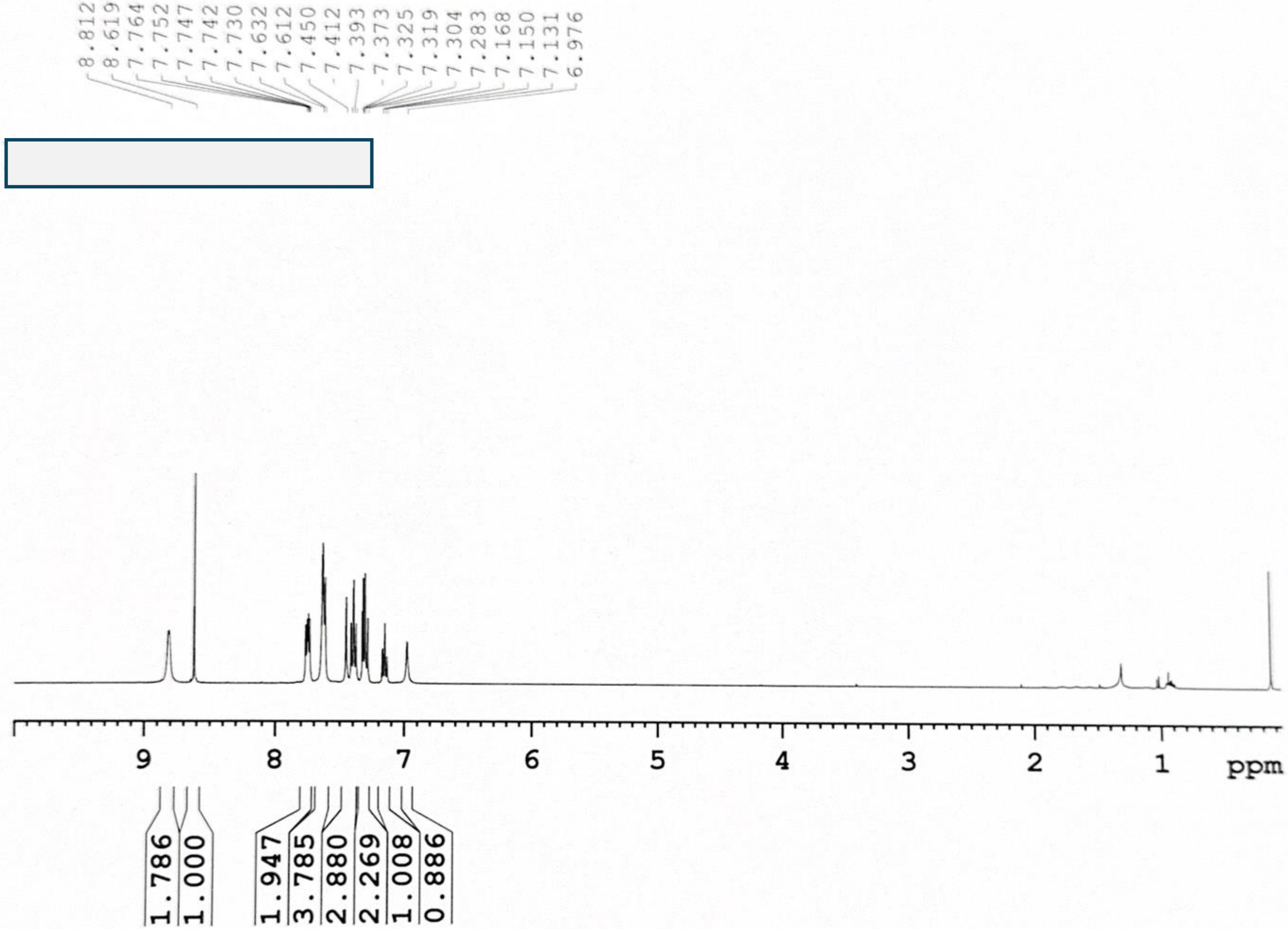
8.812
8.619
7.764
7.752
7.747
7.742
7.730
7.632
7.612
7.450
7.412
7.393
7.373
7.325
7.319
7.304
7.283
7.168
7.150
7.131
6.976
9
8
7
6
5
4
3
2
1
ppm
1.786
1.000
1.947
3.785
2.880
2.269
1.008
0.886

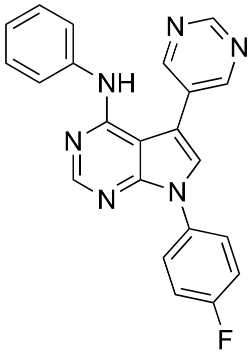
NH
N
N
N
N
N
F

6i

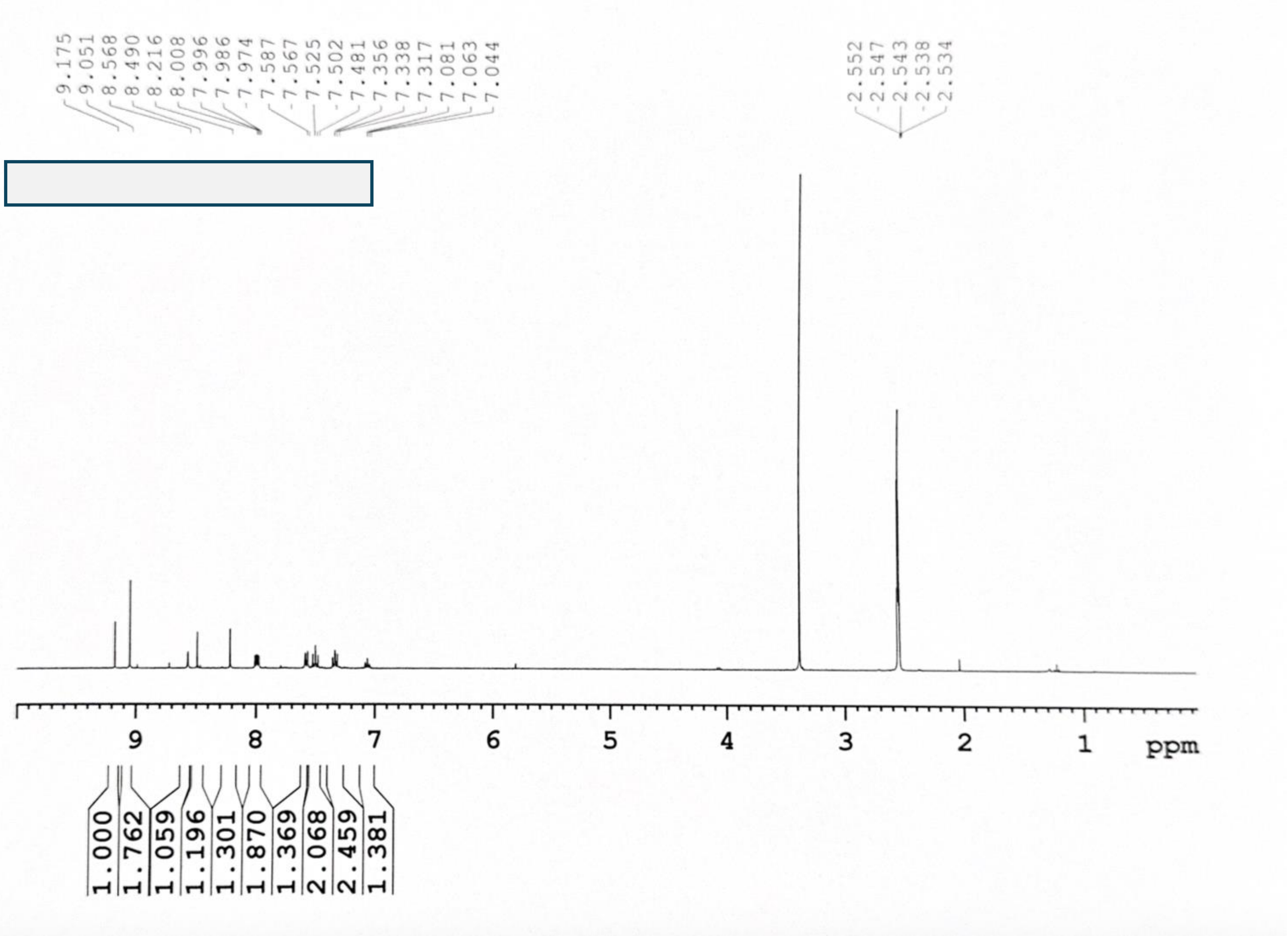
9.175
9.051
8.568
8.490
8.216
8.008
7.996
7.986
7.974
7.587
7.567
7.525
7.502
7.481
7.356
7.338
7.317
7.081
7.063
7.044
2.552
2.547
2.543
2.538
2.534
9
8
7
6
5
4
3
2
1
ppm
1.000
1.762
1.059
1.196
1.301
1.870
1.369
2.068
2.459
1.381

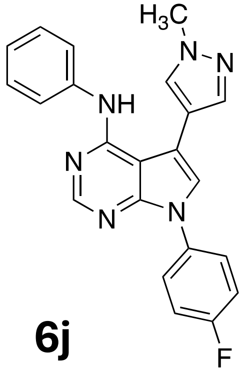

H3C
N
N
NH
N
N
N
F

6j

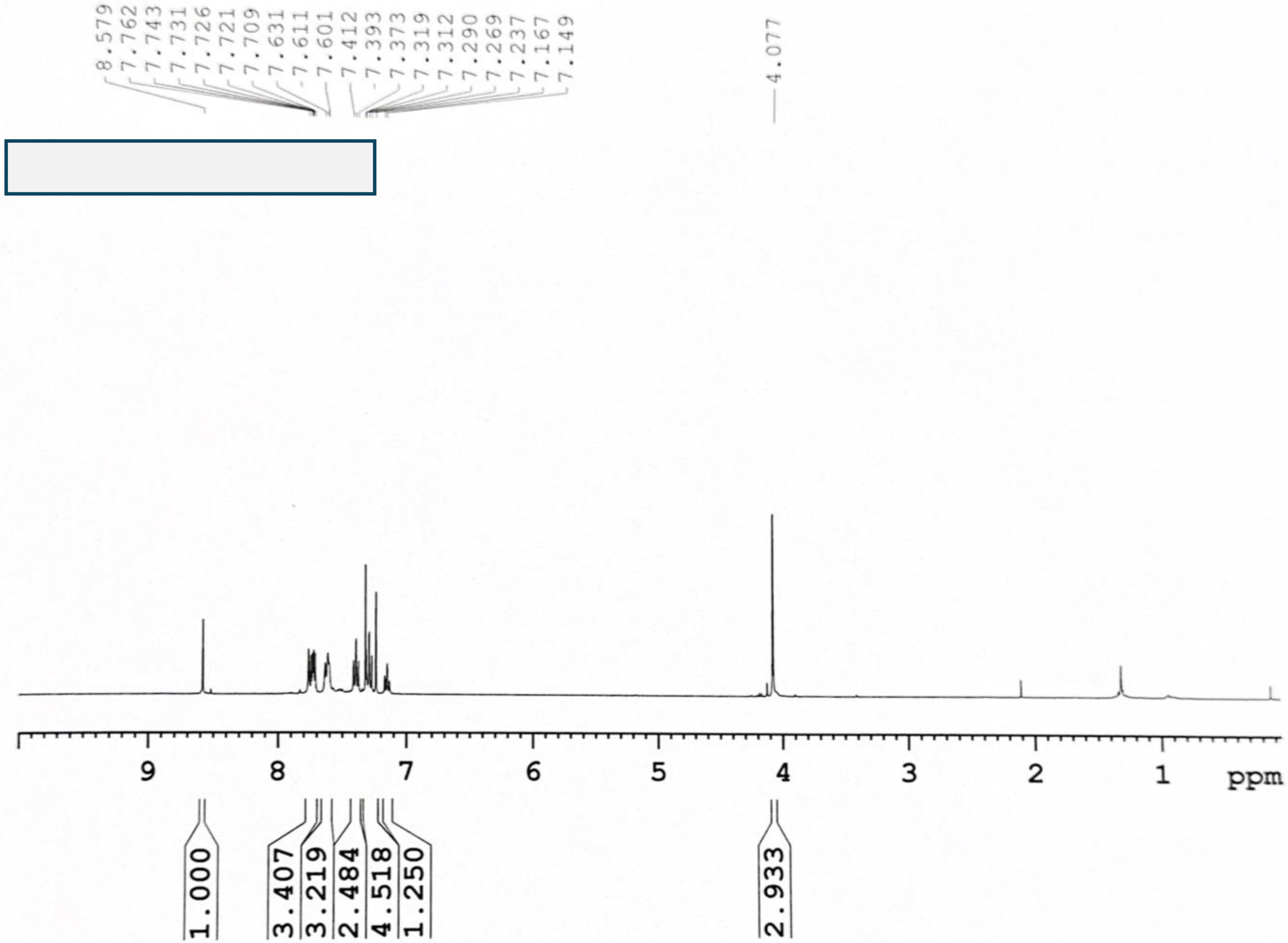

8.579
7.762
7.743
7.731
7.726
7.721
7.709
7.631
7.611
7.601
7.412
7.393
7.373
7.319
7.312
7.290
7.269
7.237
7.167
7.149
4.077
9
8
7
6
5
4
3
2
1
ppm
1.000
3.407
3.219
2.484
4.518
1.250
2.933

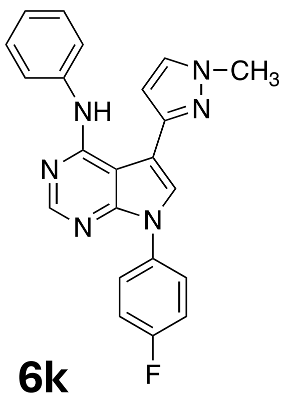
NH
N−CH3
N
N
N
N
F

6k

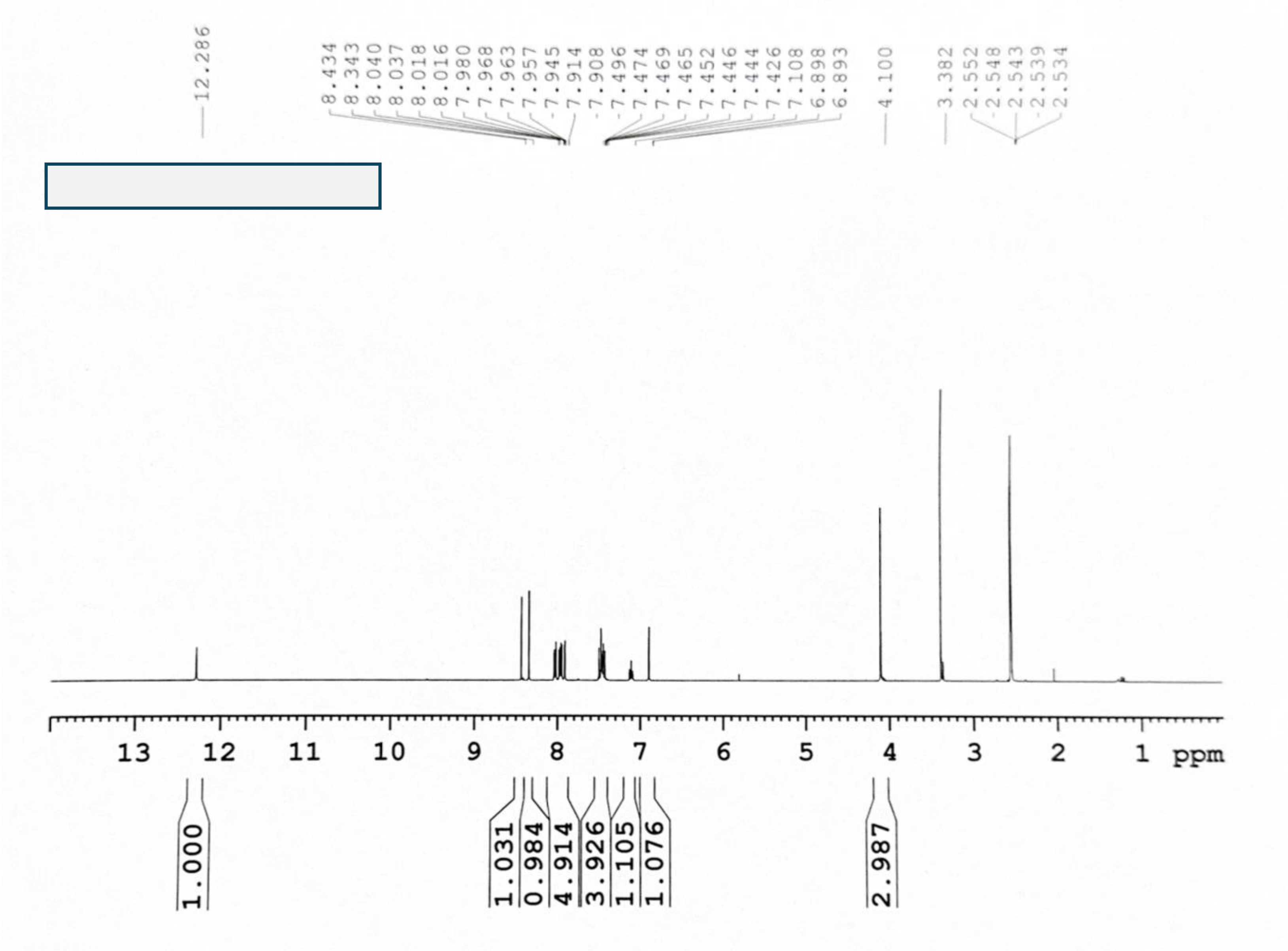
12.286
8.434
8.343
8.040
8.037
8.018
8.016
7.980
7.968
7.963
7.957
7.945
7.914
7.908
7.496
7.474
7.469
7.465
7.452
7.446
7.444
7.426
7.108
6.898
6.893
4.100
3.382
2.552
2.548
2.543
2.539
2.534
13
12
11
10
9
8
7
6
5
4
3
2
1
ppm
1.000
1.031
0.984
4.914
3.926
1.105
1.076
2.987

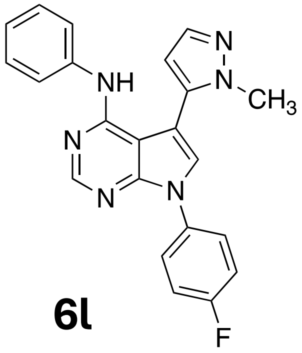
NH
N
N
N
N
N
CH3
F

6l

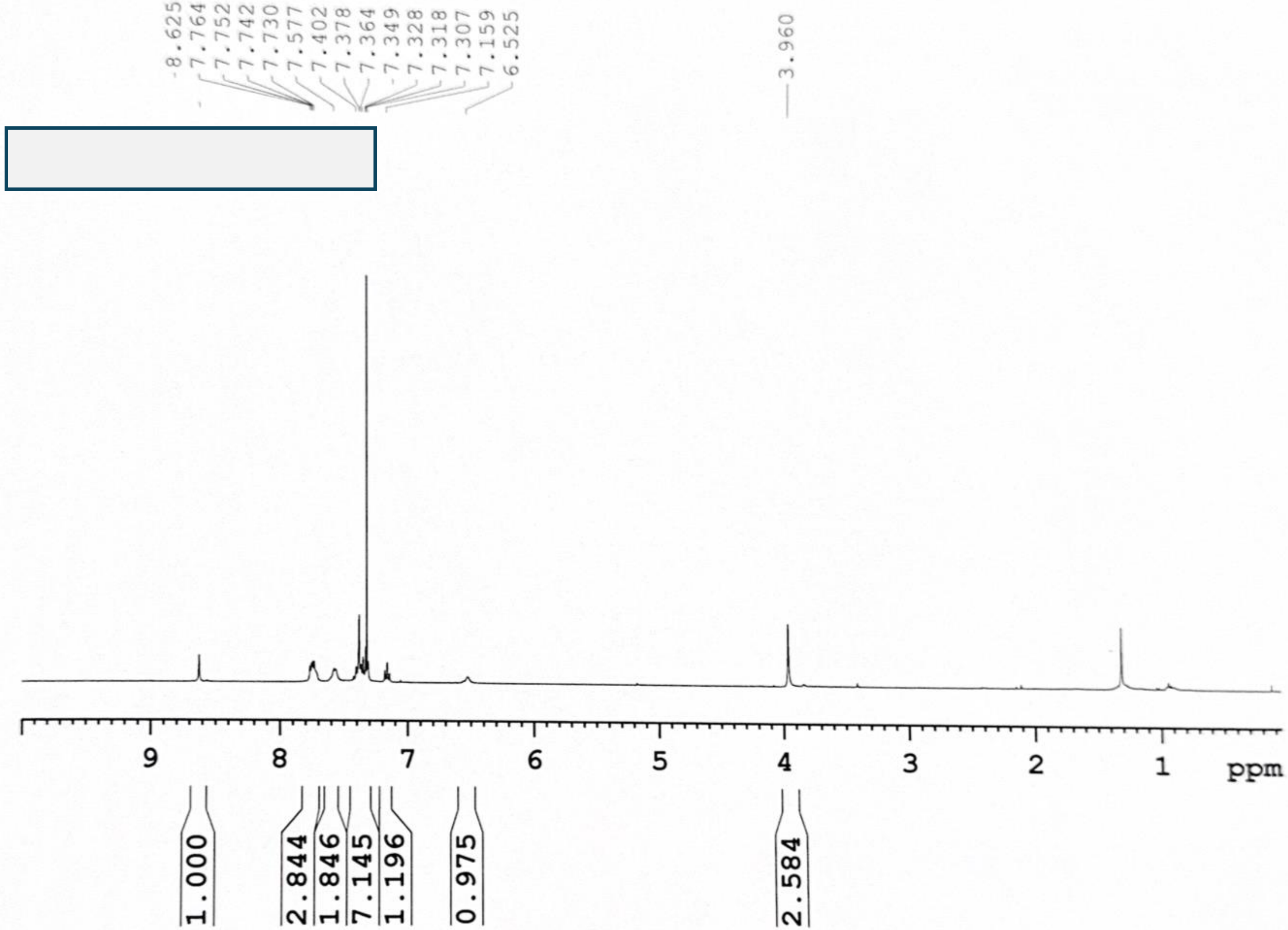
8.625
7.764
7.752
7.742
7.730
7.577
7.402
7.378
7.364
7.349
7.328
7.318
7.307
7.159
6.525
3.960
9
8
7
6
5
4
3
2
1
ppm
1.000
2.844
1.846
7.145
1.196
0.975
2.584

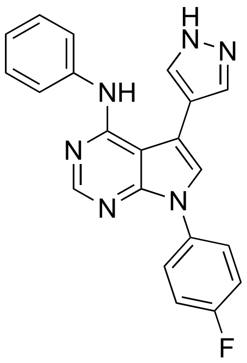

H
N
N
NH
N
N
N
F

6
m

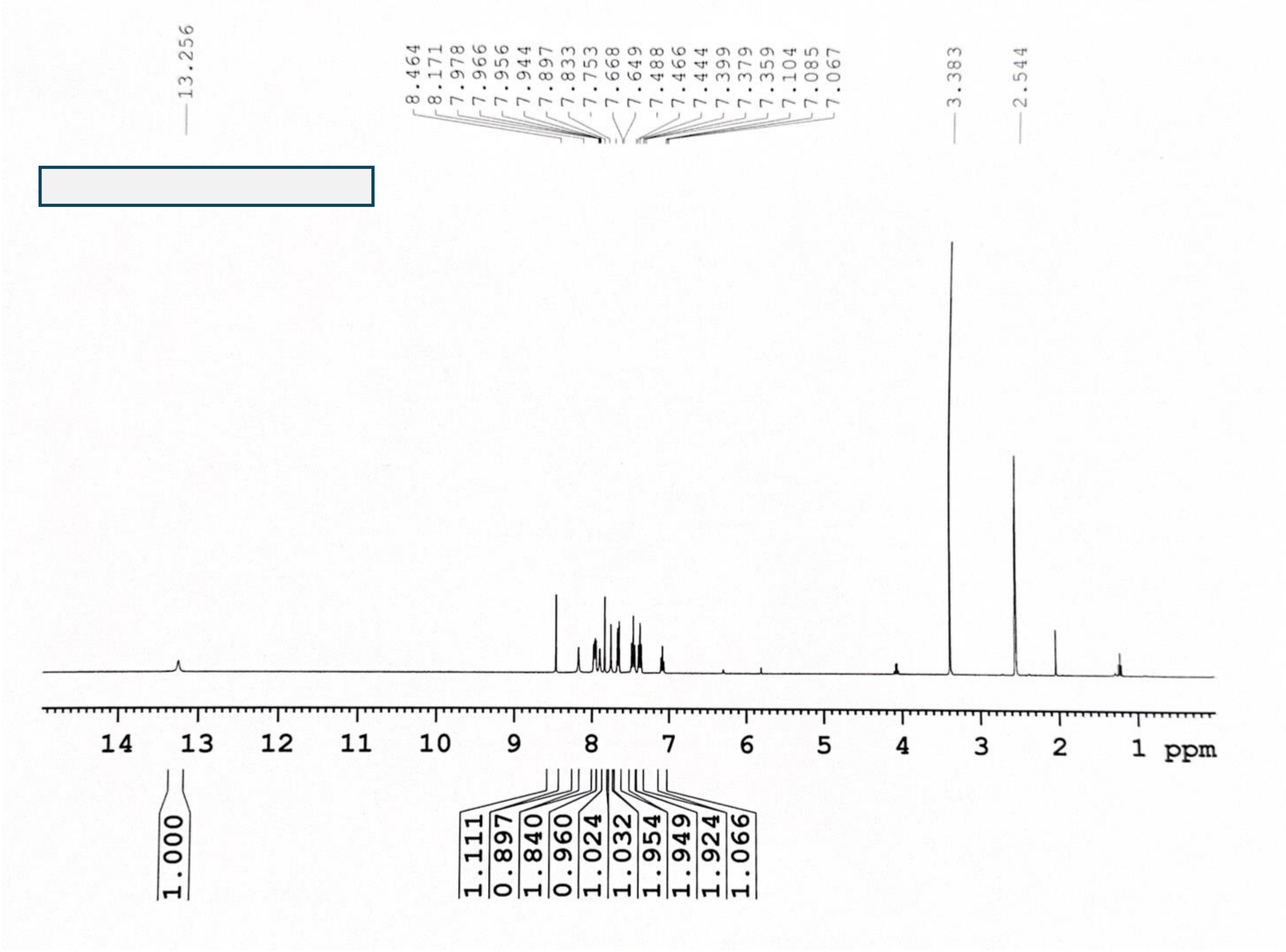

13.256
8.464
8.171
7.978
7.966
7.956
7.944
7.897
7.833
7.753
7.668
7.649
7.488
7.466
7.444
7.399
7.379
7.359
7.104
7.085
7.067
3.383
2.544
14
13
12
11
10
9
8
7
6
5
4
3
2
1 ppm
1.000
1.111
0.897
1.840
0.960
1.024
1.032
1.954
1.949
1.924
1.066

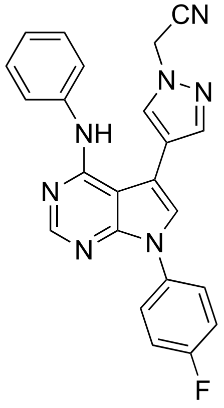
CN
N
N
NH
N
N
N
F

6n

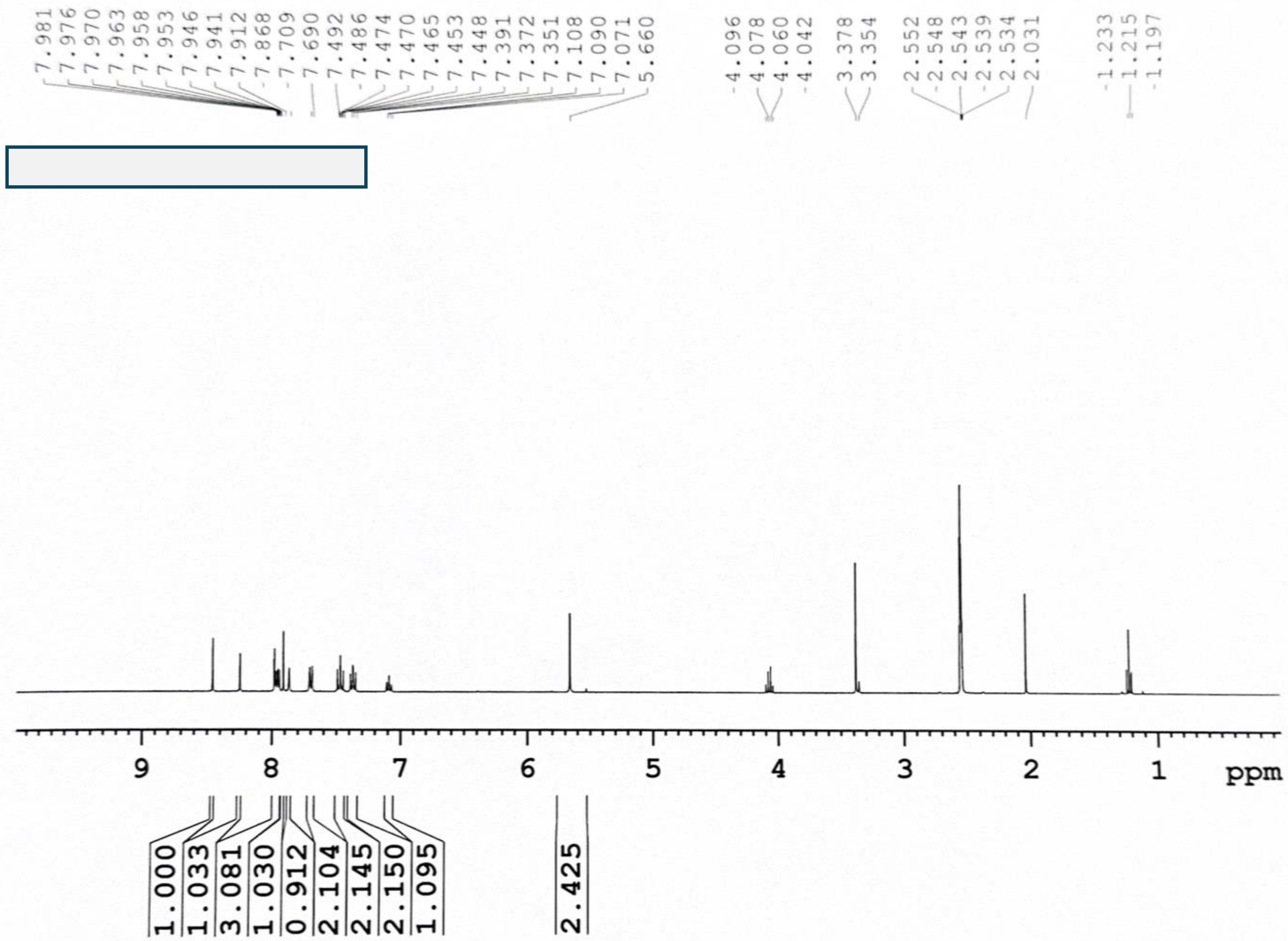
7.981
7.976
7.970
7.963
7.958
7.953
7.946
7.941
7.912
7.868
7.709
7.690
7.492
7.486
7.474
7.470
7.465
7.453
7.448
7.391
7.372
7.351
7.108
7.090
7.071
5.660
4.096
4.078
4.060
4.042
3.378
3.354
2.552
2.548
2.543
2.539
2.534
2.031
1.233
1.215
1.197
9
8
7
6
5
4
3
2
1
ppm
1.000
1.033
3.081
1.030
0.912
2.104
2.145
2.150
1.095
2.425

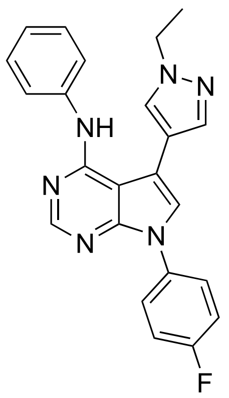

NH
N
N
N
N
N
F

6o

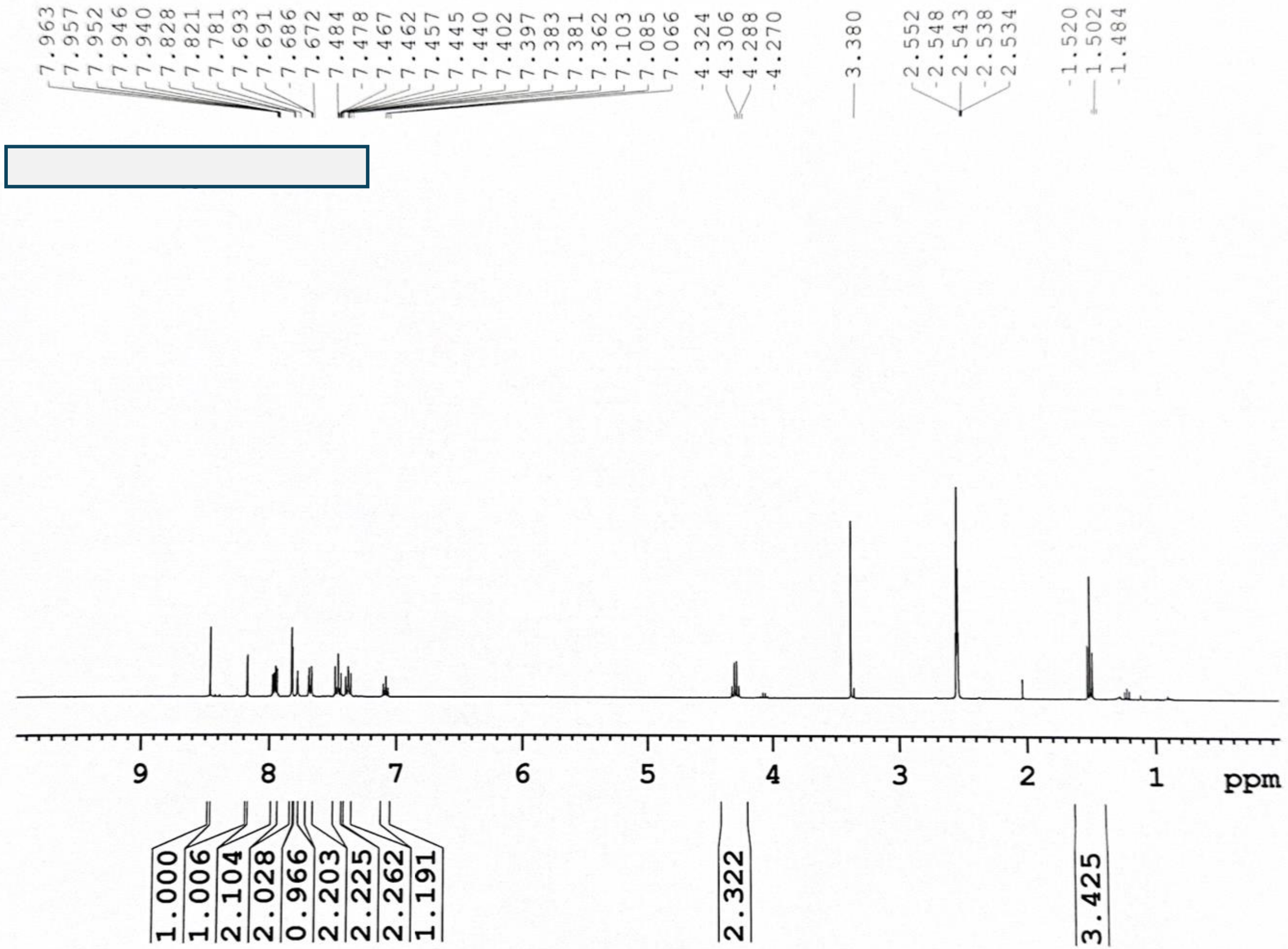

7.963
7.957
7.952
7.946
7.940
7.828
7.821
7.781
7.693
7.691
7.686
7.672
7.484
7.478
7.467
7.462
7.457
7.445
7.440
7.402
7.397
7.383
7.381
7.362
7.103
7.085
7.066
4.324
4.306
4.288
4.270
3.380
2.552
2.548
2.543
2.538
2.534
1.520
1.502
1.484
9
8
7
6
5
4
3
2
1
ppm
1.000
1.006
2.104
2.028
0.966
2.203
2.225
2.262
1.191
2.322
3.425

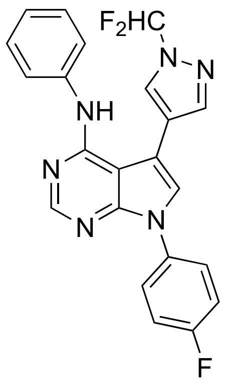
F2HC
N
N
NH
N
N
N
F

6p

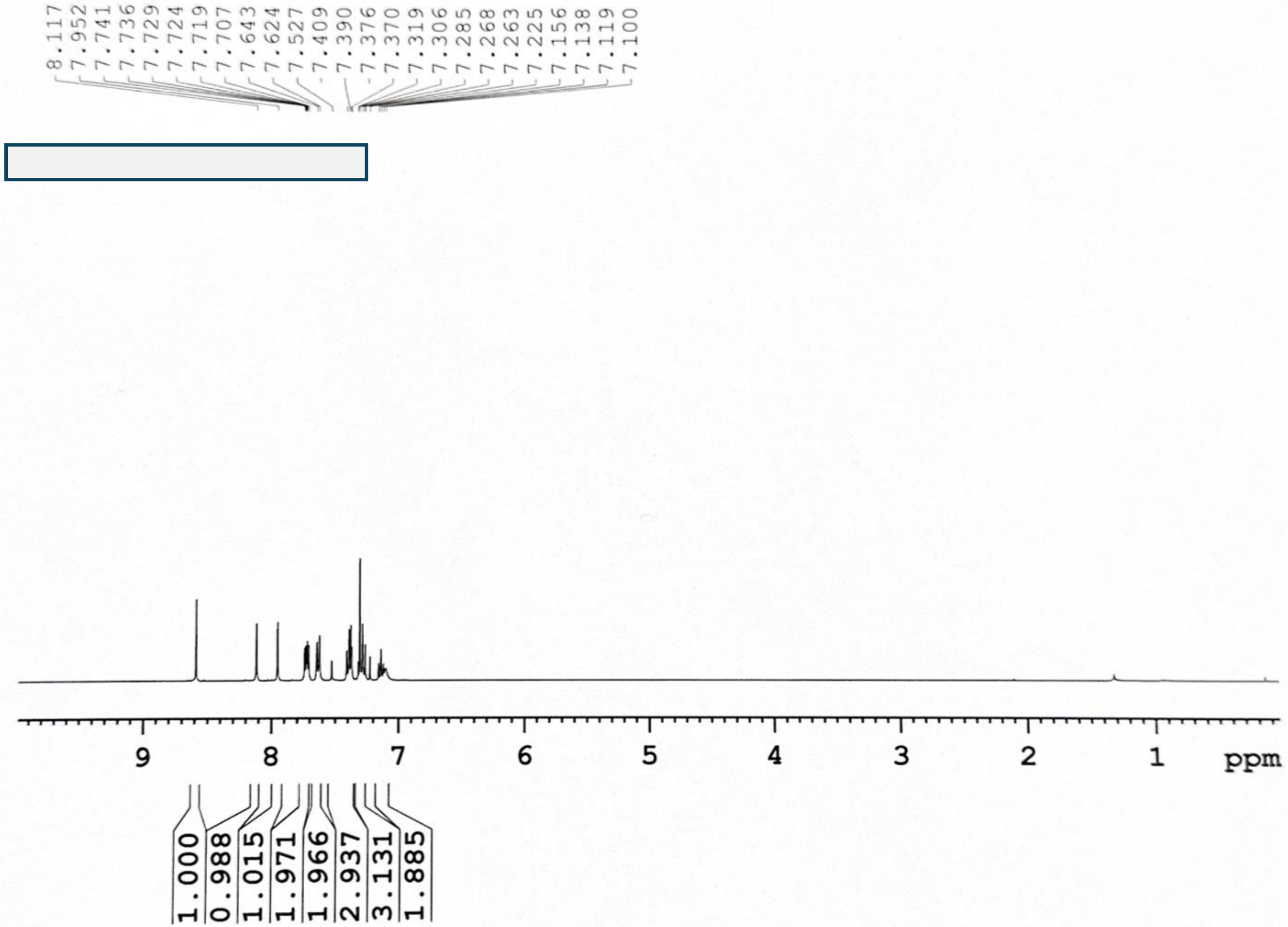
8.117
7.952
7.741
7.736
7.729
7.724
7.719
7.707
7.643
7.624
7.527
7.409
7.390
7.376
7.370
7.319
7.306
7.285
7.268
7.263
7.225
7.156
7.138
7.119
7.100
9
8
7
6
5
4
3
2
1
ppm
1.000
0.988
1.015
1.971
1.966
2.937
3.131
1.885

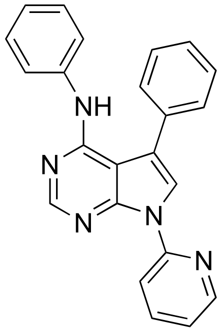

10a

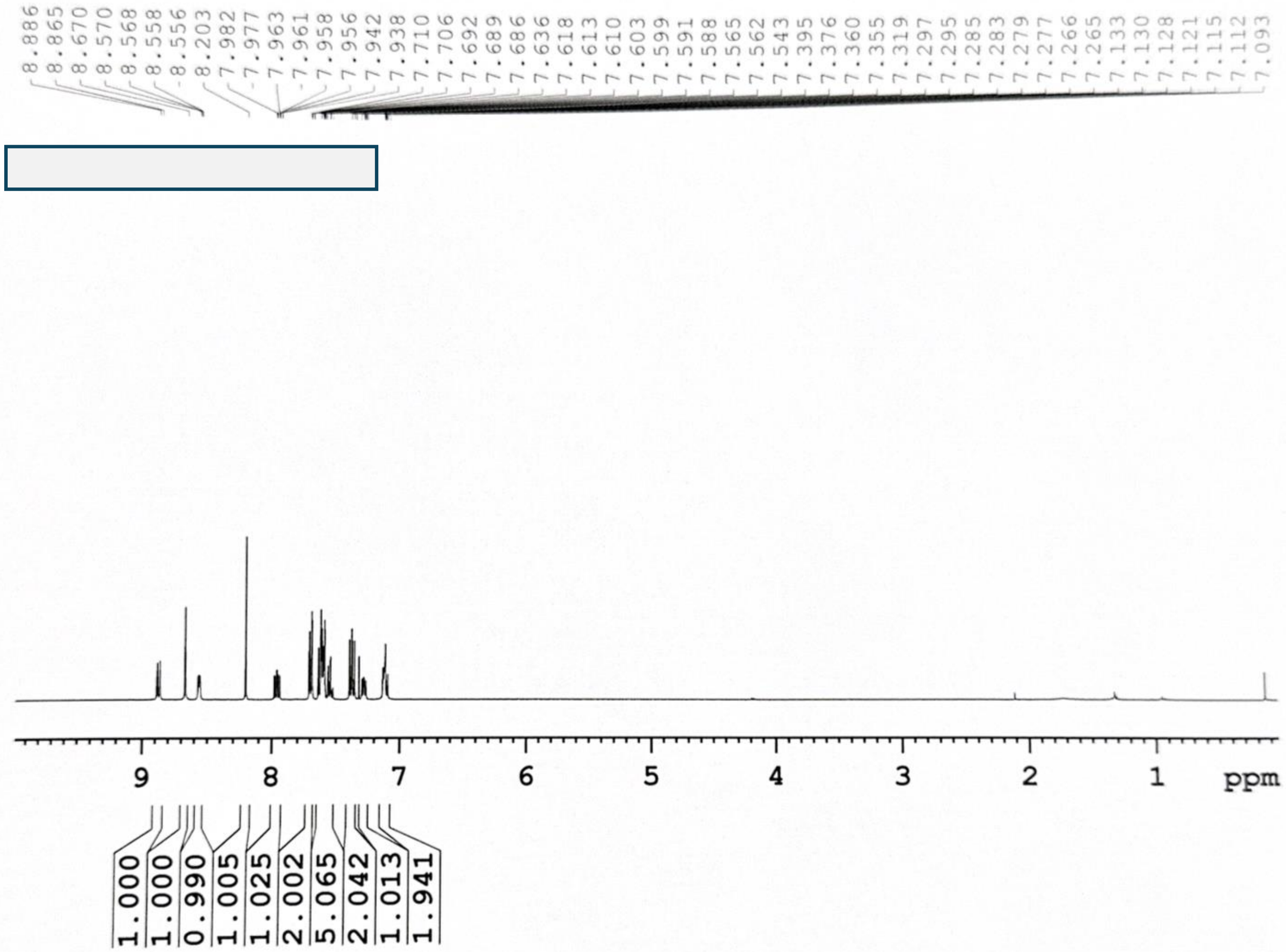
8.886
8.865
8.670
8.570
8.568
8.558
8.556
8.203
7.982
7.977
7.963
7.961
7.958
7.956
7.942
7.938
7.710
7.706
7.692
7.689
7.686
7.636
7.618
7.613
7.610
7.603
7.599
7.591
7.588
7.565
7.562
7.543
7.395
7.376
7.360
7.355
7.319
7.297
7.295
7.285
7.283
7.279
7.277
7.266
7.265
7.133
7.130
7.128
7.121
7.115
7.112
7.093
9
8
7
6
5
4
3
2
1
ppm
1.000
1.000
0.990
1.005
1.025
2.002
5.065
2.042
1.013
1.941

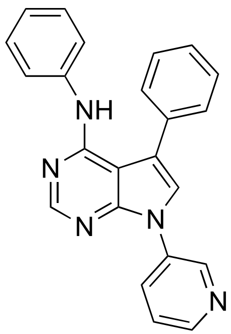

10b

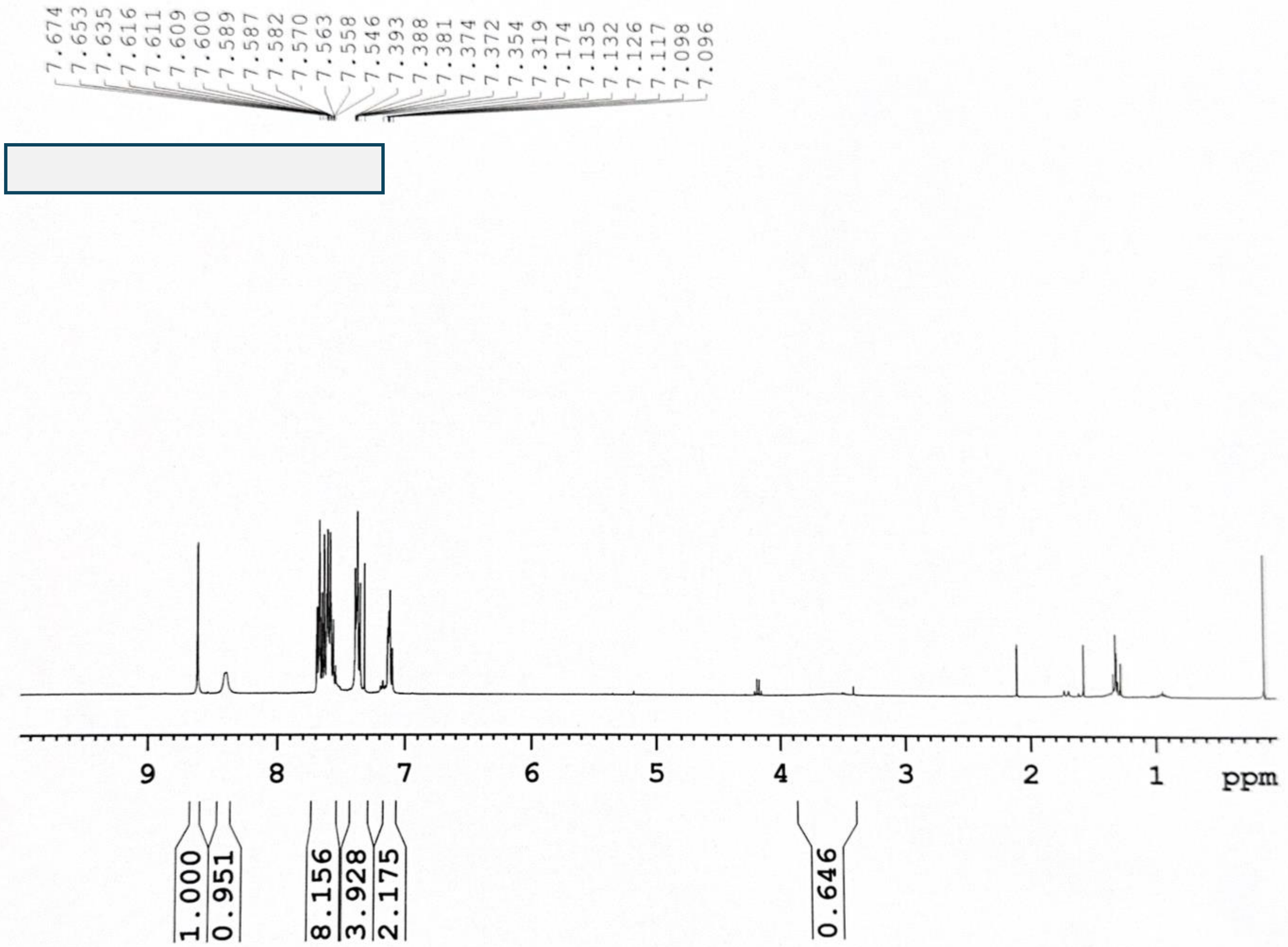
7.674
7.653
7.635
7.616
7.611
7.609
7.600
7.589
7.587
7.582
7.570
7.563
7.558
7.546
7.393
7.388
7.381
7.374
7.372
7.354
7.319
7.174
7.135
7.132
7.126
7.117
7.098
7.096
9
8
7
6
5
4
3
2
1
ppm
1.000
0.951
8.156
3.928
2.175
0.646

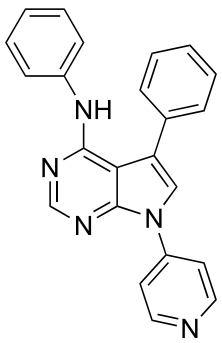
NH
N
N
N
N

10c

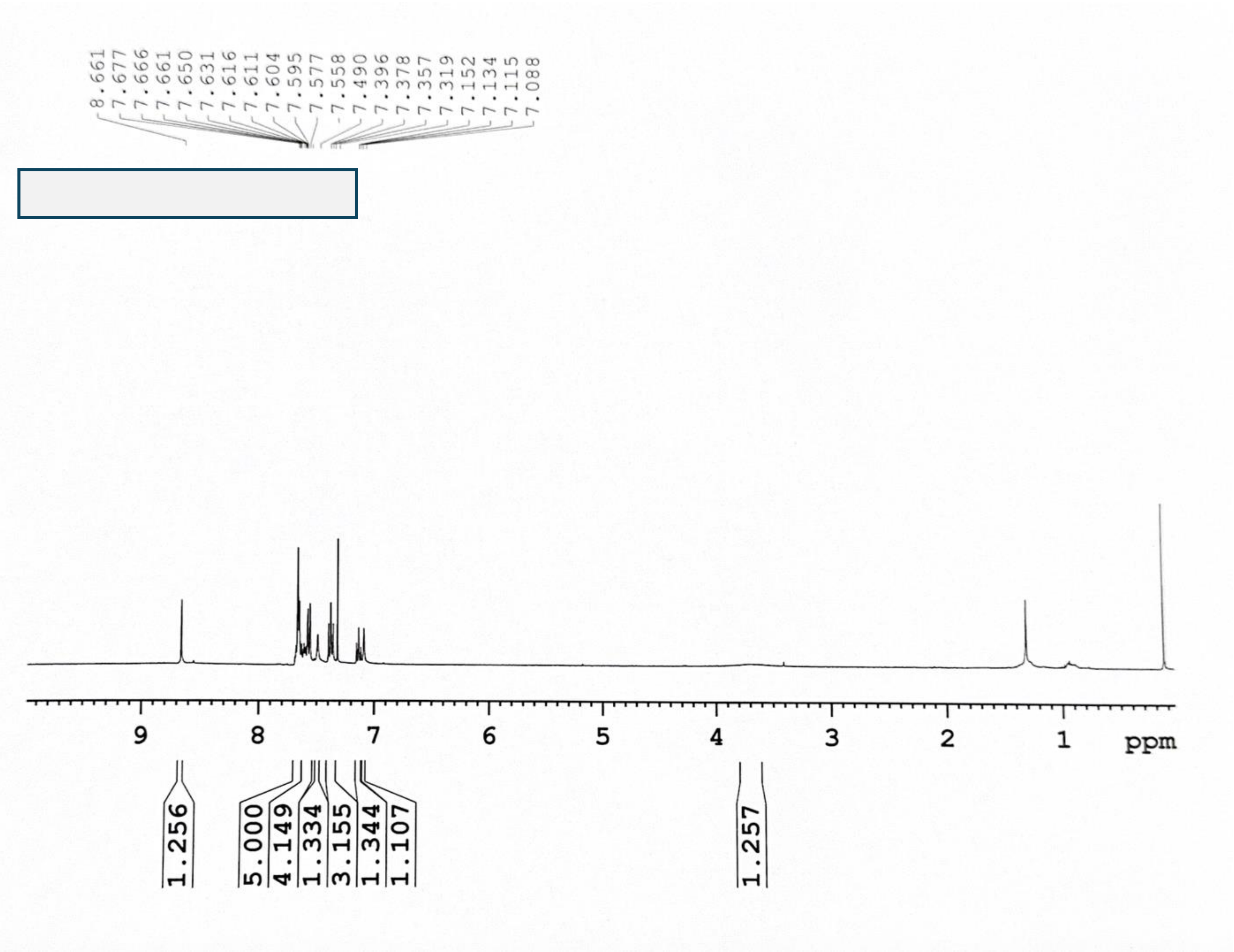
8.661
7.677
7.666
7.661
7.650
7.631
7.616
7.611
7.604
7.595
7.577
7.558
7.490
7.396
7.378
7.357
7.319
7.152
7.134
7.115
7.088
9
8
7
6
5
4
3
2
1
ppm
1.256
5.000
4.149
1.334
3.155
1.344
1.107
1.257

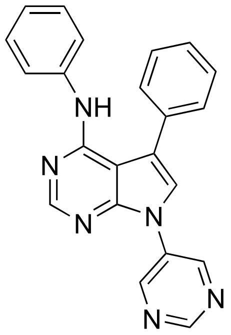

10d

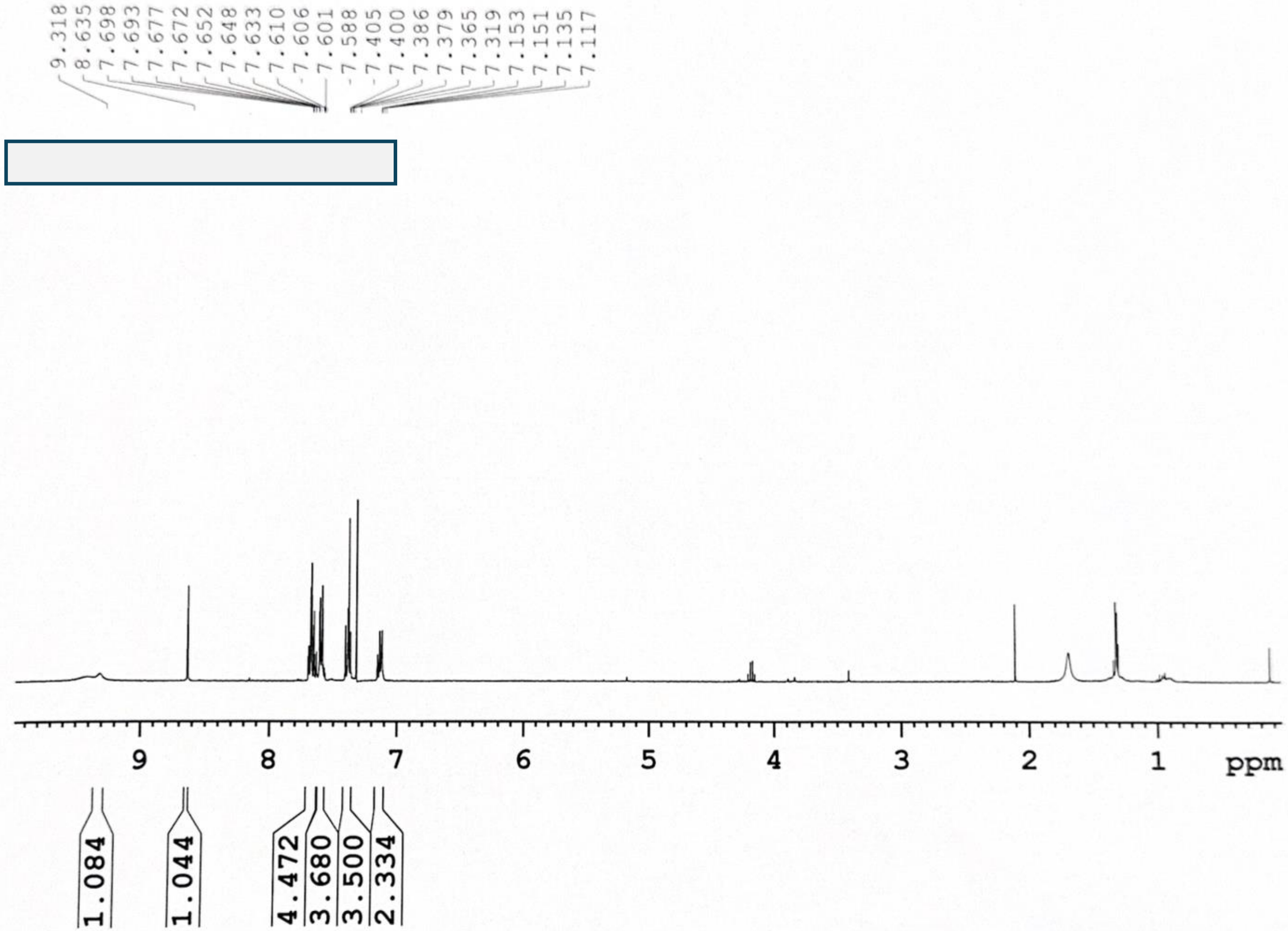
9.318
8.635
7.698
7.693
7.677
7.672
7.652
7.648
7.633
7.610
7.606
7.601
7.588
7.405
7.400
7.386
7.379
7.365
7.319
7.153
7.151
7.135
7.117
9
8
7
6
5
4
3
2
1
ppm
1.084
1.044
4.472
3.680
3.500
2.334

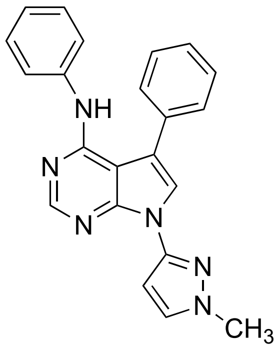
NH
N
N
N
N
N
CH3

10e

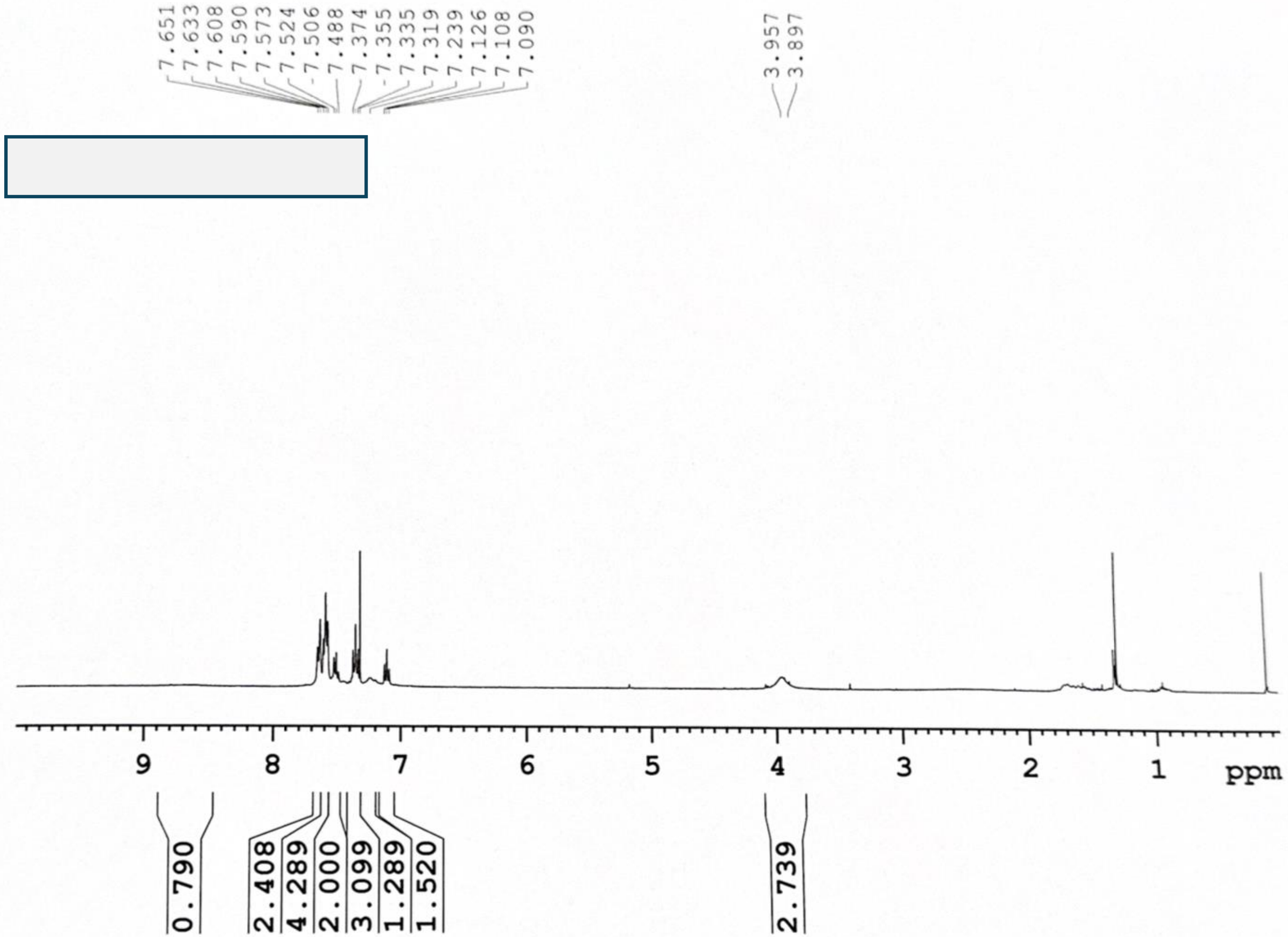
7.651
7.633
7.608
7.590
7.573
7.524
7.506
7.488
7.374
7.355
7.335
7.319
7.239
7.126
7.108
7.090
3.957
3.897
9
8
7
6
5
4
3
2
1
ppm
0.790
2.408
4.289
2.000
3.099
1.289
1.520
2.739

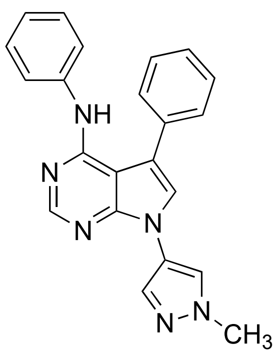
NH
N
N
N
N
N
CH3

10f

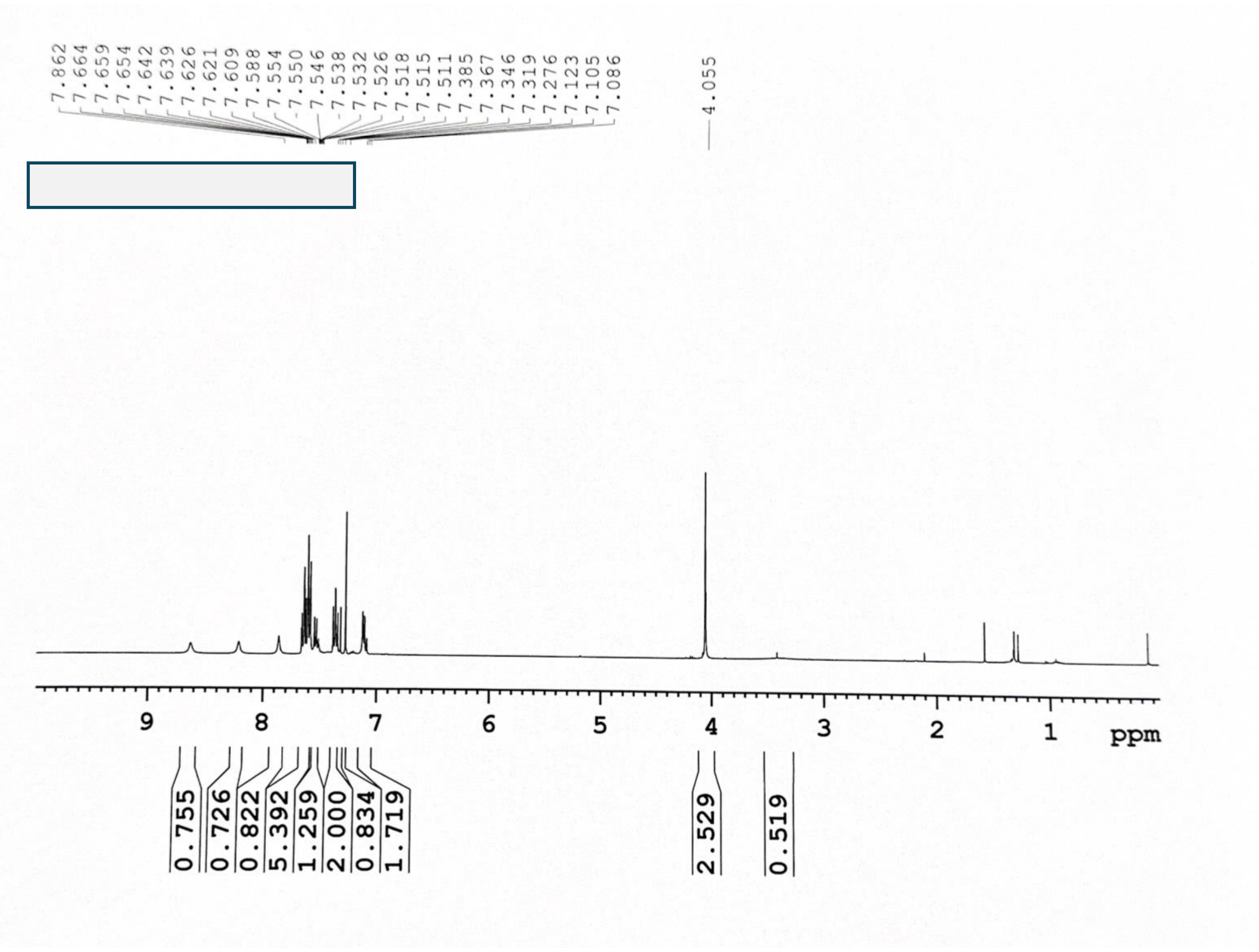
7.862
7.664
7.659
7.654
7.642
7.639
7.626
7.621
7.609
7.588
7.554
7.550
7.546
7.538
7.532
7.526
7.518
7.515
7.511
7.385
7.367
7.346
7.319
7.276
7.123
7.105
7.086
4.055
9
8
7
6
5
4
3
2
1
ppm
0.755
0.726
0.822
5.392
1.259
2.000
0.834
1.719
2.529
0.519

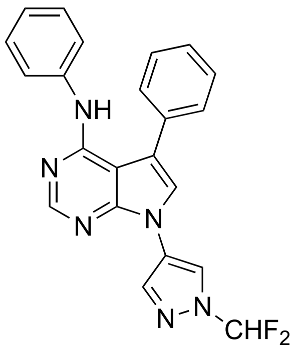

NH
N
N
N
N
N
CHF2

10g

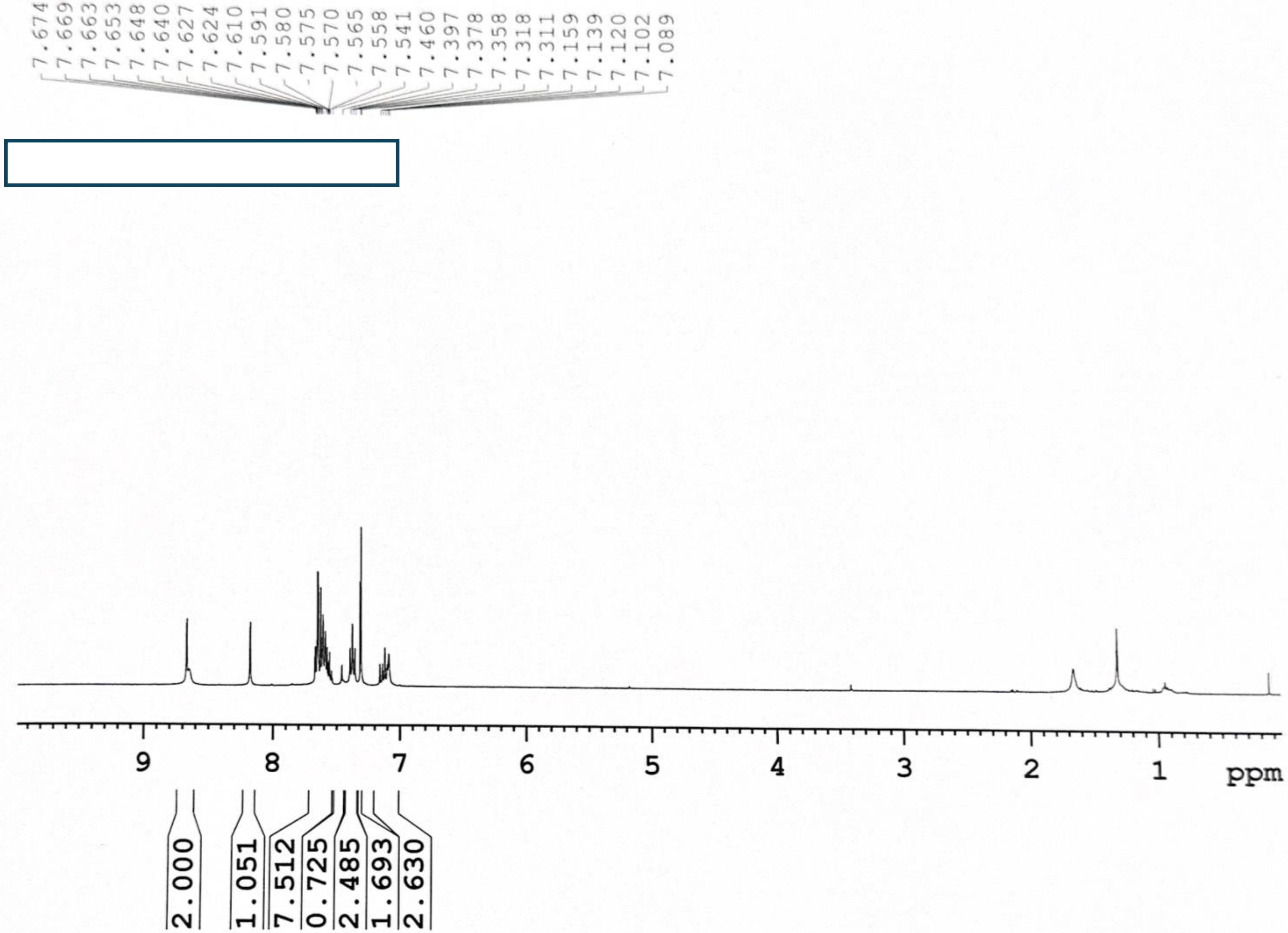

7.674
7.669
7.663
7.653
7.648
7.640
7.627
7.624
7.610
7.591
7.580
7.575
7.570
7.565
7.558
7.541
7.460
7.397
7.378
7.358
7.318
7.311
7.159
7.139
7.120
7.102
7.089
9
8
7
6
5
4
3
2
1
ppm
2.000
1.051
7.512
0.725
2.485
1.693
2.630

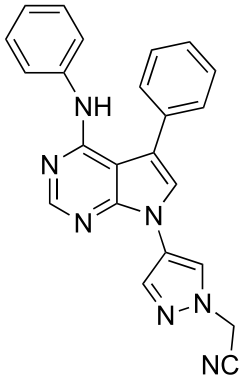
NH
N
N
N
N
N
NC

10h

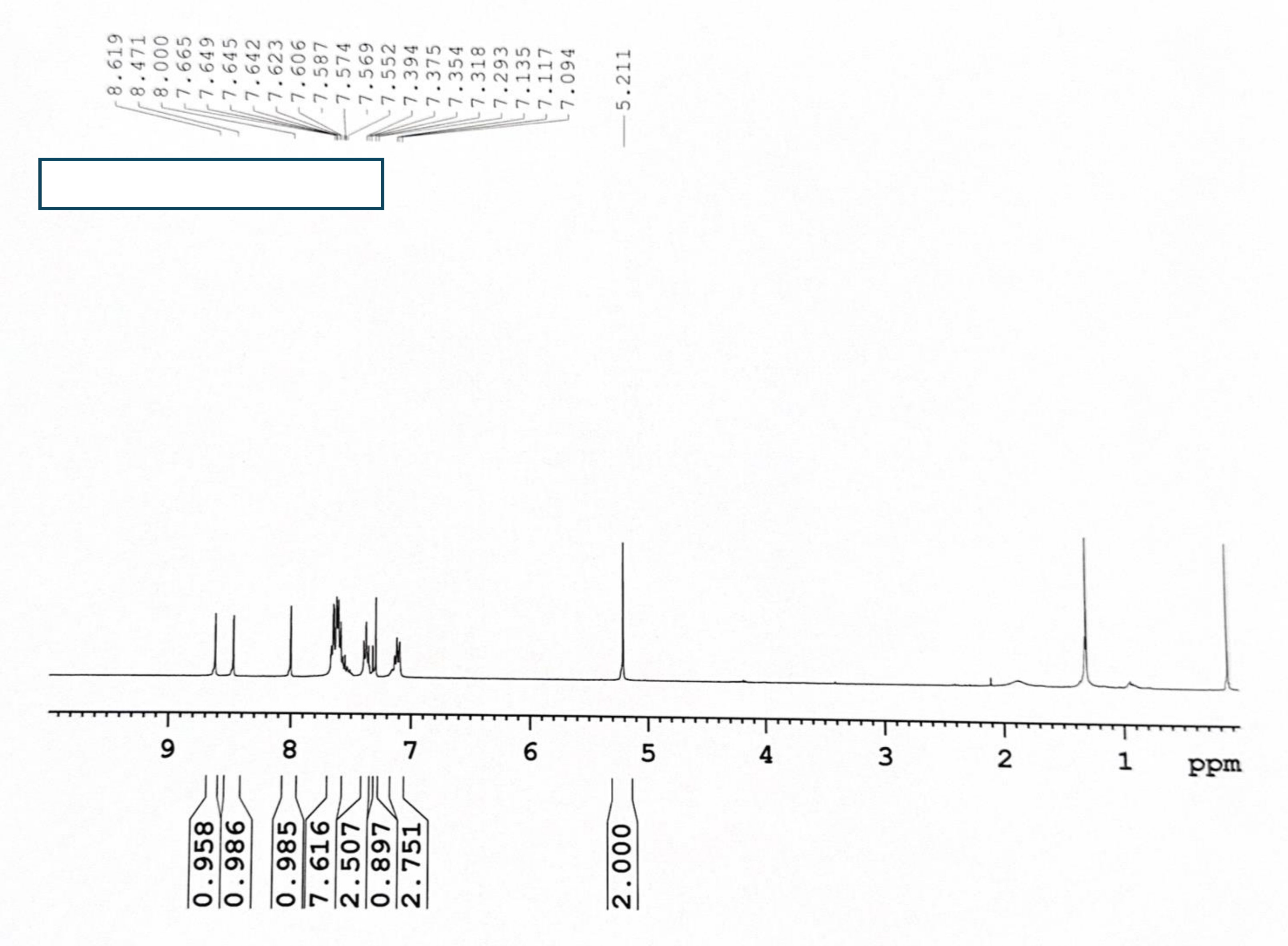
8.619
8.471
8.000
7.665
7.649
7.645
7.642
7.623
7.606
7.587
7.574
7.569
7.552
7.394
7.375
7.354
7.318
7.293
7.135
7.117
7.094
5.211
9
8
7
6
5
4
3
2
1
ppm
0.958
0.986
0.985
7.616
2.507
0.897
2.751
2.000

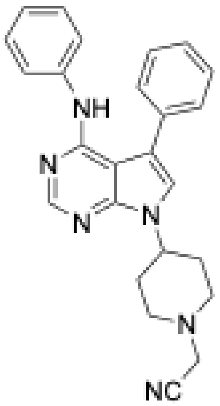
NH
N
N
N
N
NC

12a

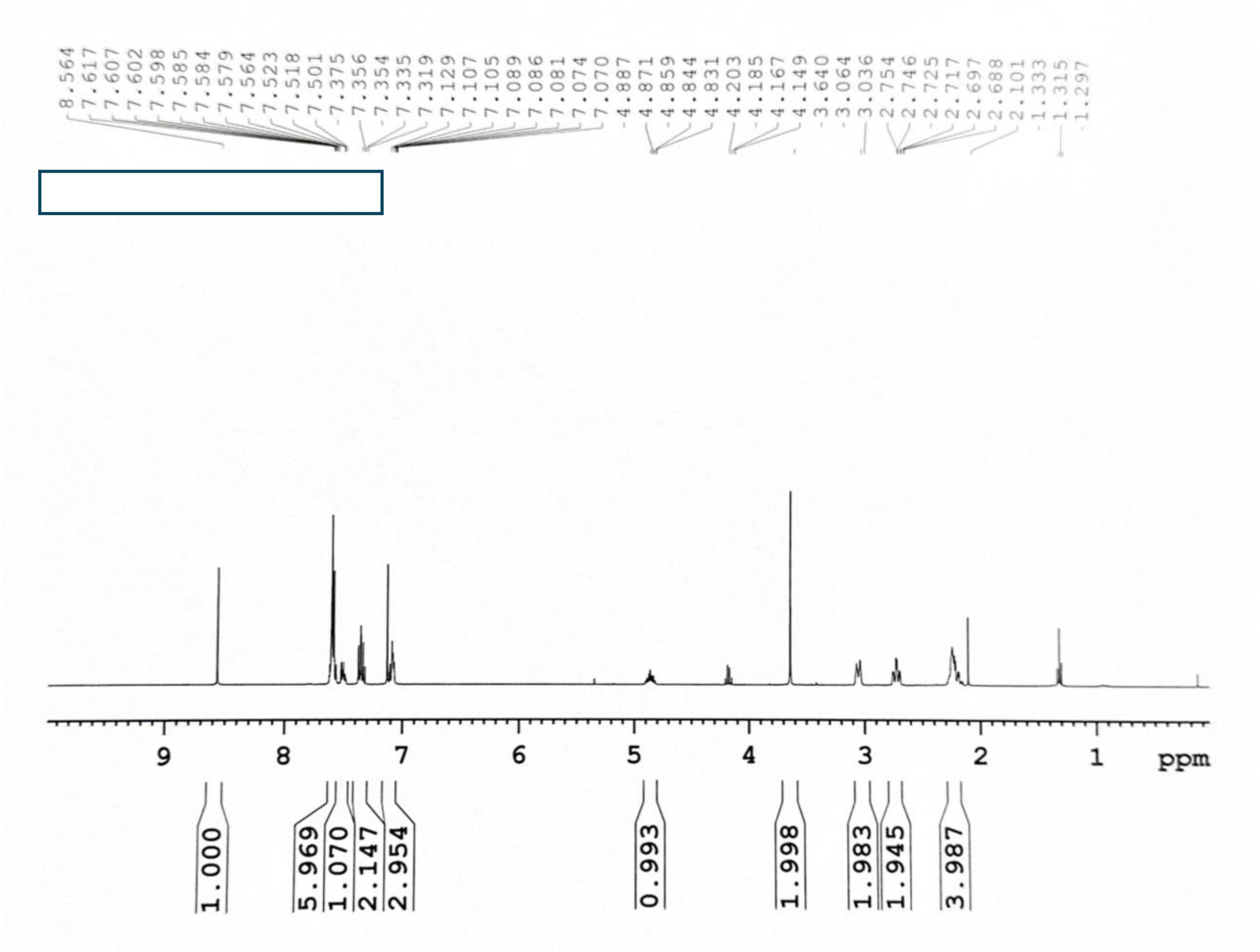
8.564
7.617
7.607
7.602
7.598
7.585
7.584
7.579
7.564
7.523
7.518
7.501
7.375
7.356
7.354
7.335
7.319
7.129
7.107
7.105
7.089
7.086
7.081
7.074
7.070
4.887
4.871
4.859
4.844
4.831
4.203
4.185
4.167
4.149
3.640
3.064
3.036
2.754
2.746
2.725
2.717
2.697
2.688
2.101
1.333
1.315
1.297
9
8
7
6
5
4
3
2
1
ppm
1.000
5.969
1.070
2.147
2.954
0.993
1.998
1.983
1.945
3.987

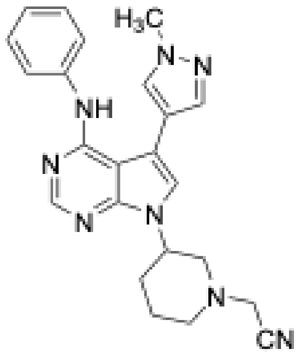
H3C
N
N
NH
N
N
N
N
CN

12b

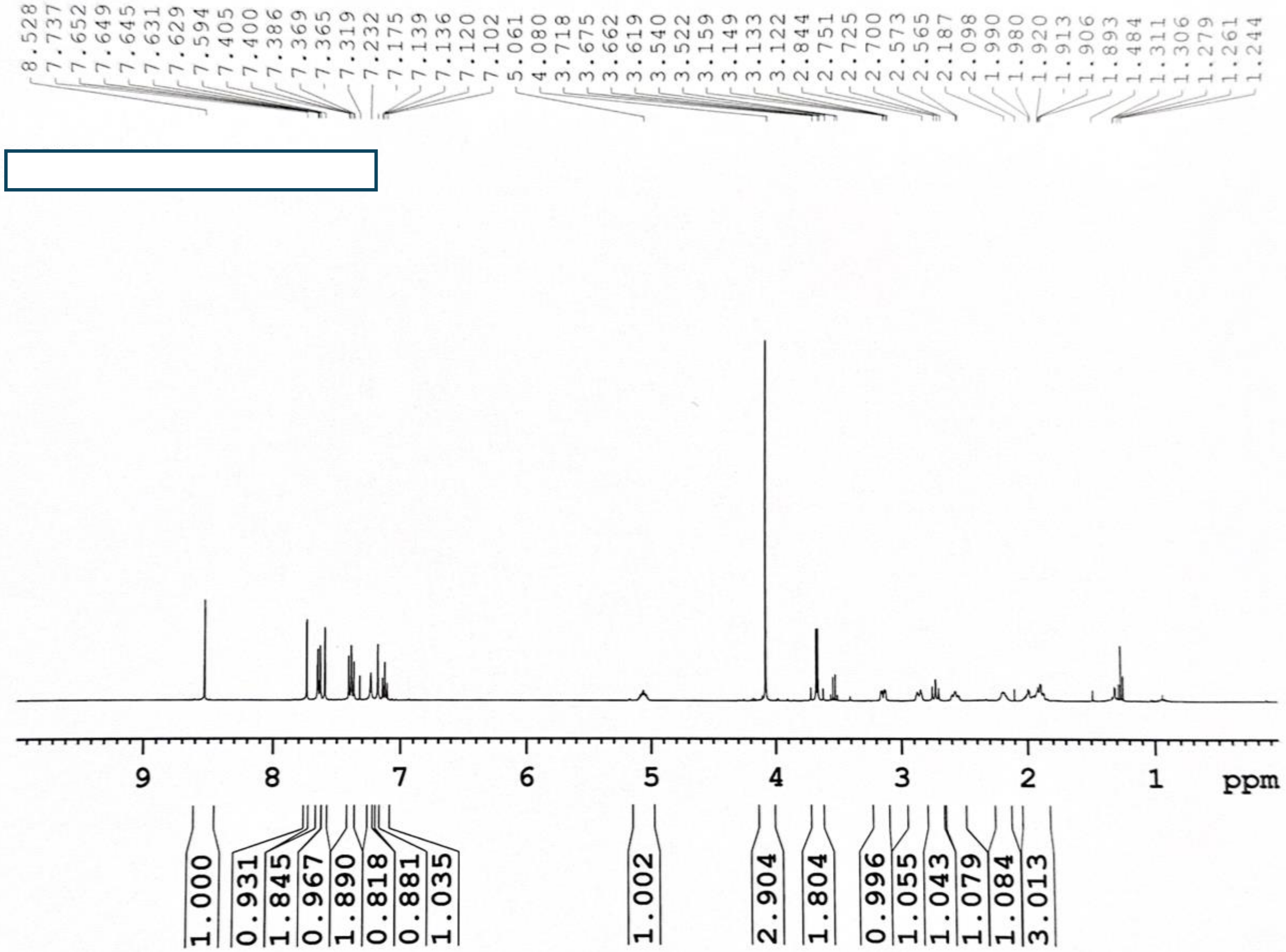
8.528
7.737
7.652
7.649
7.645
7.631
7.629
7.594
7.405
7.400
7.386
7.369
7.365
7.319
7.232
7.175
7.139
7.136
7.120
7.102
5.061
4.080
3.718
3.675
3.662
3.619
3.540
3.522
3.159
3.149
3.133
3.122
2.844
2.751
2.725
2.700
2.573
2.565
2.187
2.098
1.990
1.980
1.920
1.913
1.906
1.893
1.484
1.311
1.306
1.279
1.261
1.244
9
8
7
6
5
4
3
2
1
ppm
1.000
0.931
1.845
0.967
1.890
0.818
0.881
1.035
1.002
2.904
1.804
0.996
1.055
1.043
1.079
1.084
3.013

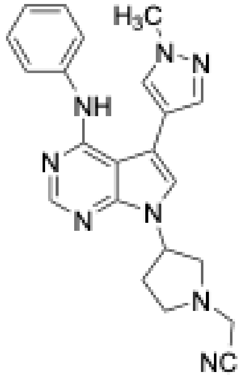

H3C
N
N
NH
N
N
N
N
NC

12c

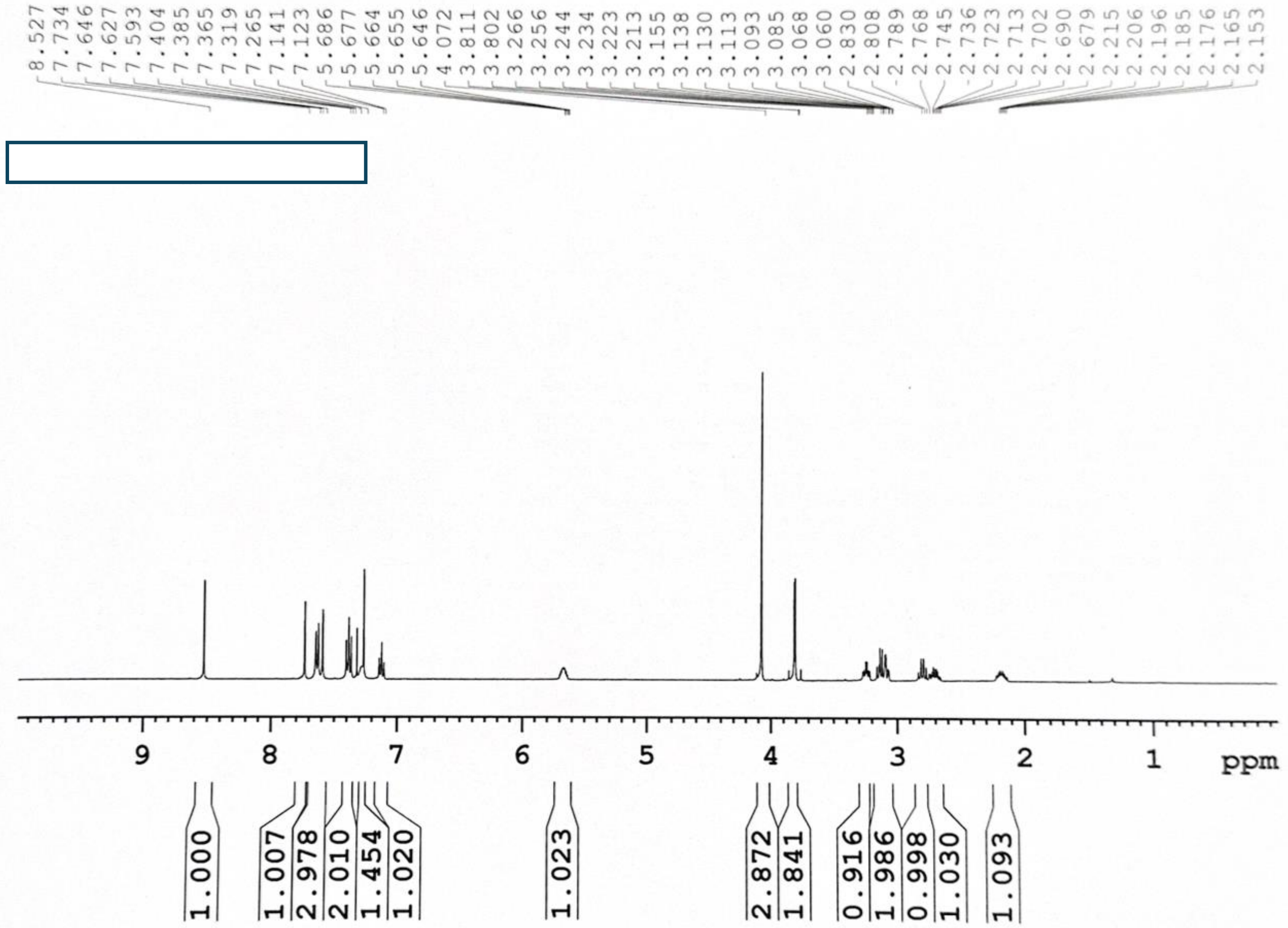

8.527
7.734
7.646
7.627
7.593
7.404
7.385
7.365
7.319
7.265
7.141
7.123
5.686
5.677
5.664
5.655
5.646
4.072
3.811
3.802
3.266
3.256
3.244
3.234
3.223
3.213
3.155
3.138
3.130
3.113
3.093
3.085
3.068
3.060
2.830
2.808
2.789
2.768
2.745
2.736
2.723
2.713
2.702
2.690
2.679
2.215
2.206
2.196
2.185
2.176
2.165
2.153
9
8
7
6
5
4
3
2
1
ppm
1.000
1.007
2.978
2.010
1.454
1.020
1.023
2.872
1.841
0.916
1.986
0.998
1.030
1.093

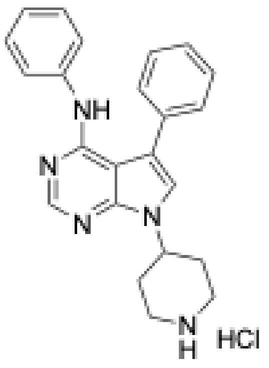
NH
N
N
N
N
H
HCl

13

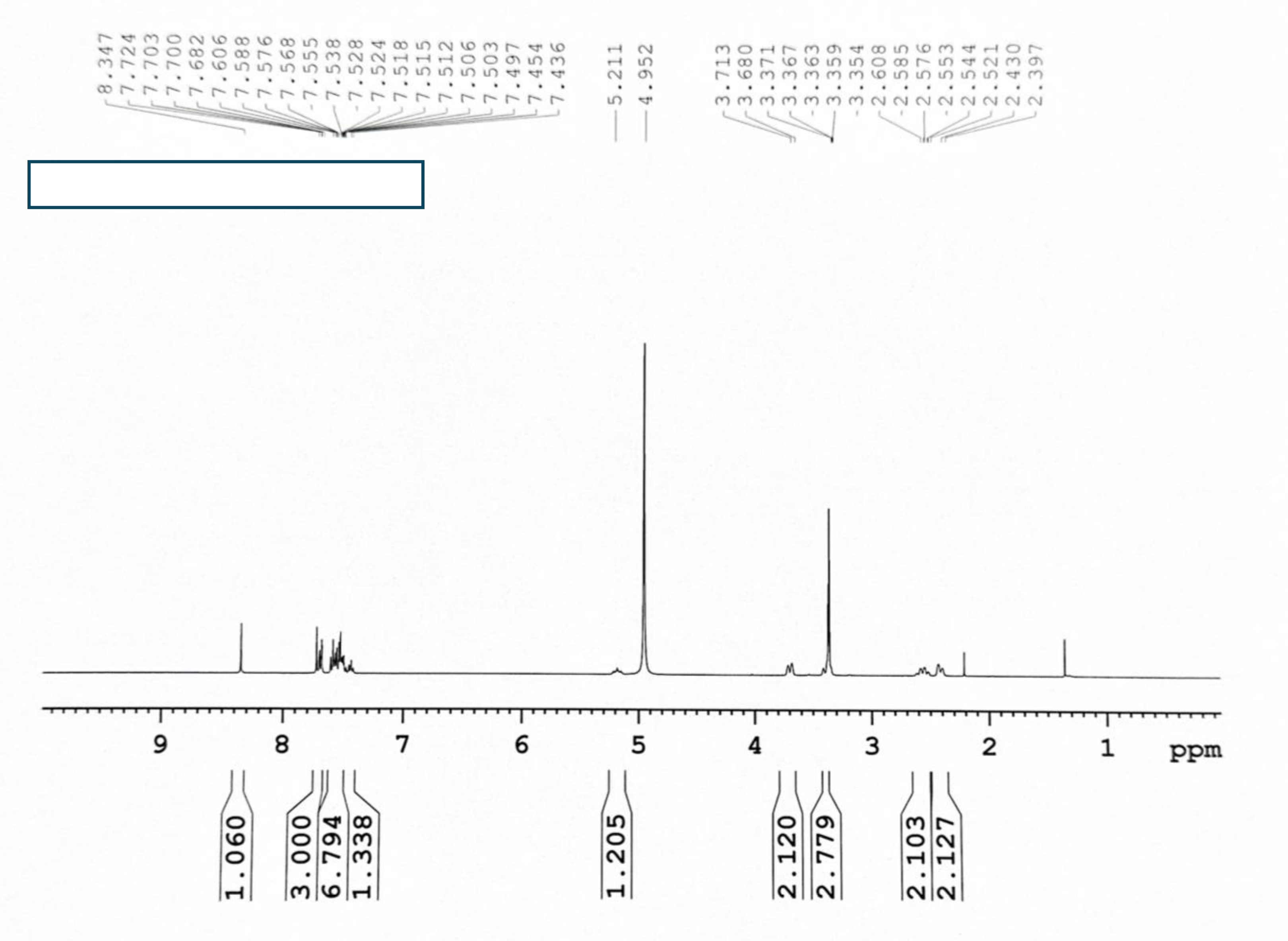
8.347
7.724
7.703
7.700
7.682
7.606
7.588
7.576
7.568
7.555
7.538
7.528
7.524
7.518
7.515
7.512
7.506
7.503
7.497
7.454
7.436
5.211
4.952
3.713
3.680
3.371
3.367
3.363
3.359
3.354
2.608
2.585
2.576
2.553
2.544
2.521
2.430
2.397
9
8
7
6
5
4
3
2
1
ppm
1.060
3.000
6.794
1.338
1.205
2.120
2.779
2.103
2.127

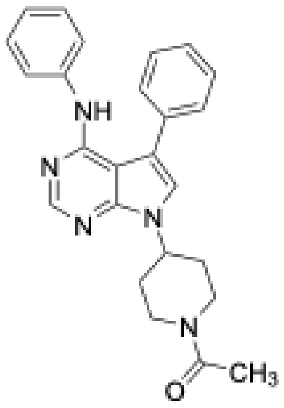
NH
N
N
N
N
O
CH3

14a

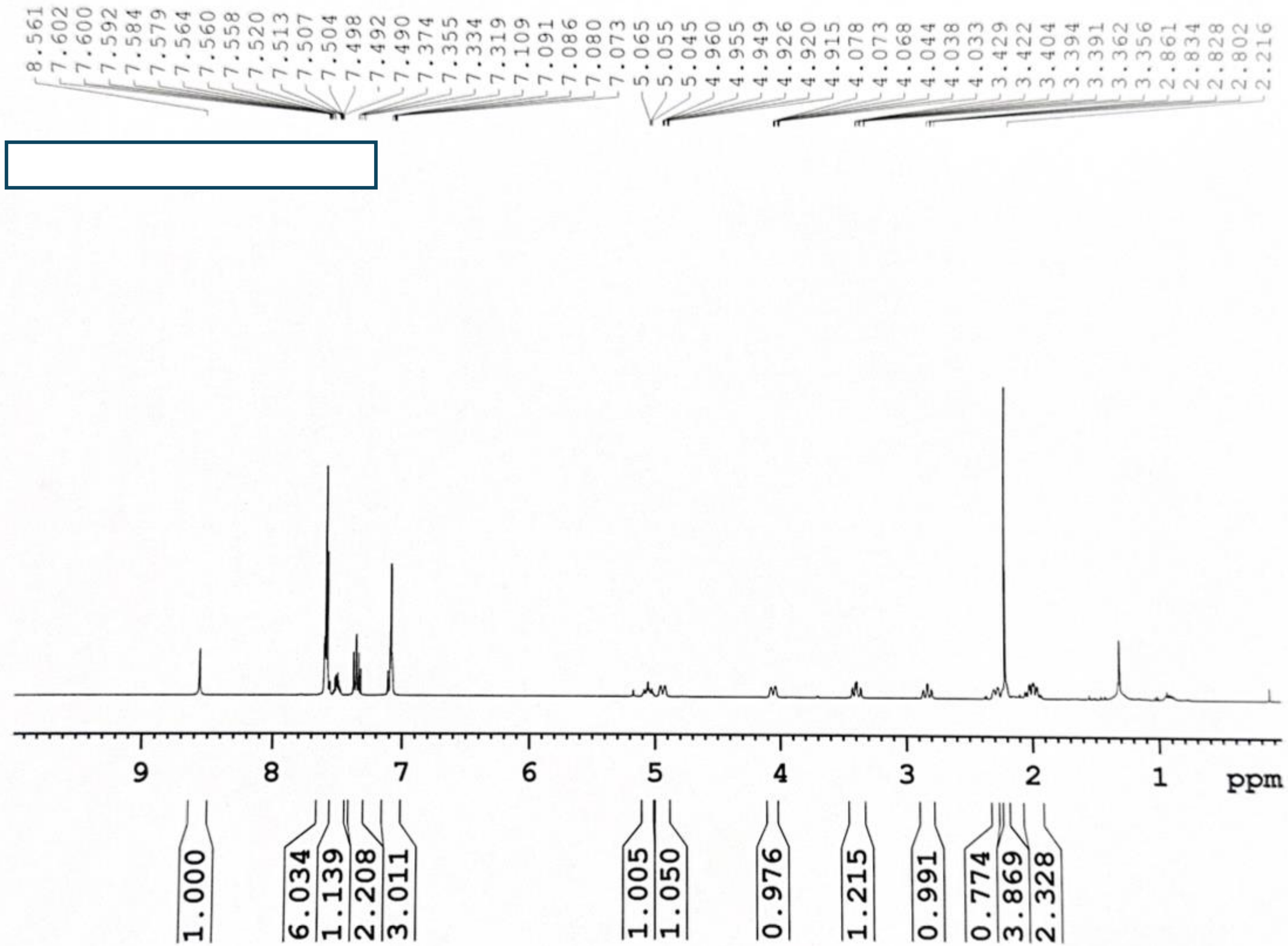
8.561
7.602
7.600
7.592
7.584
7.579
7.564
7.560
7.558
7.520
7.513
7.507
7.504
7.498
7.492
7.490
7.374
7.355
7.334
7.319
7.109
7.091
7.086
7.080
7.073
5.065
5.055
5.045
4.960
4.955
4.949
4.926
4.920
4.915
4.078
4.073
4.068
4.044
4.038
4.033
3.429
3.422
3.404
3.394
3.391
3.362
3.356
2.861
2.834
2.828
2.802
2.216
9
8
7
6
5
4
3
2
1
ppm
1.000
6.034
1.139
2.208
3.011
1.005
1.050
0.976
1.215
0.991
0.774
3.869
2.328

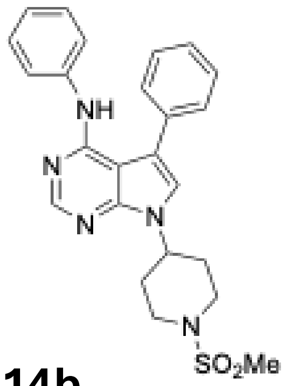
NH
N
N
N
N
SO2Me

14b

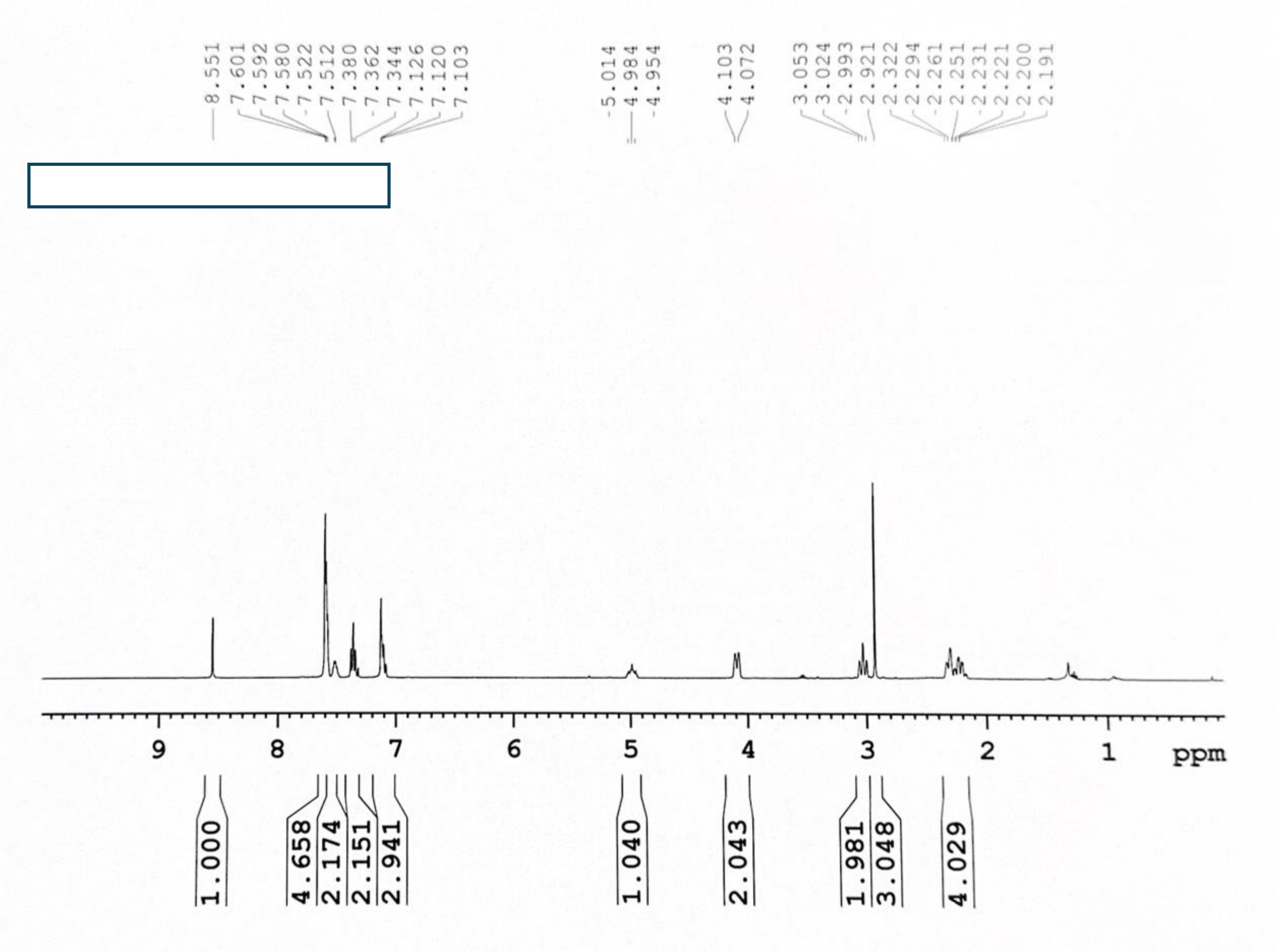
8.551
7.601
7.592
7.580
7.522
7.512
7.380
7.362
7.344
7.126
7.120
7.103
5.014
4.984
4.954
4.103
4.072
3.053
3.024
2.993
2.921
2.322
2.294
2.261
2.251
2.231
2.221
2.200
2.191
9
8
7
6
5
4
3
2
1
ppm
1.000
4.658
2.174
2.151
2.941
1.040
2.043
1.981
3.048
4.029

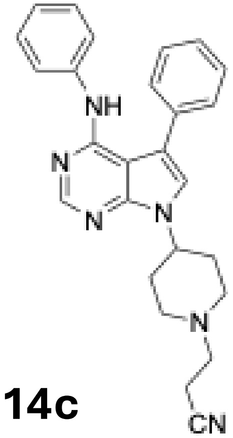

14c

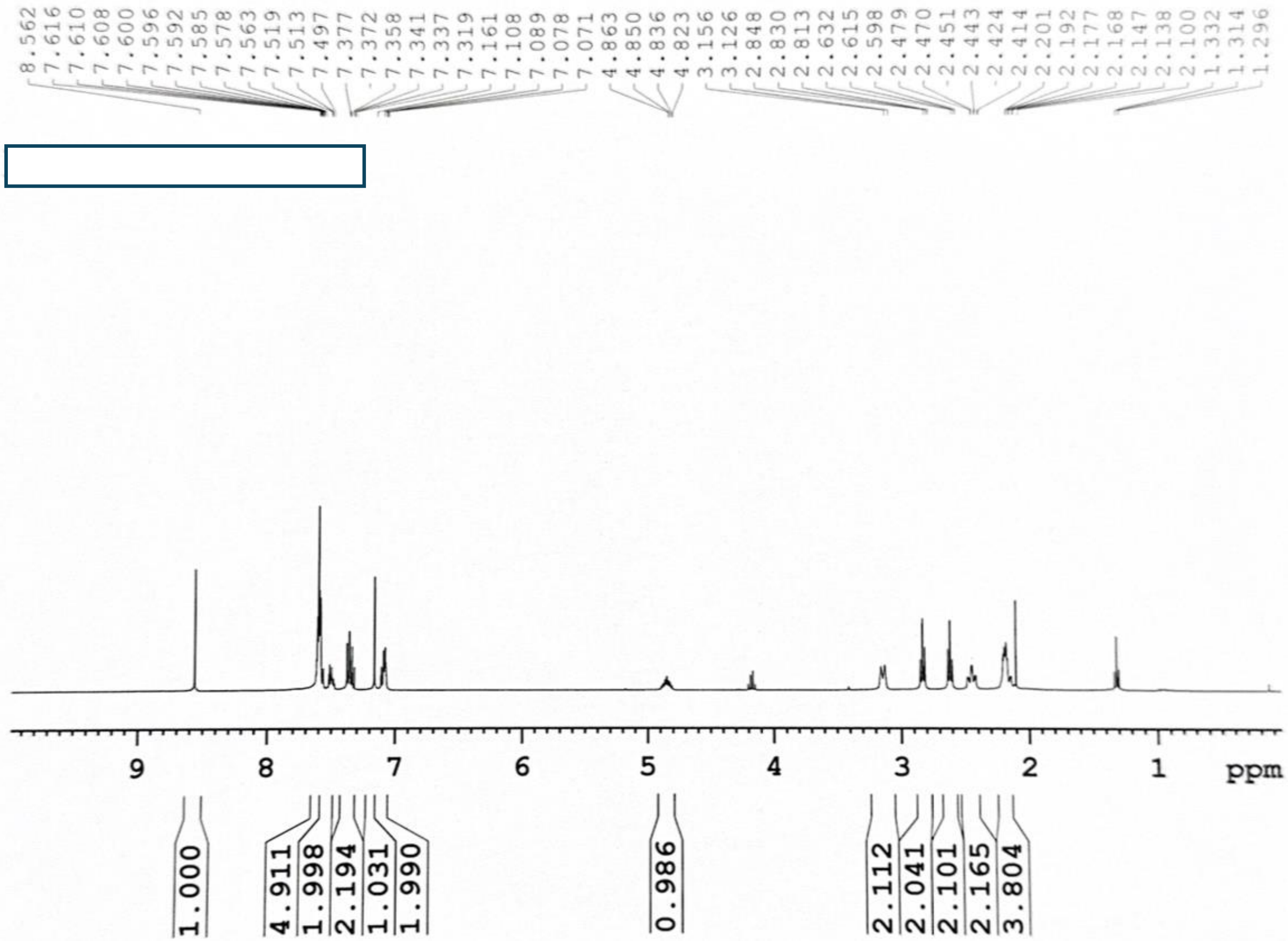
8.562
7.616
7.610
7.608
7.600
7.596
7.592
7.585
7.578
7.563
7.519
7.513
7.497
7.377
7.372
7.358
7.341
7.337
7.319
7.161
7.108
7.089
7.078
7.071
4.863
4.850
4.836
4.823
3.156
3.126
2.848
2.830
2.813
2.632
2.615
2.598
2.479
2.470
2.451
2.443
2.424
2.414
2.201
2.192
2.177
2.168
2.147
2.138
2.100
1.332
1.314
1.296
9
8
7
6
5
4
3
2
1
ppm
1.000
4.911
1.998
2.194
1.031
1.990
0.986
2.112
2.041
2.101
2.165
3.804

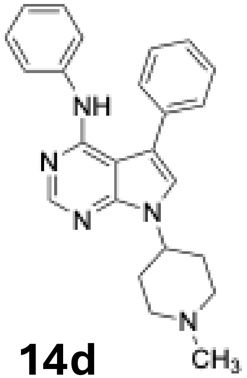

14d

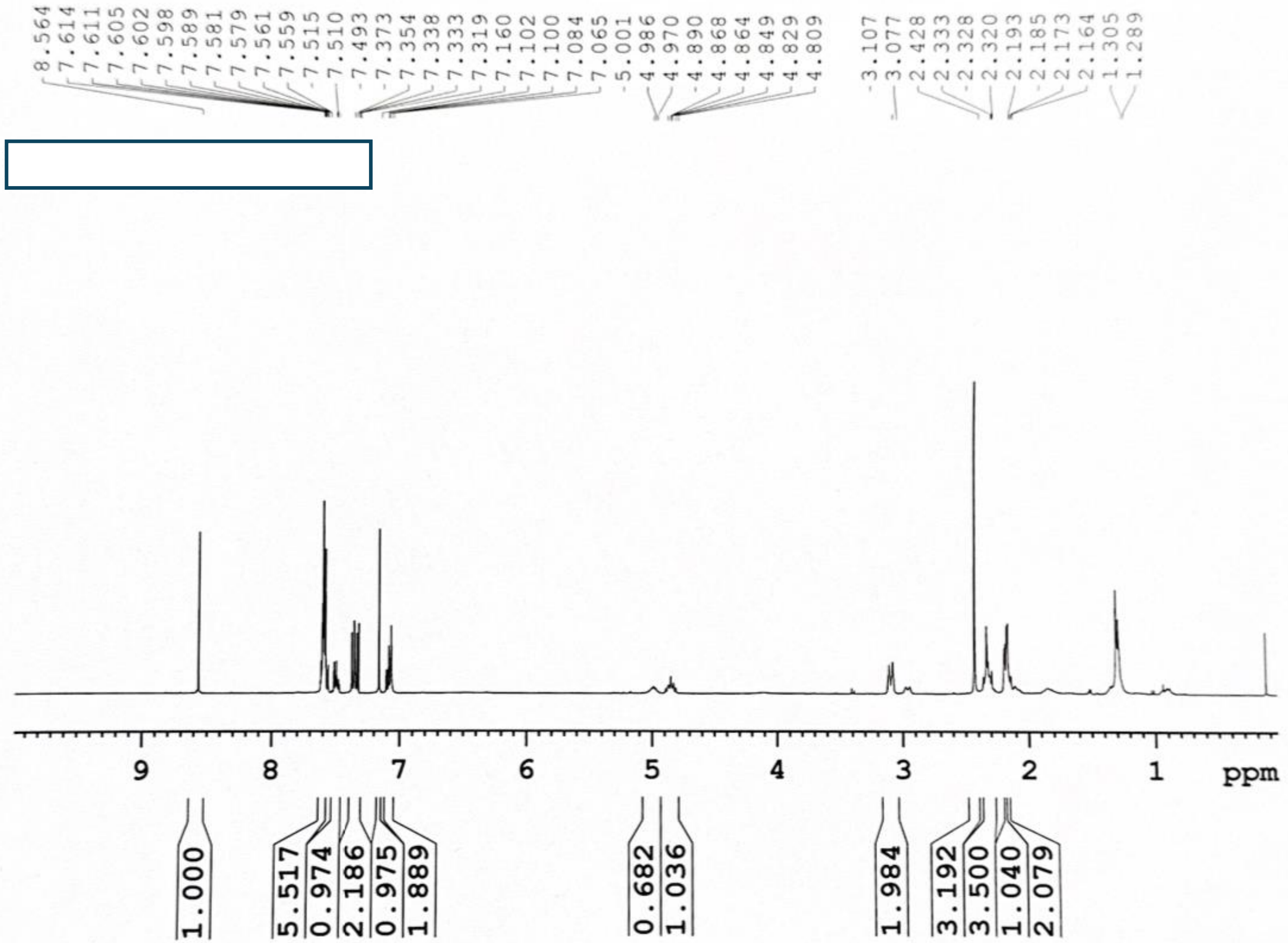
8.564
7.614
7.611
7.605
7.602
7.598
7.589
7.581
7.579
7.561
7.559
7.515
7.510
7.493
7.373
7.354
7.338
7.333
7.319
7.160
7.102
7.100
7.084
7.065
5.001
4.986
4.970
4.890
4.868
4.864
4.849
4.829
4.809
3.107
3.077
2.428
2.333
2.328
2.320
2.193
2.185
2.173
2.164
1.305
1.289
9
8
7
6
5
4
3
2
1
ppm
1.000
5.517
0.974
2.186
0.975
1.889
0.682
1.036
1.984
3.192
3.500
1.040
2.079

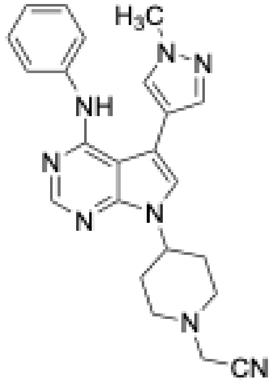
H3C
N
N
NH
N
N
N
N
CN

15a

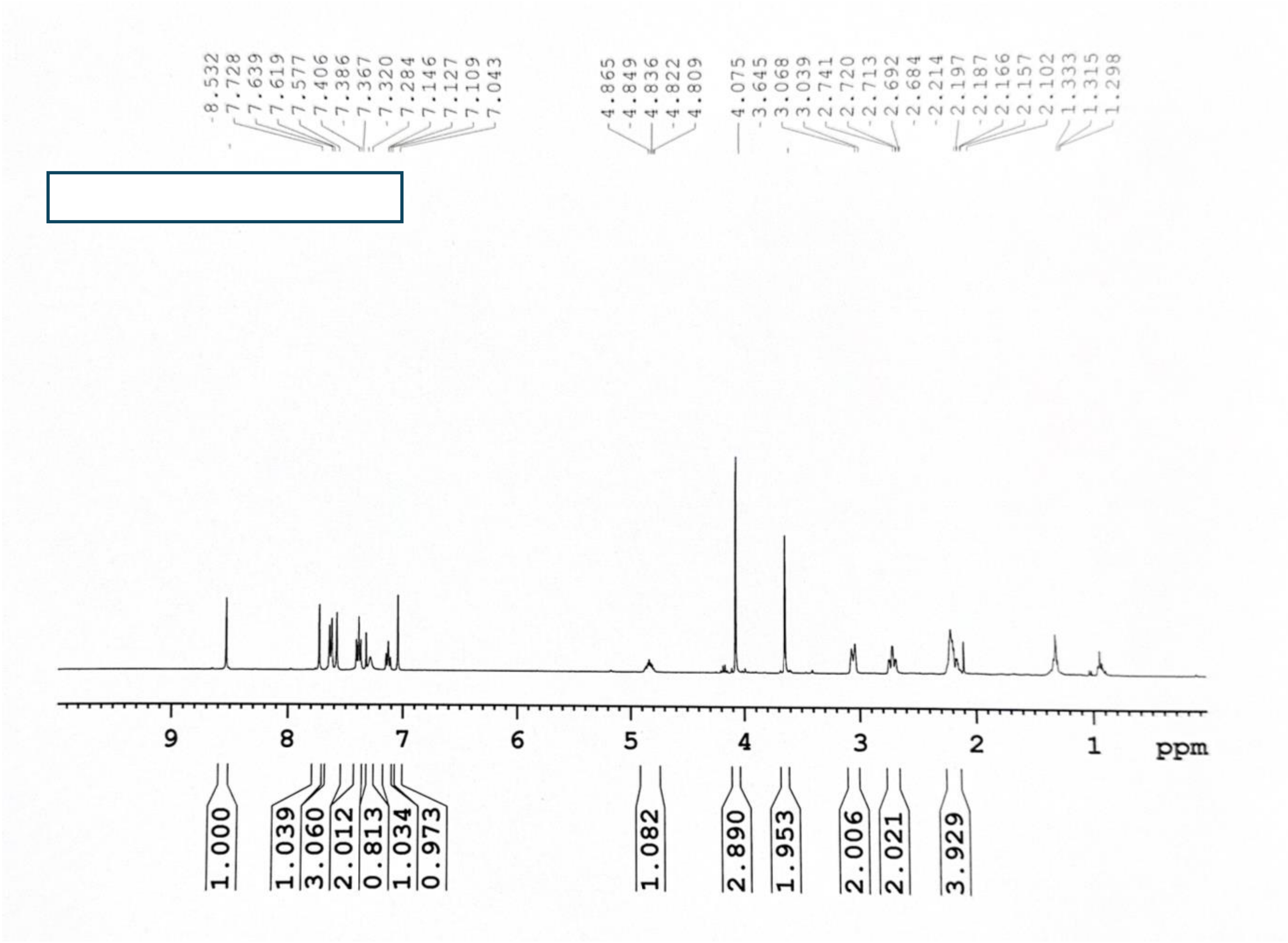
8.532
7.728
7.639
7.619
7.577
7.406
7.386
7.367
7.320
7.284
7.146
7.127
7.109
7.043
4.865
4.849
4.836
4.822
4.809
4.075
3.645
3.068
3.039
2.741
2.720
2.713
2.692
2.684
2.214
2.197
2.187
2.166
2.157
2.102
1.333
1.315
1.298
9
8
7
6
5
4
3
2
1
ppm
1.000
1.039
3.060
2.012
0.813
1.034
0.973
1.082
2.890
1.953
2.006
2.021
3.929

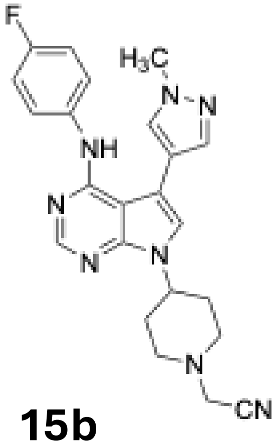
F
H_3C
N
N
NH
N
N
N
N
CN

15b

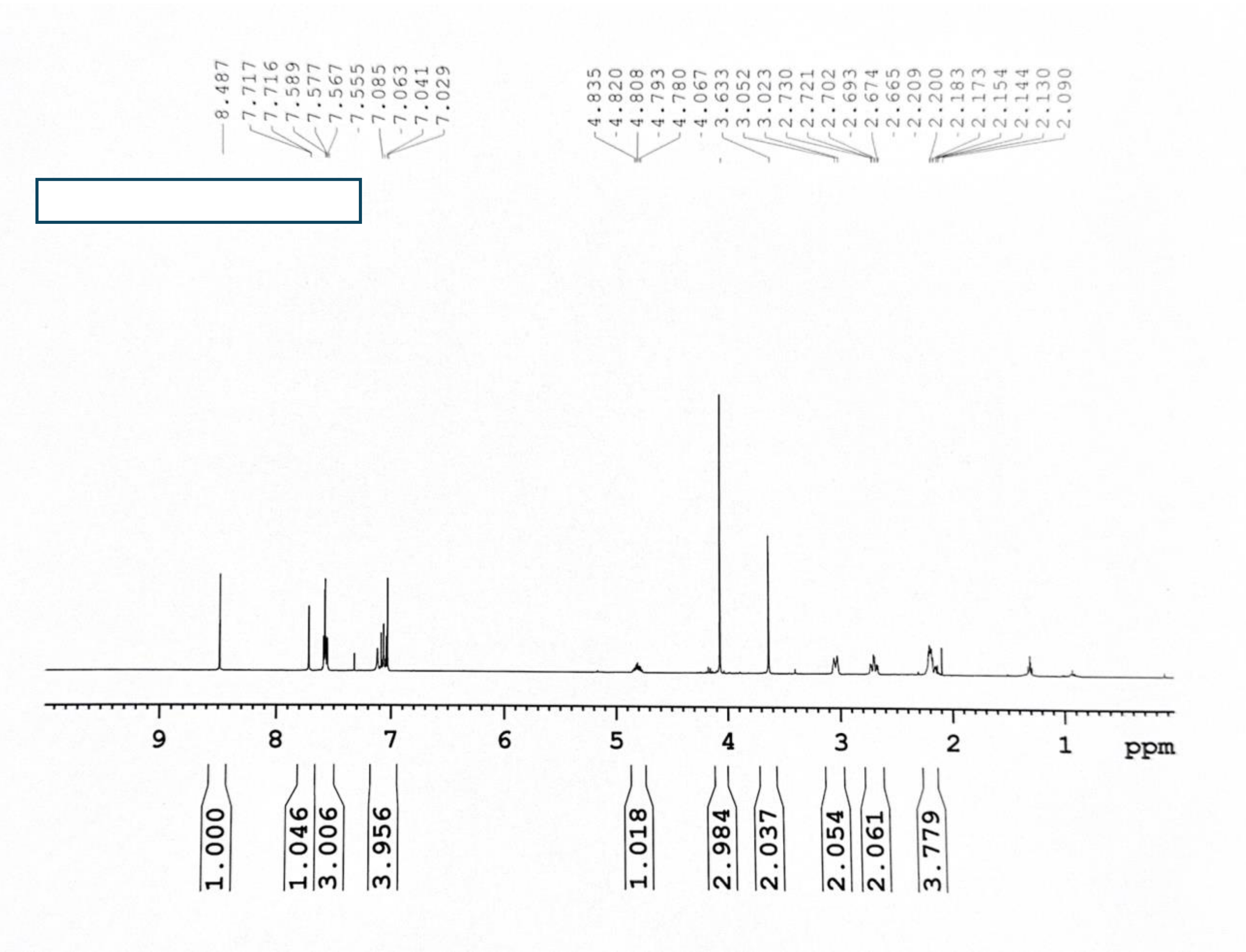
8.487
7.717
7.716
7.589
7.577
7.567
7.555
7.085
7.063
7.041
7.029
4.835
4.820
4.808
4.793
4.780
4.067
3.633
3.052
3.023
2.730
2.721
2.702
2.693
2.674
2.665
2.209
2.200
2.183
2.173
2.154
2.144
2.130
2.090
9
8
7
6
5
4
3
2
1
ppm
1.000
1.046
3.006
3.956
1.018
2.984
2.037
2.054
2.061
3.779

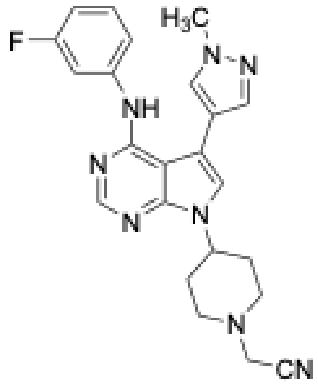

15c

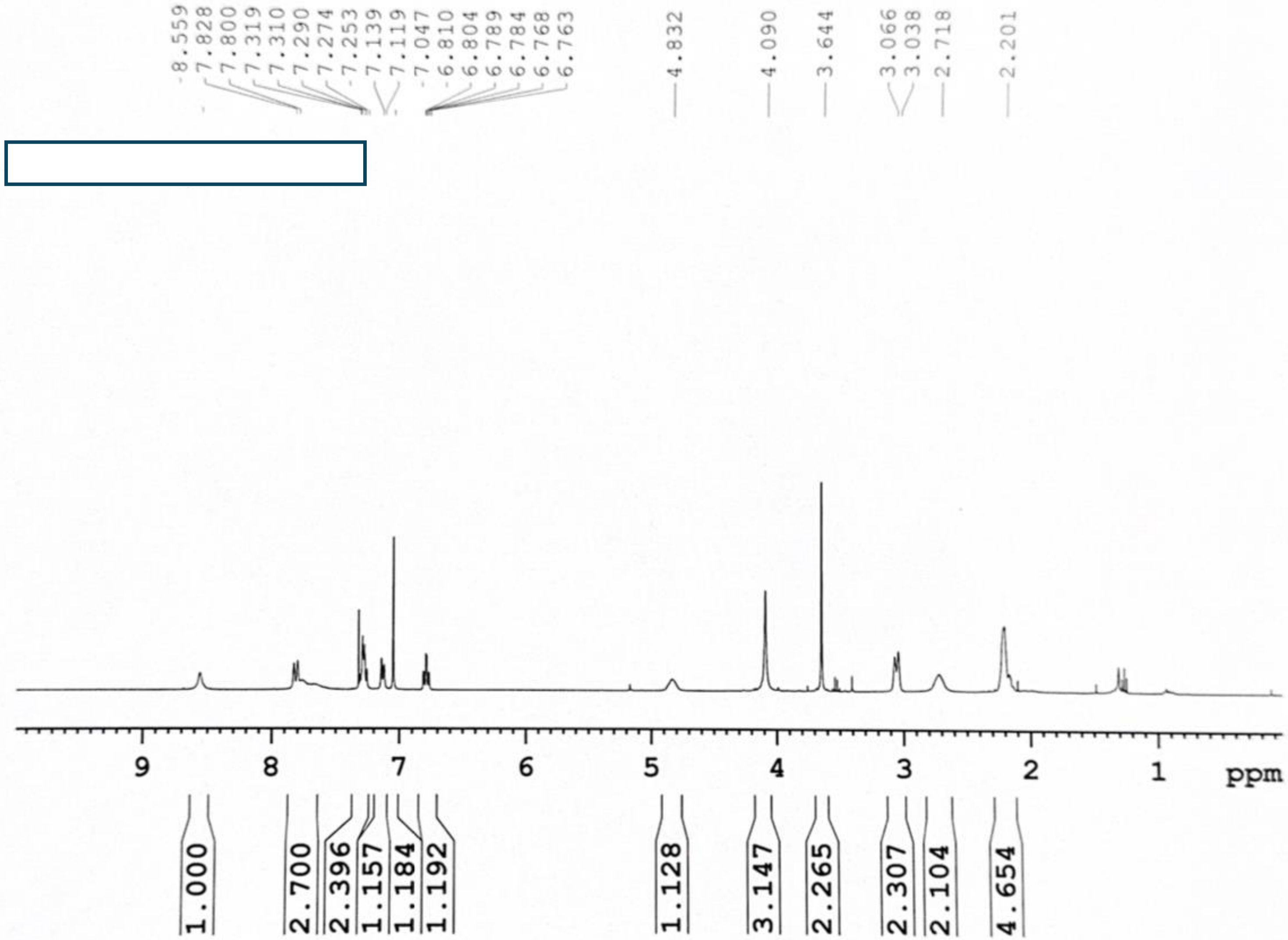
8.559
7.828
7.800
7.319
7.310
7.290
7.274
7.253
7.139
7.119
7.047
6.810
6.804
6.789
6.784
6.768
6.763
4.832
4.090
3.644
3.066
3.038
2.718
2.201
9
8
7
6
5
4
3
2
1
ppm
1.000
2.700
2.396
1.157
1.184
1.192
1.128
3.147
2.265
2.307
2.104
4.654

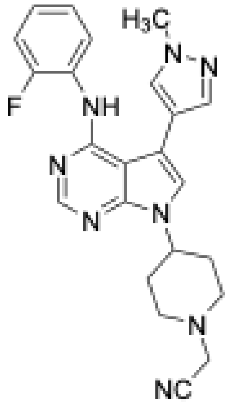
H3C
N
N
F
NH
N
N
N
N
NC

15d

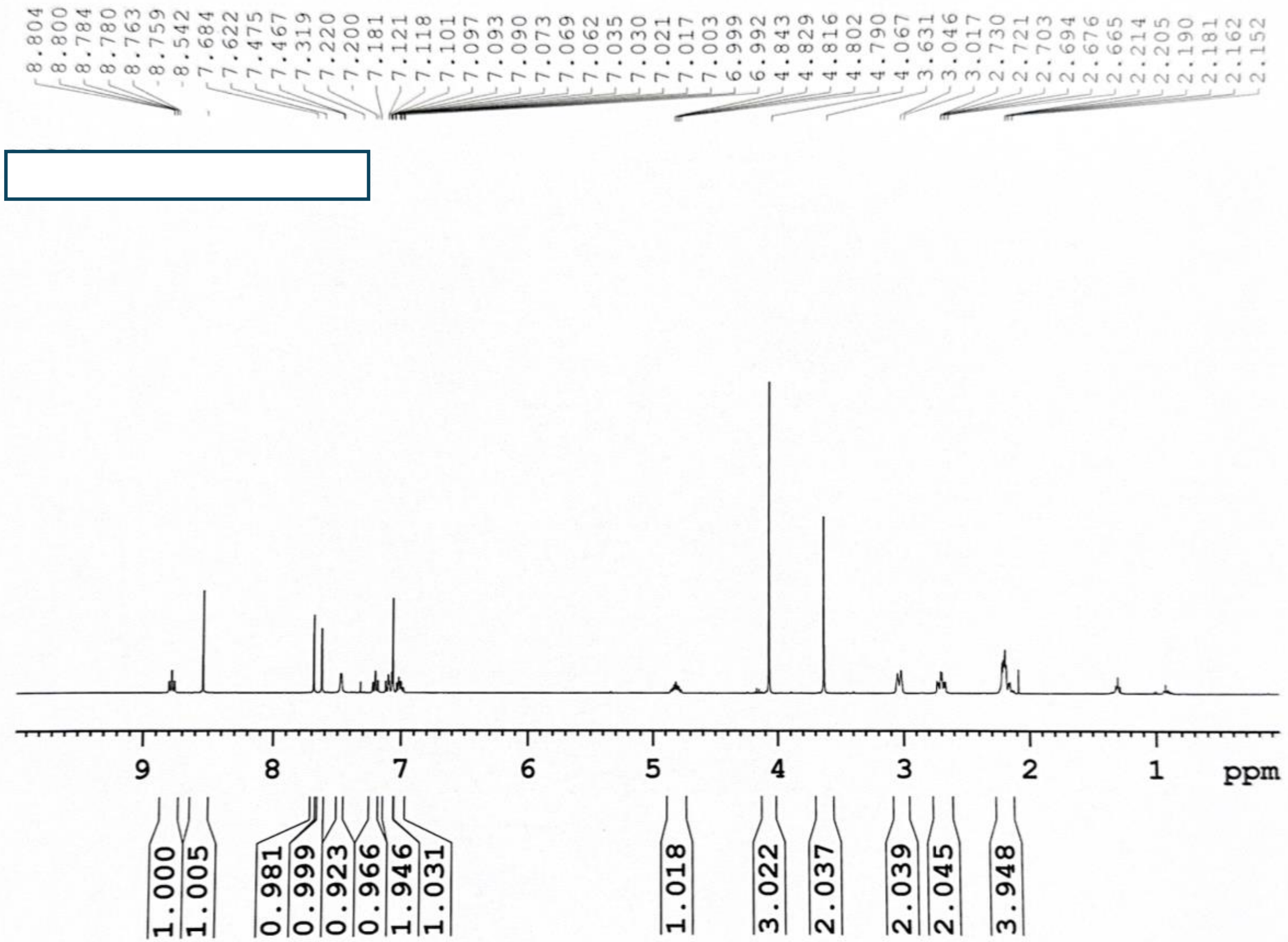
8.804
8.800
8.784
8.780
8.763
8.759
8.542
7.684
7.622
7.475
7.467
7.319
7.220
7.200
7.181
7.121
7.118
7.101
7.097
7.093
7.090
7.073
7.069
7.062
7.035
7.030
7.021
7.017
7.003
6.999
6.992
4.843
4.829
4.816
4.802
4.790
4.067
3.631
3.046
3.017
2.730
2.721
2.703
2.694
2.676
2.665
2.214
2.205
2.190
2.181
2.162
2.152
9
8
7
6
5
4
3
2
1
ppm
1.000
1.005
0.981
0.999
0.923
0.966
1.946
1.031
1.018
3.022
2.037
2.039
2.045
3.948

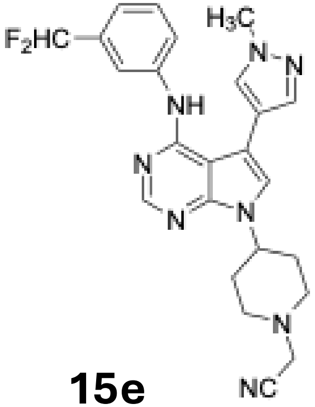
F2HC
H3C
N
NH
NC

15e

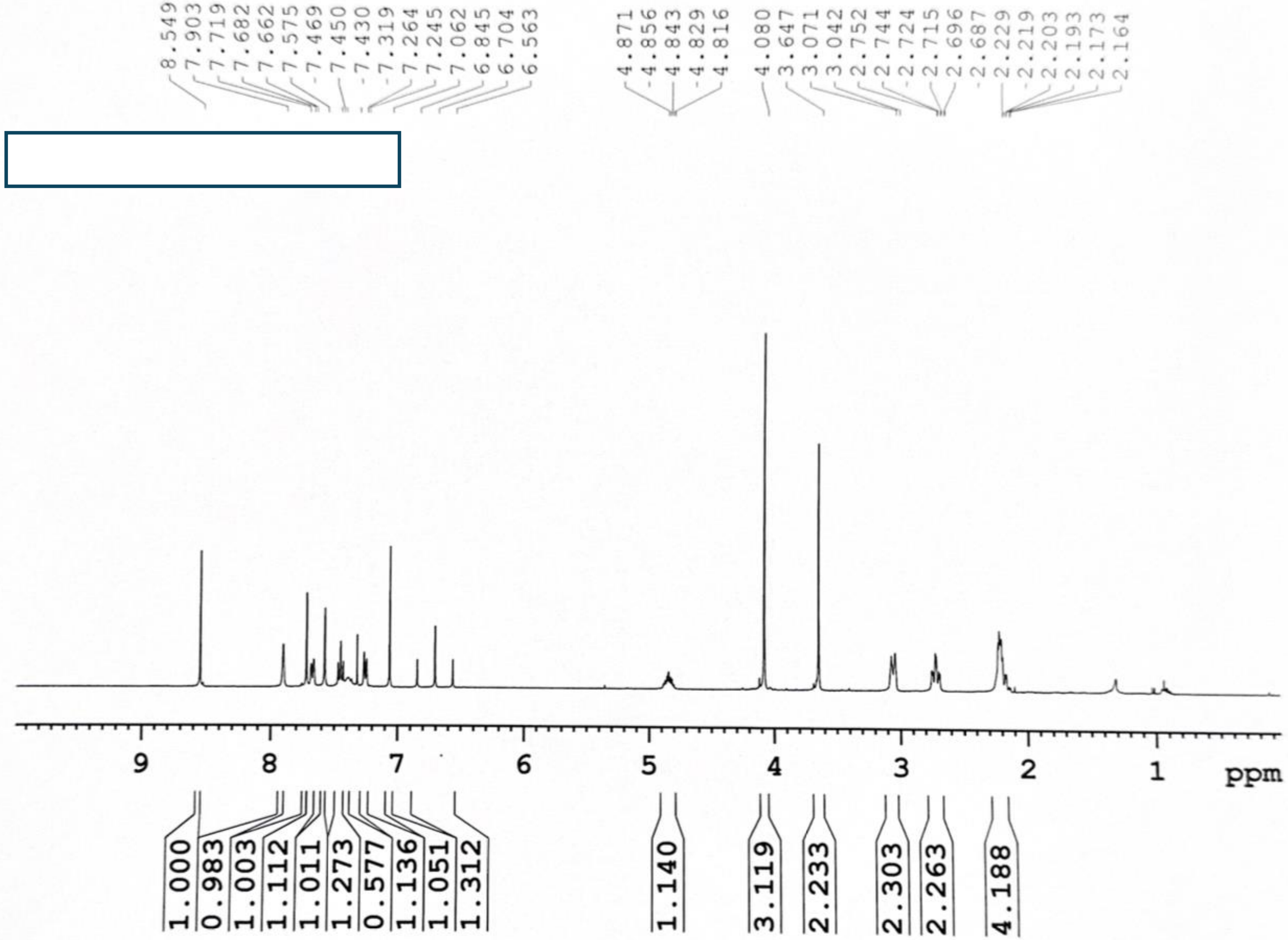
8.549
7.903
7.719
7.682
7.662
7.575
7.469
7.450
7.430
7.319
7.264
7.245
7.062
6.845
6.704
6.563
4.871
4.856
4.843
4.829
4.816
4.080
3.647
3.071
3.042
2.752
2.744
2.724
2.715
2.696
2.687
2.229
2.219
2.203
2.193
2.173
2.164
9
8
7
6
5
4
3
2
1
ppm
1.000
0.983
1.003
1.112
1.011
1.273
0.577
1.136
1.051
1.312
1.140
3.119
2.233
2.303
2.263
4.188

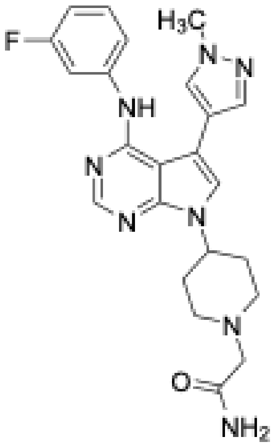
F
NH
H3C
N
N
N
N
N
N
O
NH2

15f

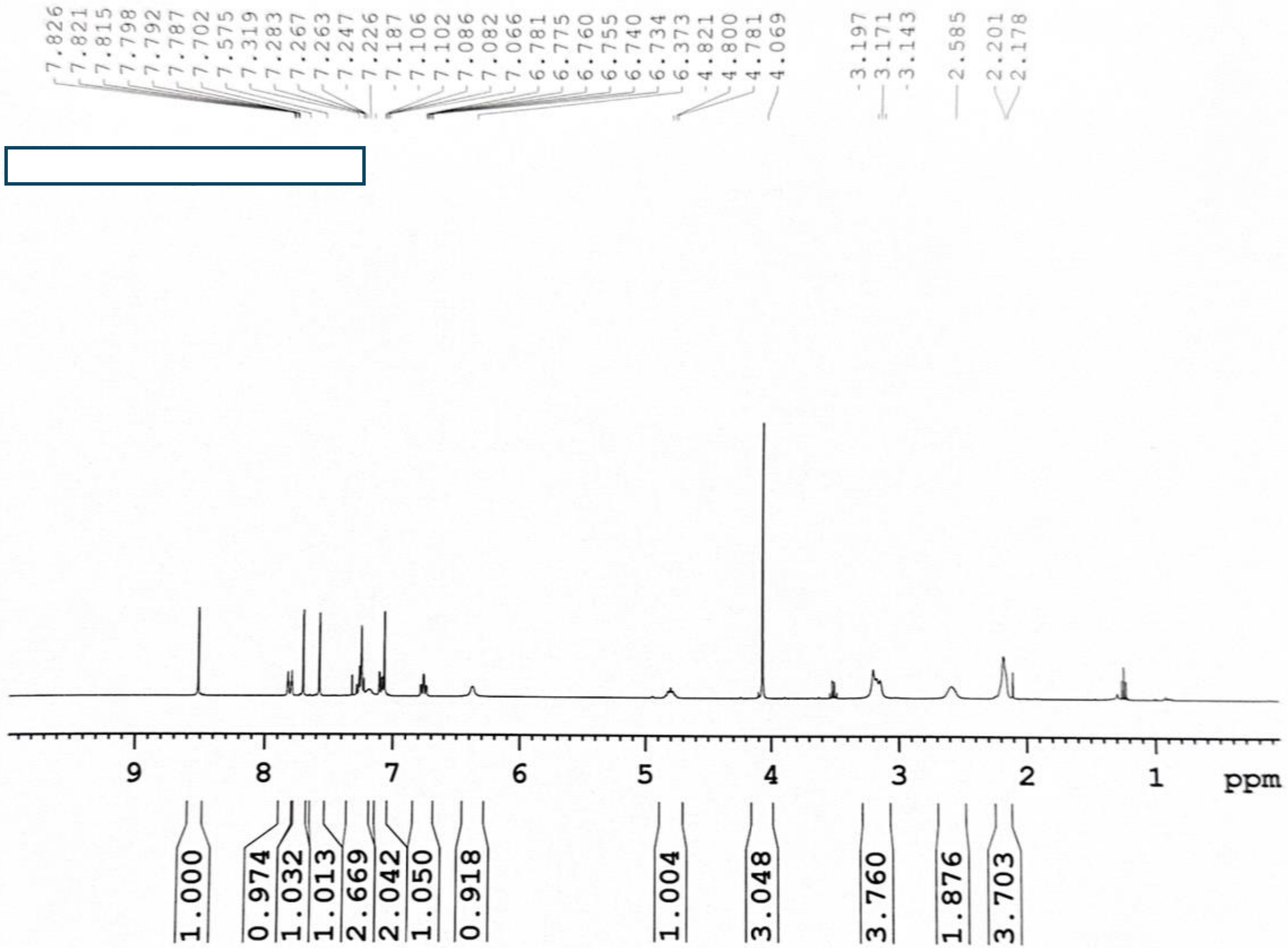
7.826
7.821
7.815
7.798
7.792
7.787
7.702
7.575
7.319
7.283
7.267
7.263
7.247
7.226
7.187
7.106
7.102
7.086
7.082
7.066
6.781
6.775
6.760
6.755
6.740
6.734
6.373
4.821
4.800
4.781
4.069
3.197
3.171
3.143
2.585
2.201
2.178
9
8
7
6
5
4
3
2
1
ppm
1.000
0.974
1.032
1.013
2.669
2.042
1.050
0.918
1.004
3.048
3.760
1.876
3.703

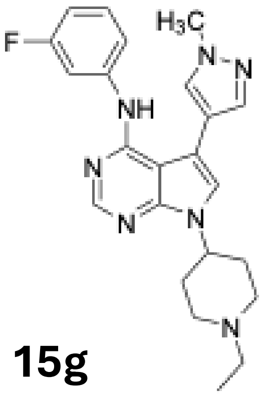
F
H3C
N
N
NH
N
N
N
N

15g

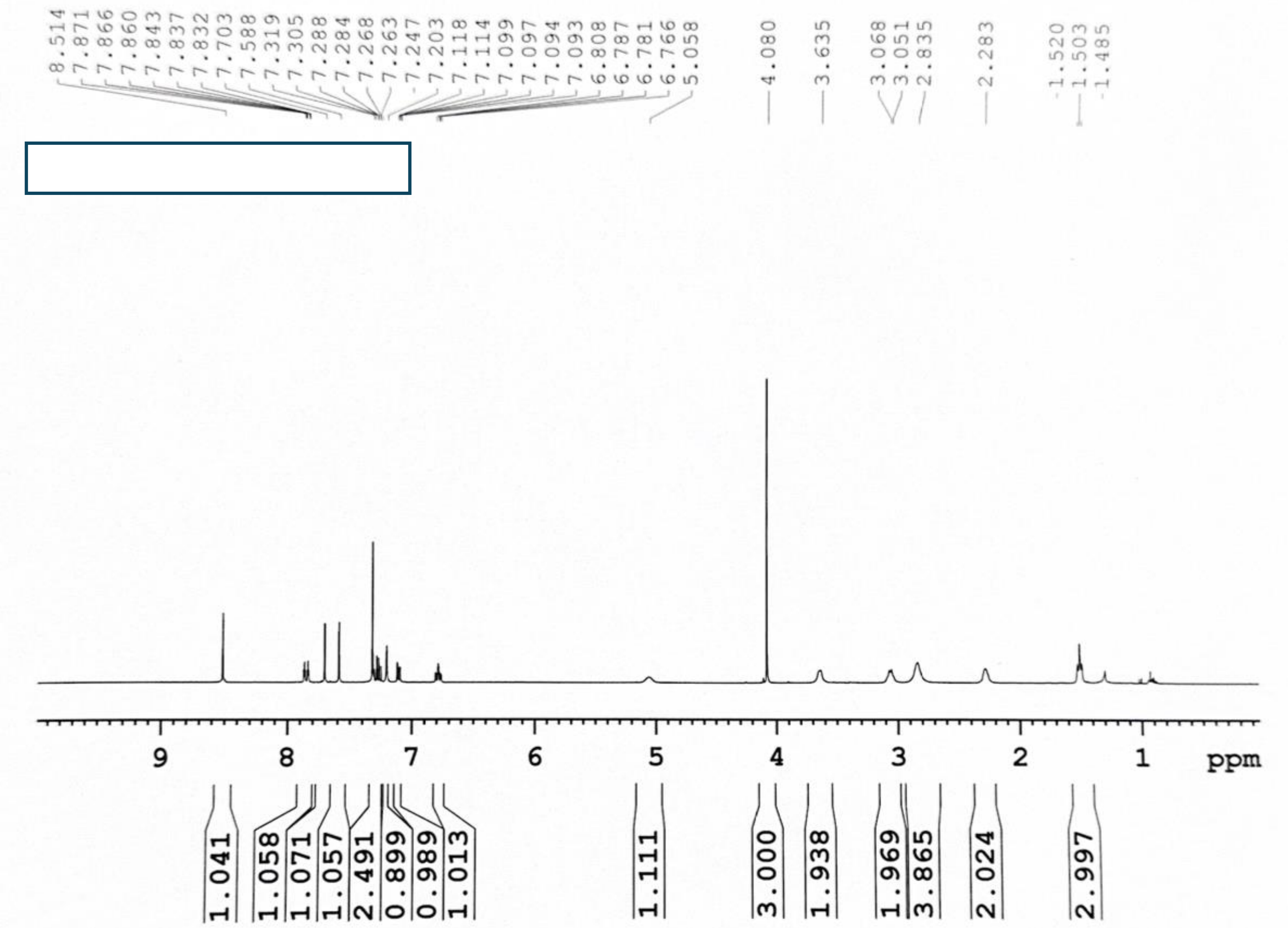
8.514
7.871
7.866
7.860
7.843
7.837
7.832
7.703
7.588
7.319
7.305
7.288
7.284
7.268
7.263
7.247
7.203
7.118
7.114
7.099
7.097
7.094
7.093
6.808
6.787
6.781
6.766
5.058
4.080
3.635
3.068
3.051
2.835
2.283
1.520
1.503
1.485
9
8
7
6
5
4
3
2
1
ppm
1.041
1.058
1.071
1.057
2.491
0.899
0.989
1.013
1.111
3.000
1.938
1.969
3.865
2.024
2.997

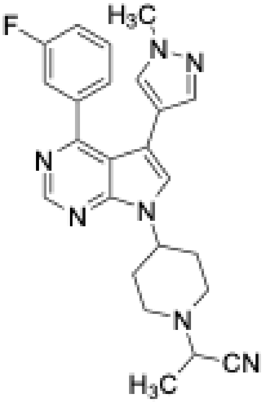
F
H_3C
N
N
N
N
N
N
CN
H_3C

15h

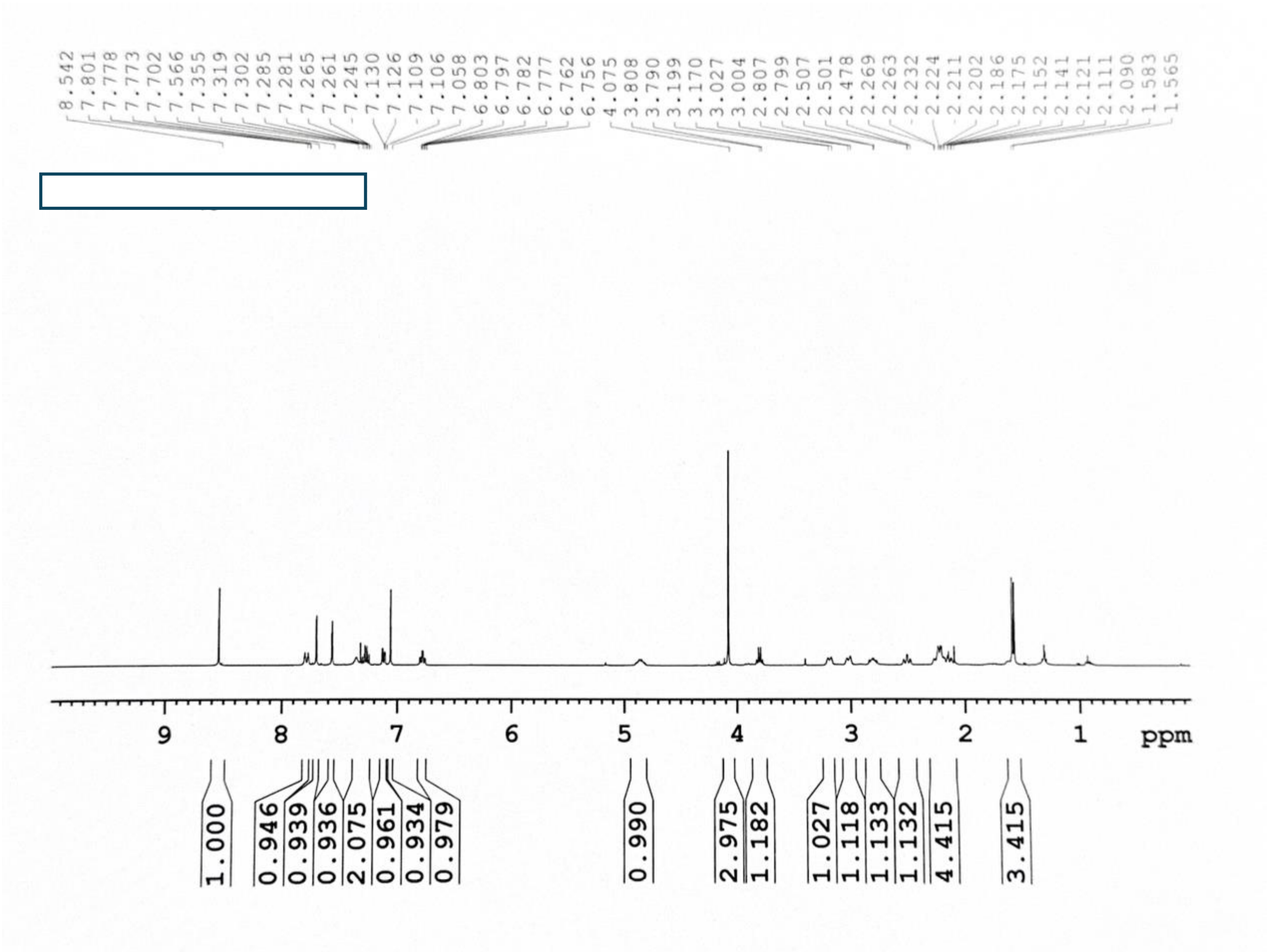
8.542
7.801
7.778
7.773
7.702
7.566
7.355
7.319
7.302
7.285
7.281
7.265
7.261
7.245
7.130
7.126
7.109
7.106
7.058
6.803
6.797
6.782
6.777
6.762
6.756
4.075
3.808
3.790
3.199
3.170
3.027
3.004
2.807
2.799
2.507
2.501
2.478
2.269
2.263
2.232
2.224
2.211
2.202
2.186
2.175
2.152
2.141
2.121
2.111
2.090
1.583
1.565
9
8
7
6
5
4
3
2
1
ppm
1.000
0.946
0.939
0.936
2.075
0.961
0.934
0.979
0.990
2.975
1.182
1.027
1.118
1.133
1.132
4.415
3.415

^{1}H NMR (400 MHz, $CD_3OD+CDCl_3$)

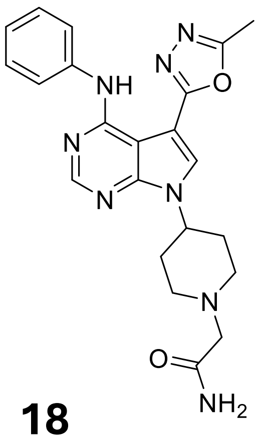

18

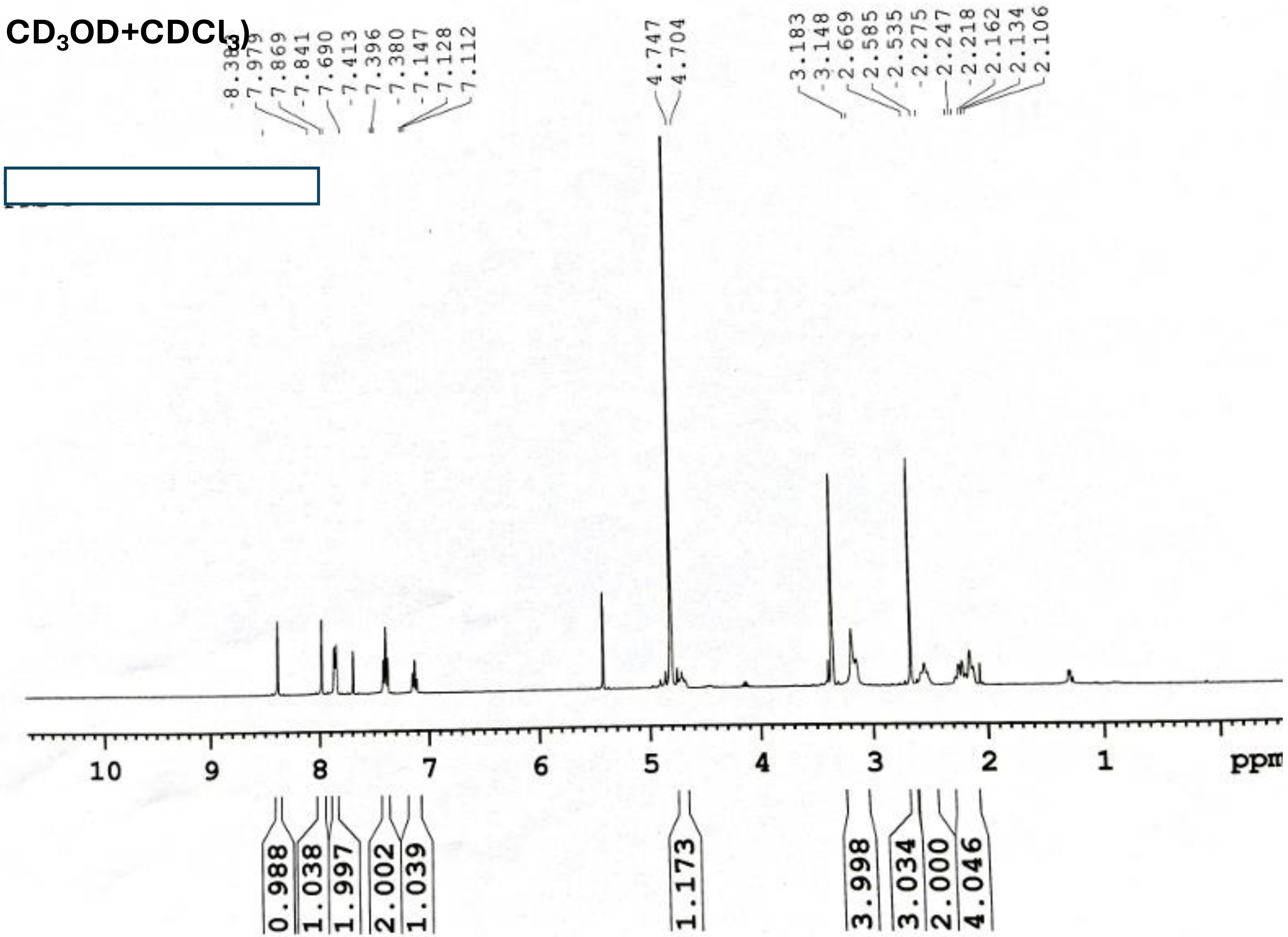

^1H NMR (400 MHz, CD_3OD)

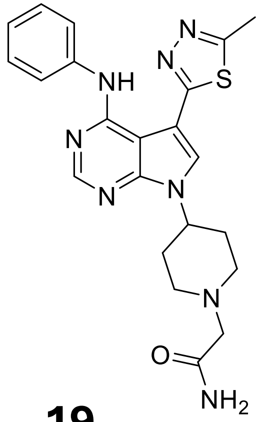

19

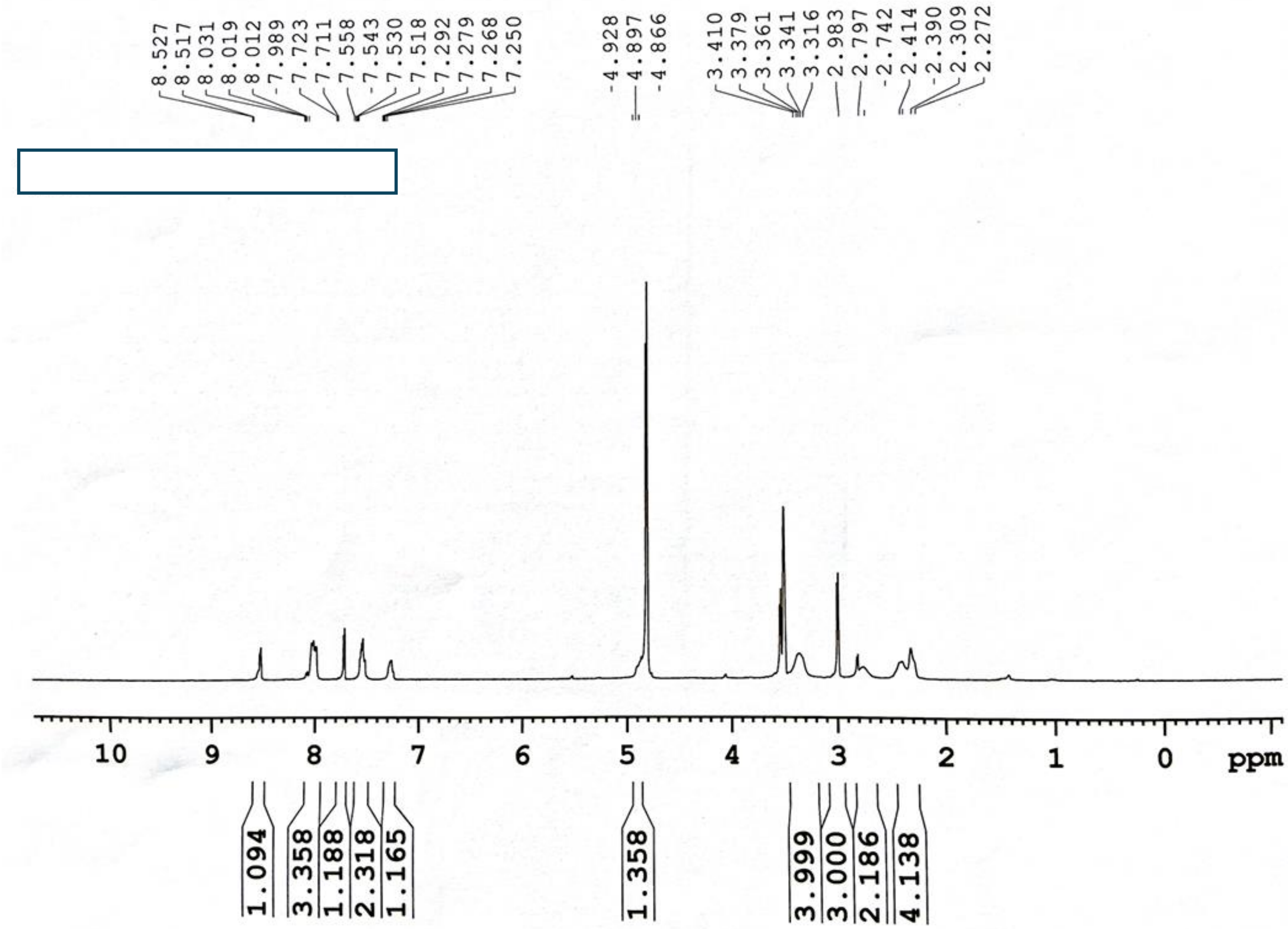

^1H NMR (400 MHz, $CD_3OD+CDCl_3$)

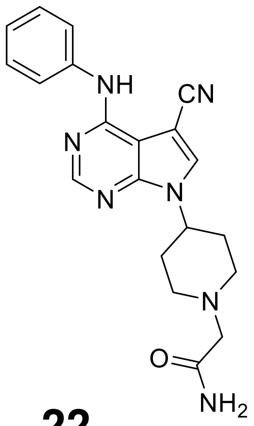

22

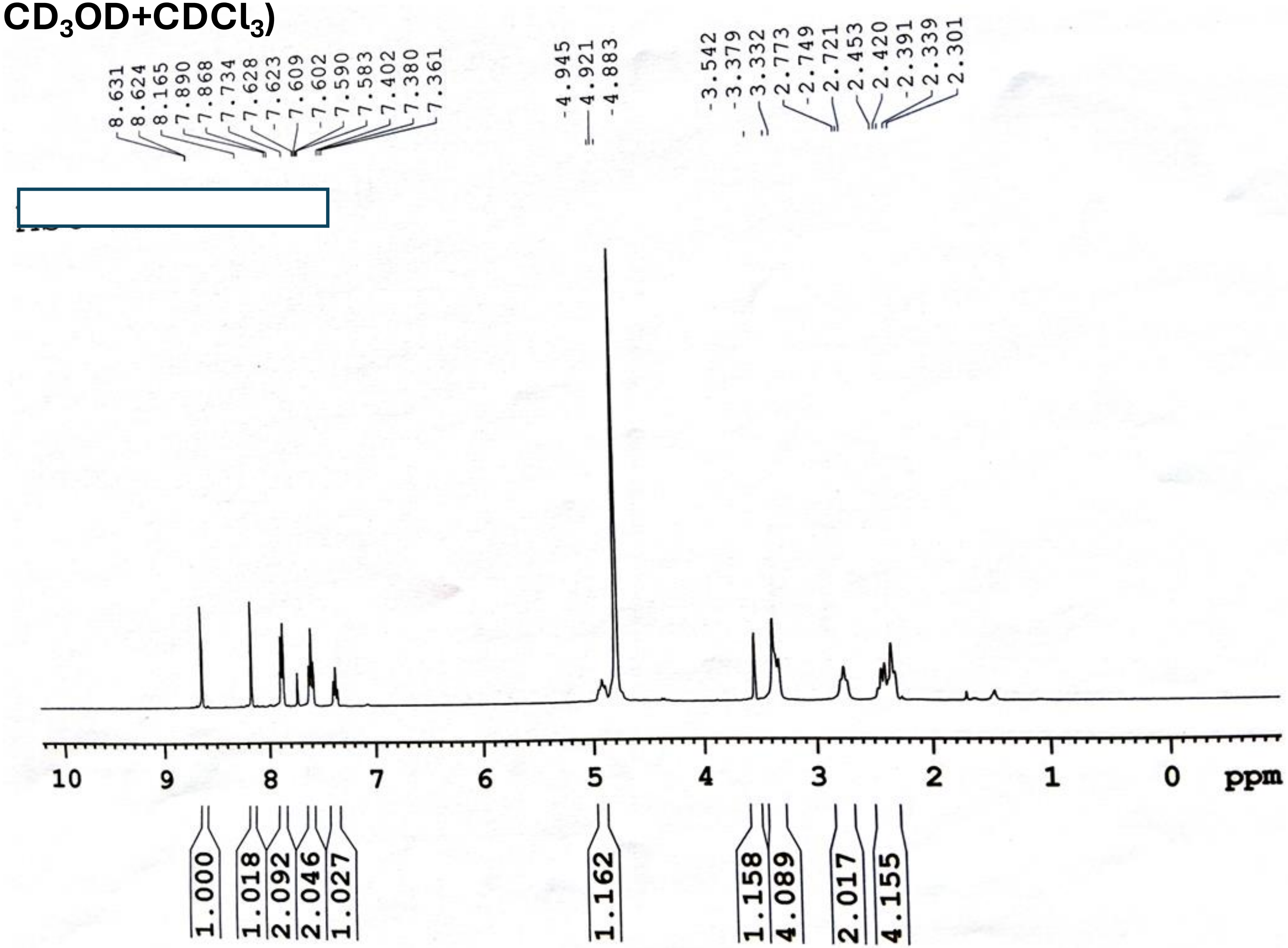

Image not found.

LC-UV Chromatogram

Method: Isocratic 80 20 for 10 min
Batch: U
Sample: MSU-SMQ-3-134
User: Admin
S/N: 000LC10009
Date: 06/27/2025 1:23 PM

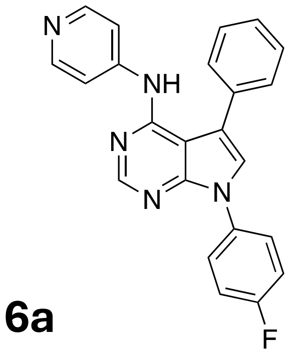

6a

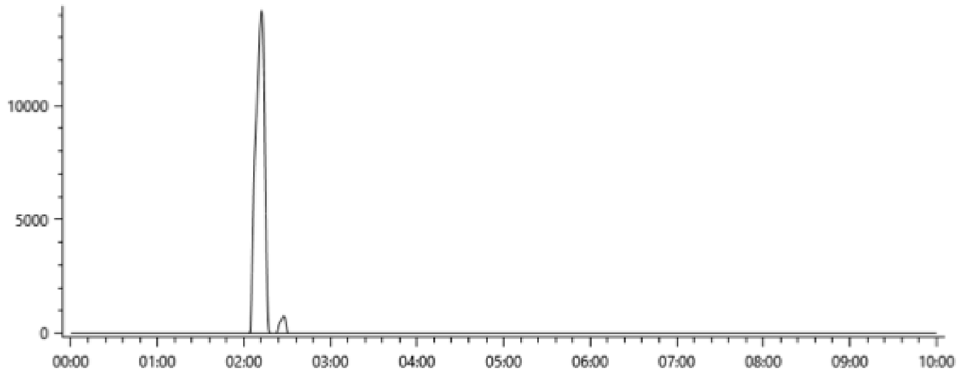

#	Compound	Retention Time	Area	% Area	Height	Conc.
1		02:12.2	102979.5	96.8	14205.37	0.000
2		02:27.4	3275.7	3.2	754.89	0.000

Image not found.

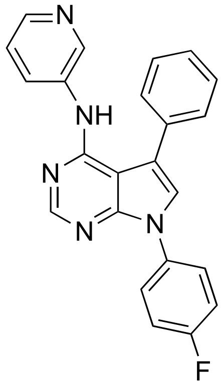

6b

LC-UV Chromatogram

Method: Isocratic 80 20 for 10 min
Batch: j
Sample: MSU-SMQ-4-022
User: Admin
S/N: 000LC10009
Date: 06/27/2025 10:31 AM

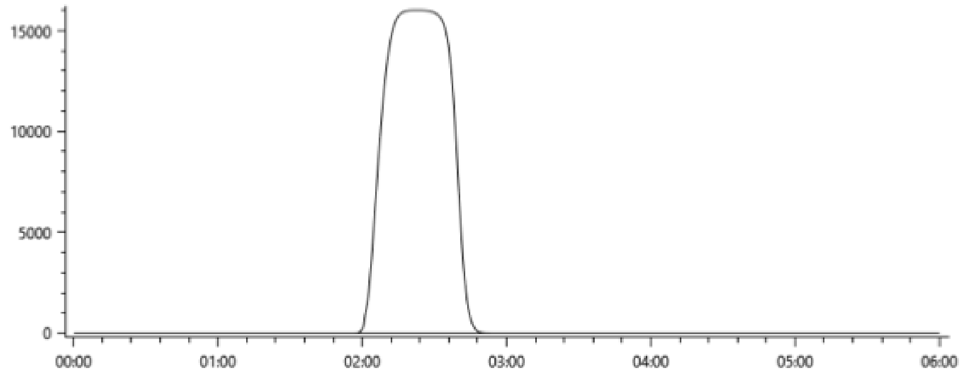

#	Compound	Retention Time	Area	% Area	Height	Conc.
1		02:22.8	528318.8	100.0	16017.11	0.000

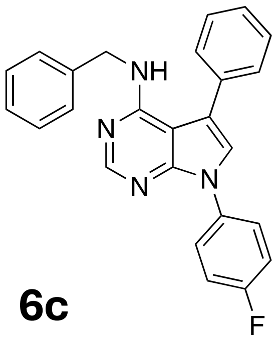

6c

Image not found.

LC-UV Chromatogram

Method: Isocratic 80 20 for 10 min
Batch: U
Sample: MSU-SMQ-3-123
User: Admin
S/N: 000LC10009
Date: 06/27/2025 1:07 PM

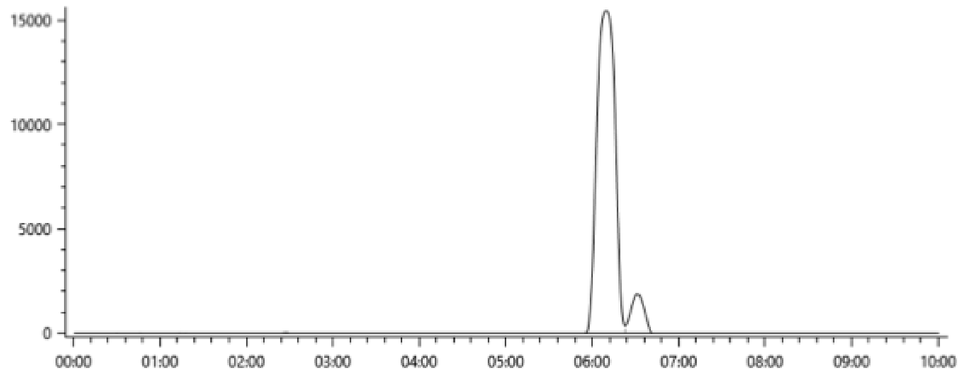

#	Compound	Retention Time	Area	% Area	Height	Conc.
1		06:09.6	227686.3	91.8	15472.51	0.000
2		06:31.6	20207.7	8.2	1896.80	0.000

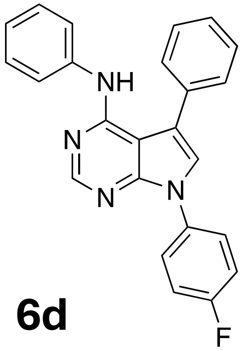

6d

Image not found.

LC-UV Chromatogram

Method: Isocratic 80 20 for 10 min
Batch: H
Sample: MSU-SMQ-3-130
User: Admin
S/N: 000LC10009
Date: 06/27/2025 11:28 AM

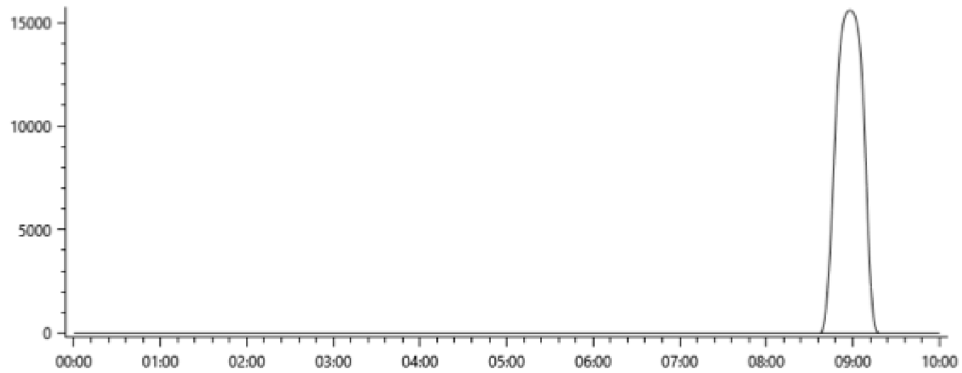

#	Compound	Retention Time	Area	% Area	Height	Conc.
1		08:57.8	347494.9	100.0	15607.54	0.000

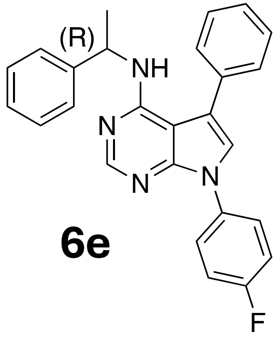

6e

LC-UV Chromatogram

Method: Isocratic 80 20 for 10 min
Batch: U
Sample: MSU-SMQ-3-146
User: Admin
S/N: 000LC10009
Date: 06/27/2025 1:34 PM

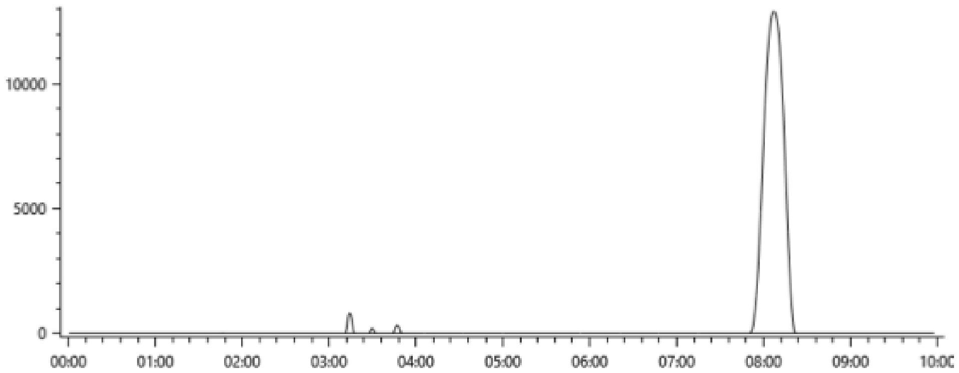

#	Compound	Retention Time	Area	% Area	Height	Conc.
1		03:14.4	2822.9	1.3	825.00	0.000
2		03:29.8	484.1	0.2	207.83	0.000
3		03:47.2	1113.3	0.5	357.58	0.000
4		08:07.2	207705.2	97.9	12967.13	0.000

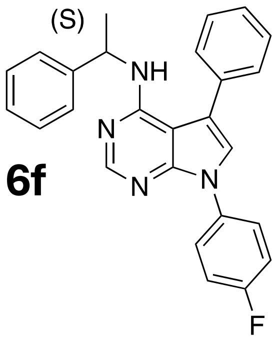

6f

Image not found.

LC-UV Chromatogram

Method: Isocratic 80 20 for 10 min
Batch: H
Sample: MSU-SMQ-3-147
User: Admin
S/N: 000LC10009
Date: 06/27/2025 12:10 PM

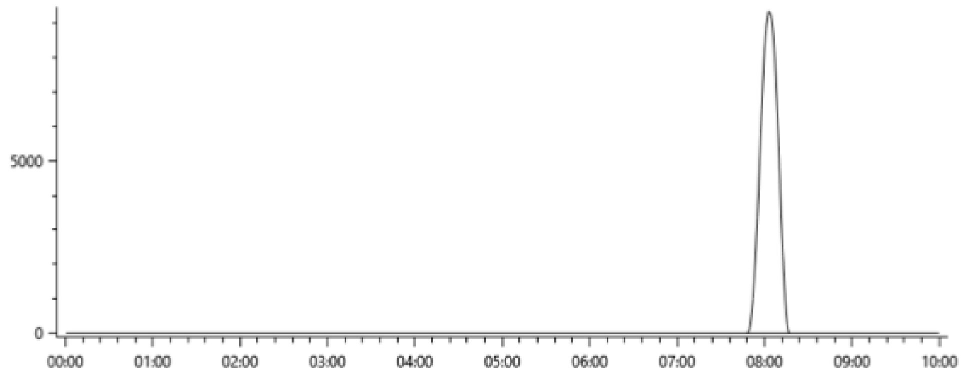

#	Compound	Retention Time	Area	% Area	Height	Conc.
1		08:03.4	132361.1	100.0	9380.95	0.000

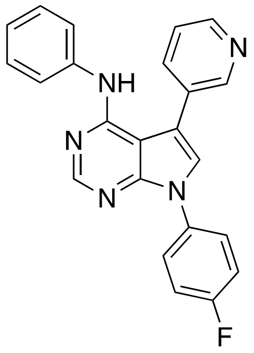

6g

Image not found.

LC-UV Chromatogram

Method: Isocratic 80 20 for 10 min
Batch: M
Sample: MSU-SMQ-3-154
User: Admin
S/N: 000LC10009
Date: 06/27/2025 6:06 PM

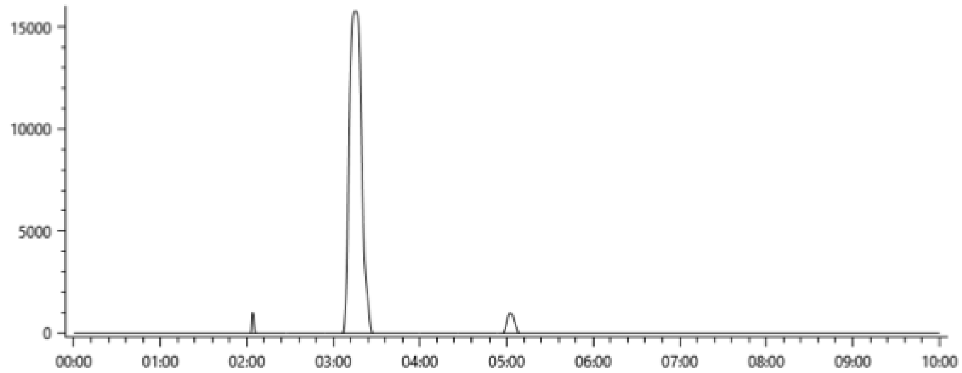

#	Compound	Retention Time	Area	% Area	Height	Conc.
1		02:04.2	1632.2	1.0	1057.24	0.000
2		03:15.2	156117.2	95.3	15826.50	0.000
3		05:02.8	6061.0	3.7	1007.58	0.000

Image not found.

LC-UV Chromatogram

Method: Isocratic 80 20 for 10 min
Batch: M
Sample: MSU-SMQ-3-153
User: Admin
S/N: 000LC10009
Date: 06/27/2025 5:56 PM

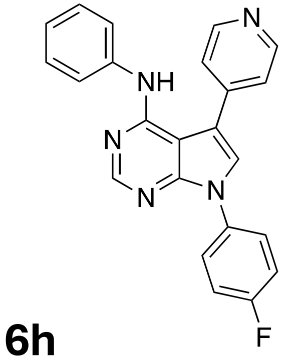

6h

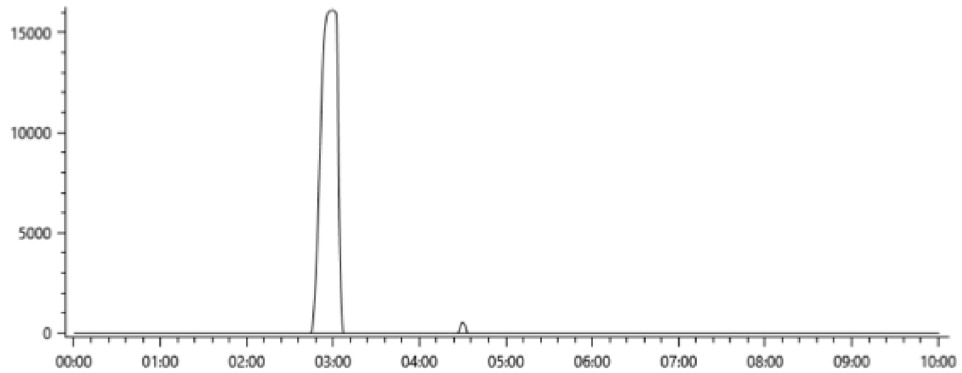

#	Compound	Retention Time	Area	% Area	Height	Conc.
1		02:59.8	219966.1	99.0	16112.93	0.000
2		04:30.2	2041.5	1.0	562.36	0.000

6i

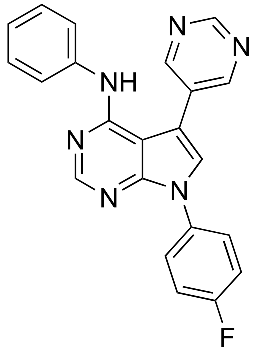

Image not found.

LC-UV Chromatogram

Method: Isocratic 80 20 for 10 min
Batch: kj
Sample: MSU-SMQ-3-171
User: Admin
S/N: 000LC10009
Date: 07/12/2025 6:12 PM

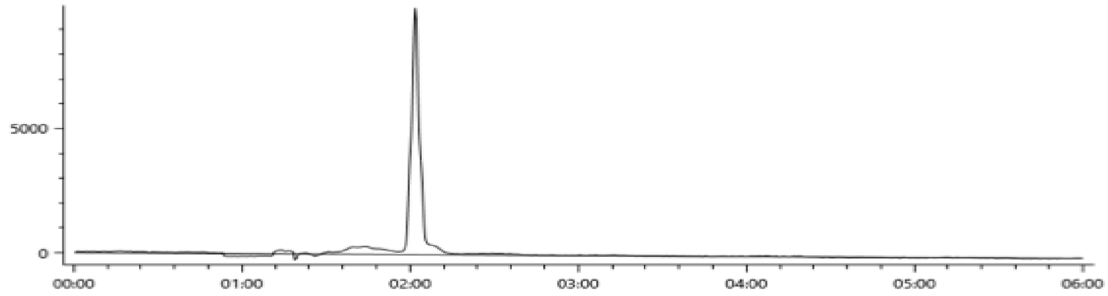

#	Compound	Retention Time	Area	% Area	Height	Conc.
1		02:01.8	43669.0	100.0	10292.86	0.000

Image not found.

LC-UV Chromatogram

Method: Isocratic 80 20 for 10 min
Batch: M
Sample: MSU-SMQ-3-151
User: Admin
S/N: 000LC10009
Date: 06/27/2025 5:45 PM

6j

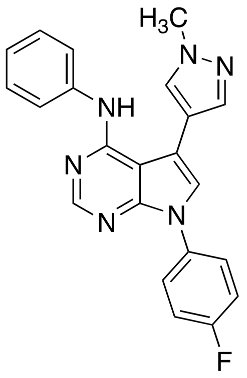

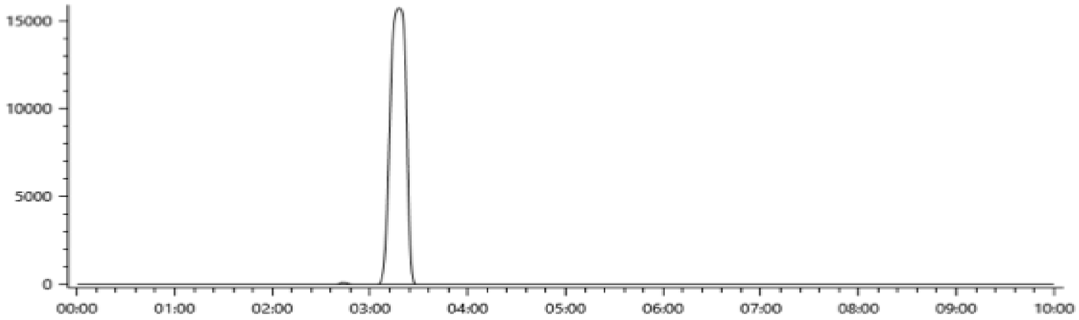

#	Compound	Retention Time	Area	% Area	Height	Conc.
1		03:17.8	179879.1	100.0	15740.55	0.000

Image not found.

6k

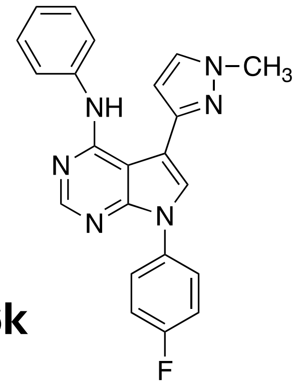

LC-UV Chromatogram

Method: Isocratic 80 20 for 10 min
Batch: M
Sample: MSU-SMQ-3-175
User: Admin
S/N: 000LC10009
Date: 06/27/2025 5:35 PM

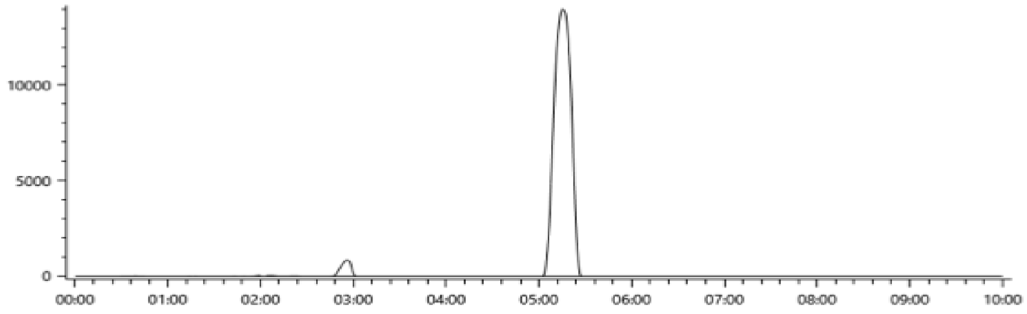

#	Compound	Retention Time	Area	% Area	Height	Conc.
1		02:56.2	7207.0	3.8	866.14	0.000
2		05:15.6	181952.4	96.2	14040.43	0.000

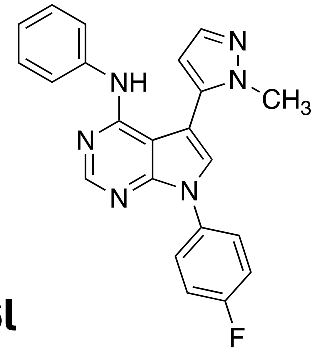

6l

Image not found.

LC-UV Chromatogram

Method: Isocratic 80 20 for 10 min
Batch: nmh
Sample: MSU-SMQ-3-152
User: Admin
S/N: 000LC10009
Date: 07/15/2025 8:33 PM

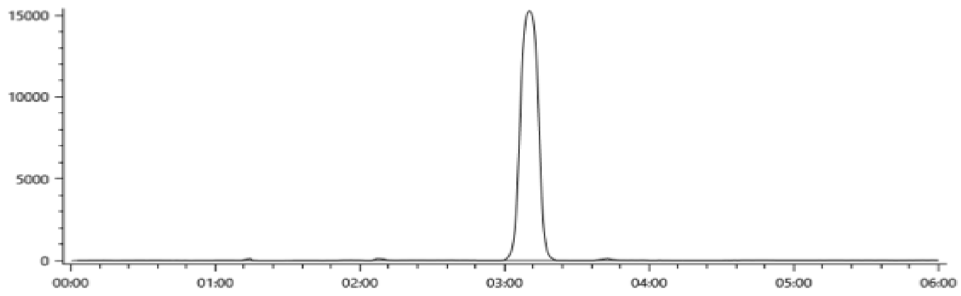

#	Compound	Retention Time	Area	% Area	Height	Conc.
1		03:10.4	129275.2	100.0	15294.18	0.000

Image not found.

LC-UV Chromatogram

Method: Isocratic 80 20 for 10 min
Batch: hu
Sample: MSU-SMQ-3-172
User: Admin
S/N: 000LC10009
Date: 06/30/2025 11:00 AM

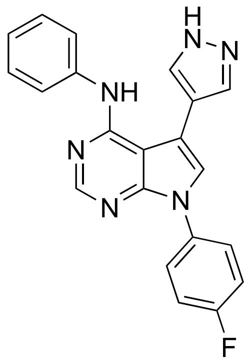

6
m

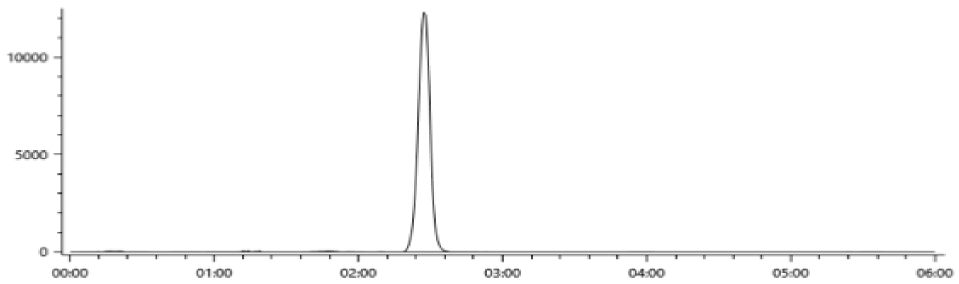

#	Compound	Retention Time	Area	% Area	Height	Conc.
1		02:27.4	72515.6	100.0	12389.44	0.000

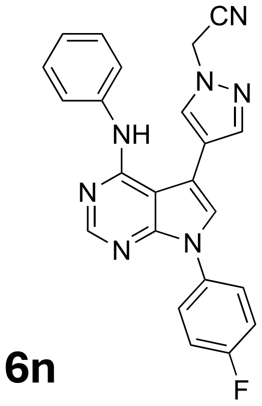

6n

Image not found.

LC-UV Chromatogram

Method: Isocratic 80 20 for 10 min
Batch: hu
Sample: MSU-SMQ-3-188
User: Admin
S/N: 000LC10009
Date: 06/30/2025 11:07 AM

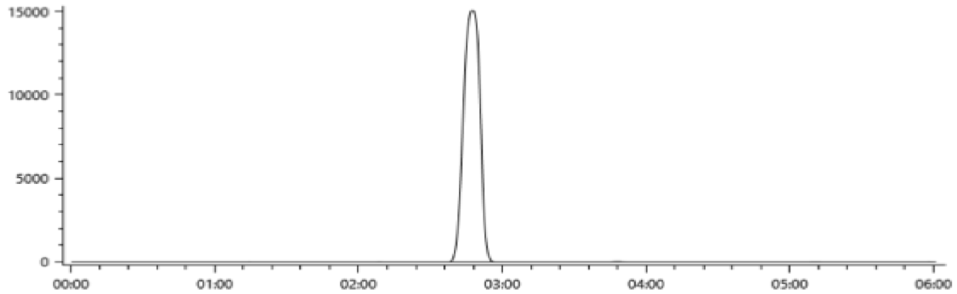

#	Compound	Retention Time	Area	% Area	Height	Conc.
1		02:47.6	120320.1	100.0	15129.78	0.000

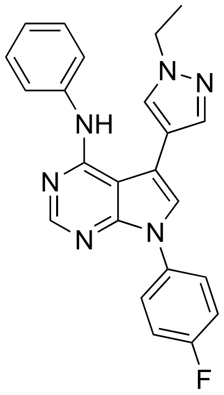

6o

Image not found.

LC-UV Chromatogram

Method: Isocratic 80 20 for 10 min
Batch: j
Sample: MSU-SMQ-3-189
User: Admin
S/N: 000LC10009
Date: 06/30/2025 11:52 AM

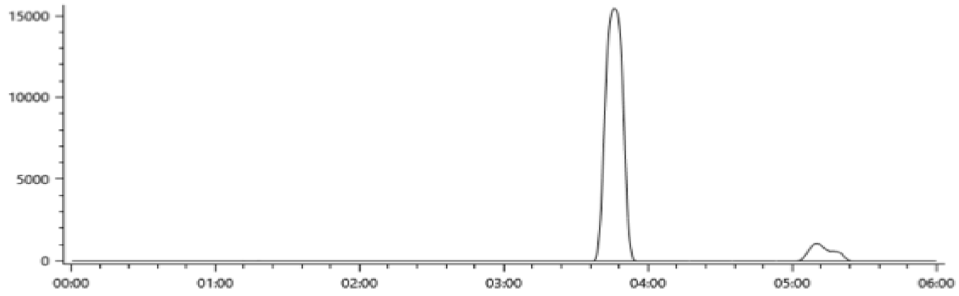

#	Compound	Retention Time	Area	% Area	Height	Conc.
1		03:46.0	132121.9	91.9	15461.64	0.000
2		05:10.2	12259.9	8.1	1064.92	0.000

Image not found.

LC-UV Chromatogram

Method: Isocratic 80 20 for 10 min
Batch: hu
Sample: MSU-SMQ-4-030
User: Admin
S/N: 000LC10009
Date: 06/30/2025 11:20 AM

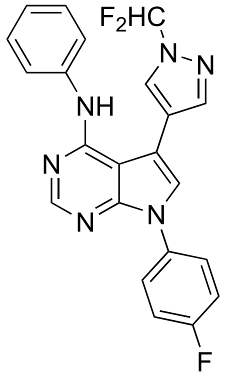

6p

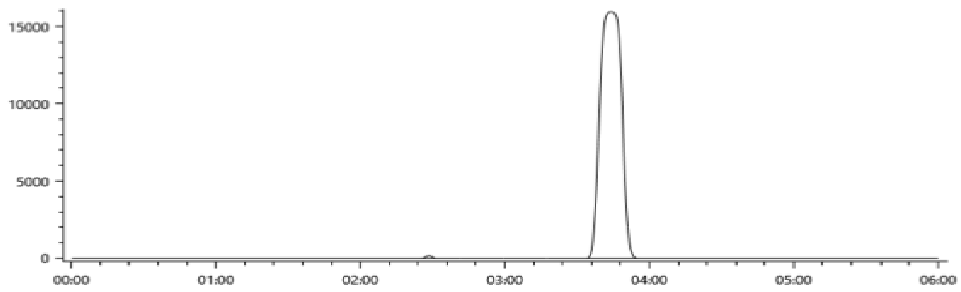

#	Compound	Retention Time	Area	% Area	Height	Conc.
1		02:28.6	532.2	0.3	183.20	0.000
2		03:44.2	166373.5	99.7	15962.08	0.000

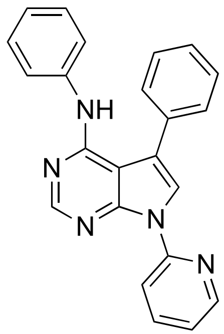

10a

Image not found.

LC-UV Chromatogram

Method: Isocratic 80 20 for 10 min
Batch: kj
Sample: MSU-SMQ-4-053
User: Admin
S/N: 000LC10009
Date: 07/12/2025 6:18 PM

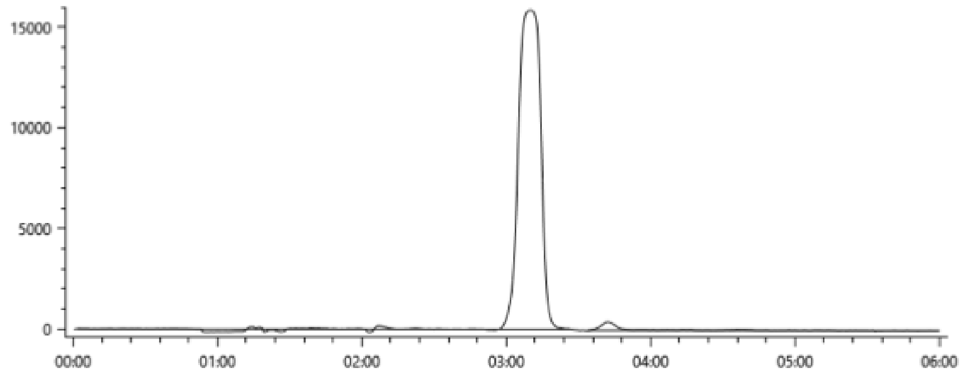

#	Compound	Retention Time	Area	% Area	Height	Conc.
1		03:10.0	167652.7	98.5	16034.28	0.000
2		03:42.2	2526.6	1.5	515.61	0.000

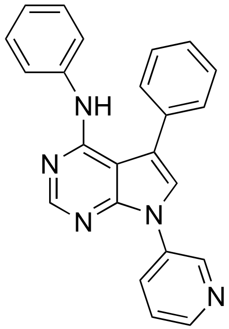

10b

Image not found.

LC-UV Chromatogram

Method: Isocratic 80 20 for 10 min
Batch: iu
Sample: MSU-SMQ-4-044
User: Admin
S/N: 000LC10009
Date: 07/02/2025 2:47 PM

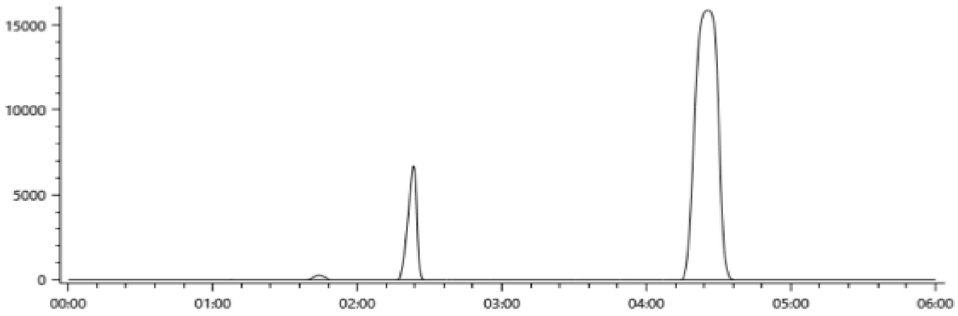

#	Compound	Retention Time	Area	% Area	Height	Conc.
1		02:23.2	30223.4	14.8	6706.92	0.000
2		04:25.6	173799.7	85.2	15887.06	0.000

Image not found.

LC-UV Chromatogram

Method: Isocratic 80 20 for 10 min
Batch: uy
Sample: MSU-SMQ-4-036
User: Admin
S/N: 000LC10009
Date: 07/02/2025 1:37 PM

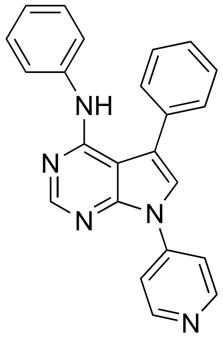

10c

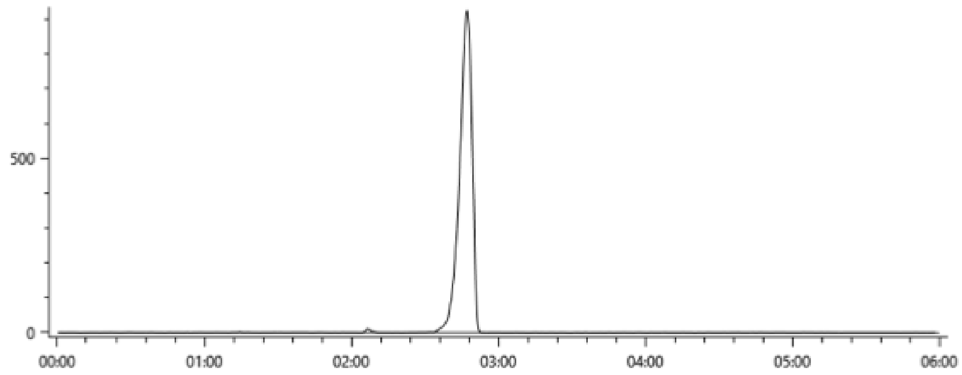

#	Compound	Retention Time	Area	% Area	Height	Conc.
1		02:47.4	5314.4	100.0	932.03	0.000

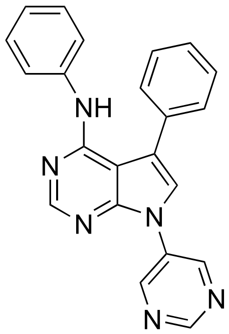

10d

Image not found.

LC-UV Chromatogram

Method: Isocratic 80 20 for 10 min
Batch: uy
Sample: MSU-SMQ-4-049
User: Admin
S/N: 000LC10009
Date: 06/30/2025 2:12 PM

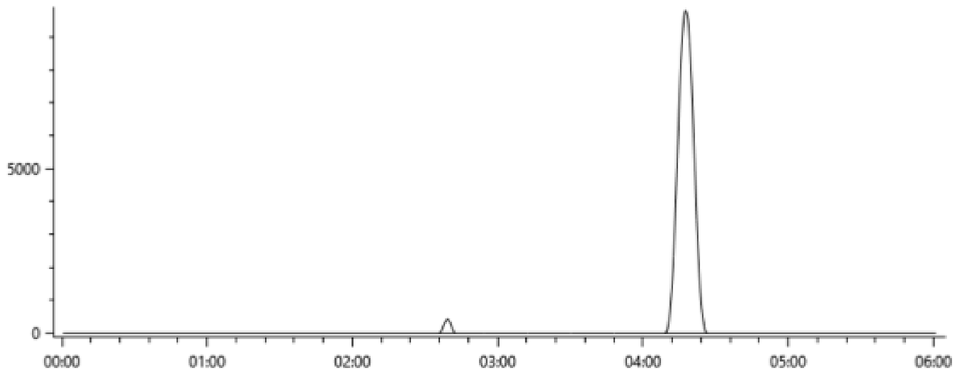

#	Compound	Retention Time	Area	% Area	Height	Conc.
1		02:39.4	1535.8	2.0	439.15	0.000
2		04:17.6	75228.7	98.0	9812.77	0.000

Image not found.

LC-UV Chromatogram

Method: Isocratic 80 20 for 10 min
Batch: uy
Sample: MSU-SMQ-4-048
User: Admin
S/N: 000LC10009
Date: 06/30/2025 2:06 PM

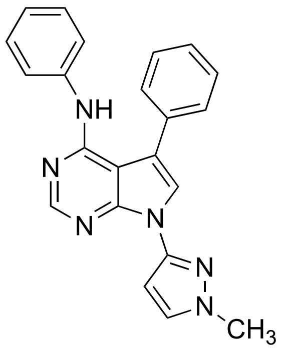

10e

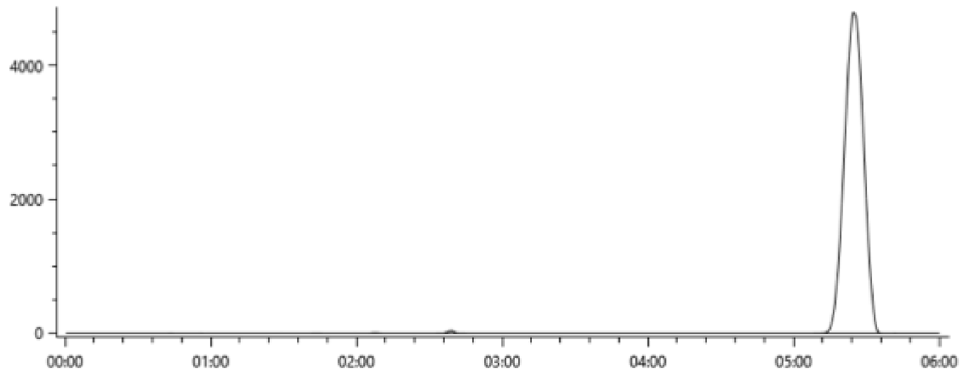

#	Compound	Retention Time	Area	% Area	Height	Conc.
1		05:25.0	44463.7	100.0	4826.38	0.000

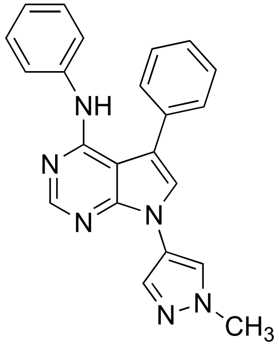

10f

Image not found.

LC-UV Chromatogram

Method: Isocratic 80 20 for 10 min
Batch: uy
Sample: MSU-SMQ-4-045
User: Admin
S/N: 000LC10009
Date: 07/02/2025 1:31 PM

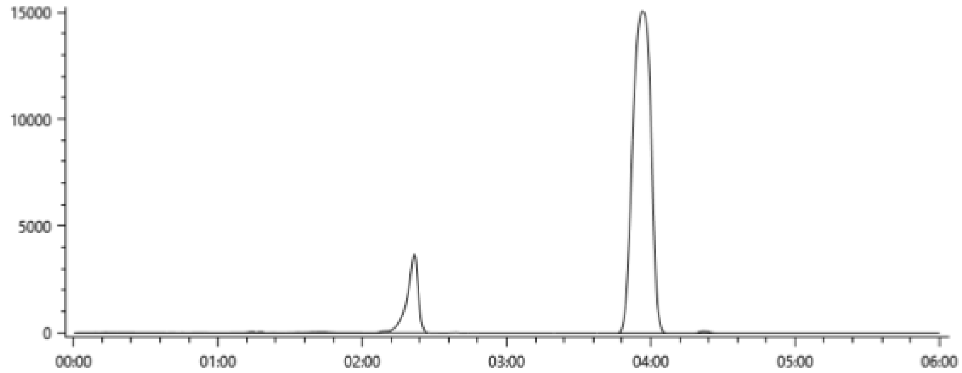

#	Compound	Retention Time	Area	% Area	Height	Conc.
1		02:21.6	19876.1	13.1	3702.67	0.000
2		03:56.6	132649.8	86.9	15117.12	0.000

Image not found.

LC-UV Chromatogram

Method: Isocratic 80 20 for 10 min
Batch: uy
Sample: MSU-SMQ-4-055
User: Admin
S/N: 000LC10009
Date: 07/02/2025 1:44 PM

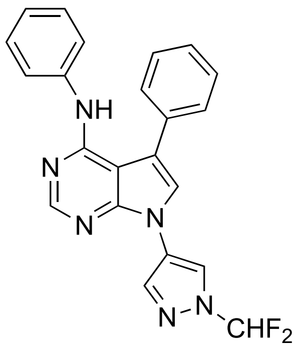

10g

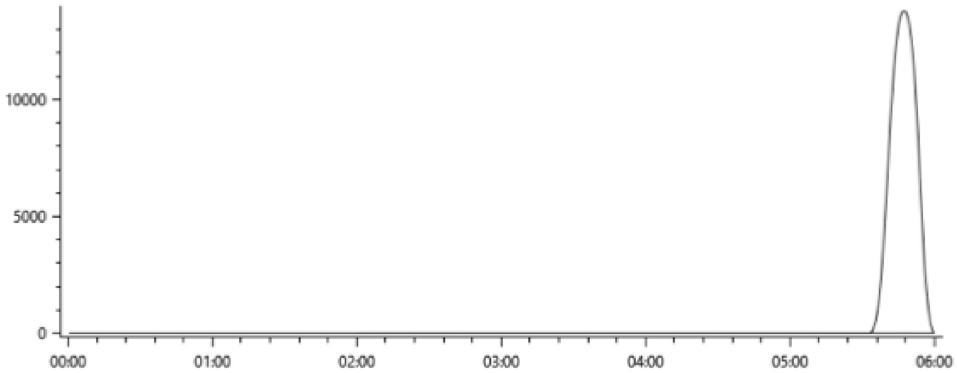

#	Compound	Retention Time	Area	% Area	Height	Conc.
1		05:47.4	181996.1	100.0	13832.90	0.000

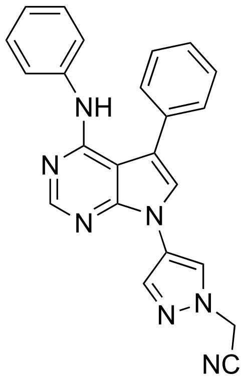

10h

Image not found.

LC-UV Chromatogram

Method: Isocratic 80 20 for 10 min
Batch: II
Sample: MSU-SMQ-4-056
User: Admin
S/N: 000LC10009
Date: 07/12/2025 7:14 PM

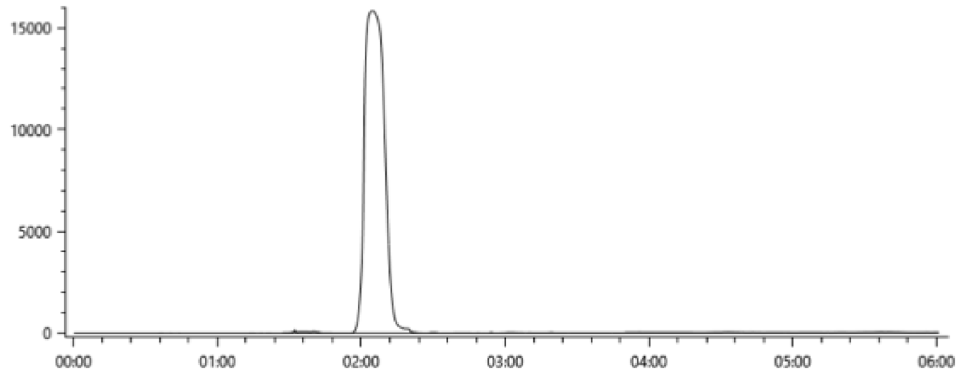

#	Compound	Retention Time	Area	% Area	Height	Conc.
1		02:04.6	154006.7	100.0	15868.25	0.000

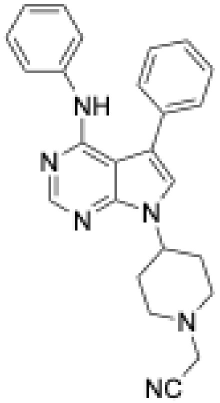

12a

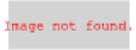

LC-UV Chromatogram

Method: Isocratic 80 20 for 10 min
Batch: uy
Sample: MSU-SMQ-4-119
User: Admin
S/N: 000LC10009
Date: 07/02/2025 2:03 PM

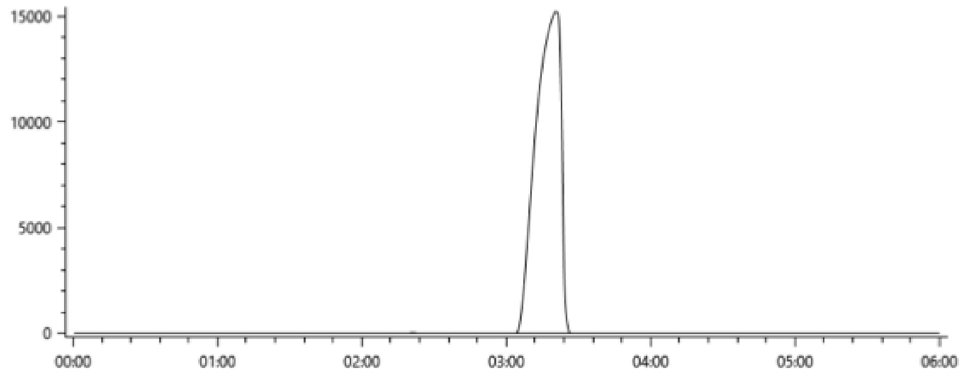

#	Compound	Retention Time	Area	% Area	Height	Conc.
1		03:20.4	186037.0	100.0	15256.62	0.000

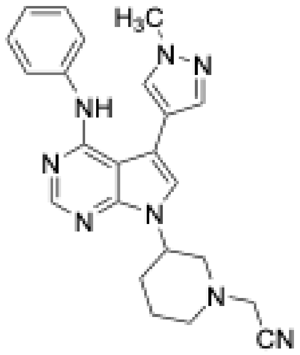

12b

Image not found.

LC-UV Chromatogram

Method: Isocratic 80 20 for 10 min
Batch: io
Sample: MSU-SMQ-4-144
User: Admin
S/N: 000LC10009
Date: 07/02/2025 3:45 PM

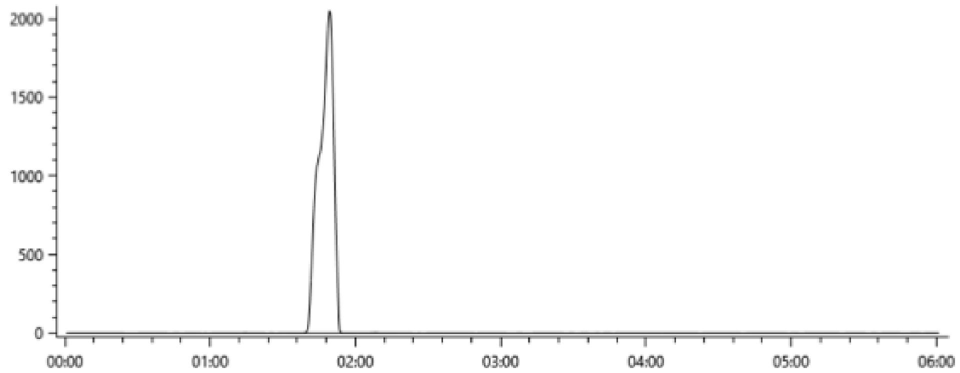

#	Compound	Retention Time	Area	% Area	Height	Conc.
1		01:49.2	14167.3	100.0	2050.05	0.000

Image not found.

LC-UV Chromatogram

Method: Isocratic 80 20 for 10 min
Batch: jk
Sample: MSU-SMQ-4-143
User: Admin
S/N: 000LC10009
Date: 07/02/2025 4:33 PM

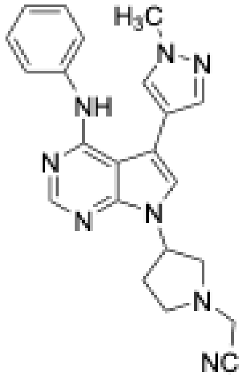

12c

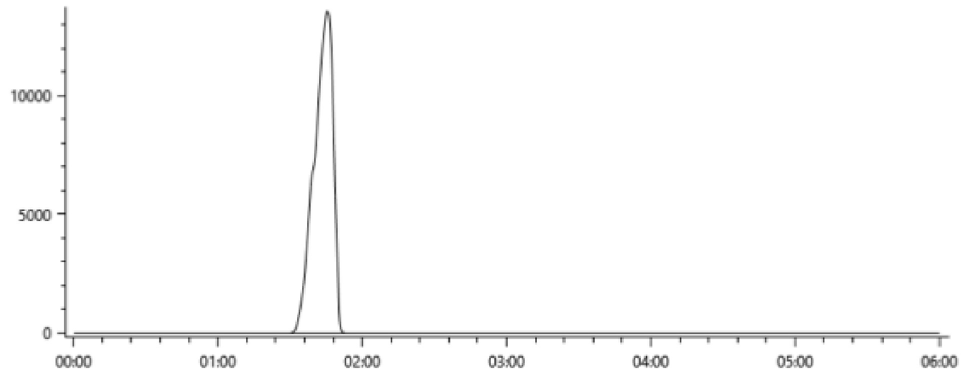

#	Compound	Retention Time	Area	% Area	Height	Conc.
1		01:45.4	125817.9	100.0	13562.68	0.000

Image not found.

LC-UV Chromatogram

Method: Isocratic 80 20 for 10 min
Batch: uy
Sample: MSU-SMQ-4-099
User: Admin
S/N: 000LC10009
Date: 07/02/2025 1:57 PM

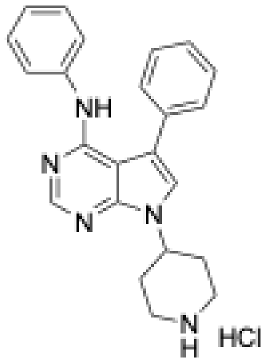

13

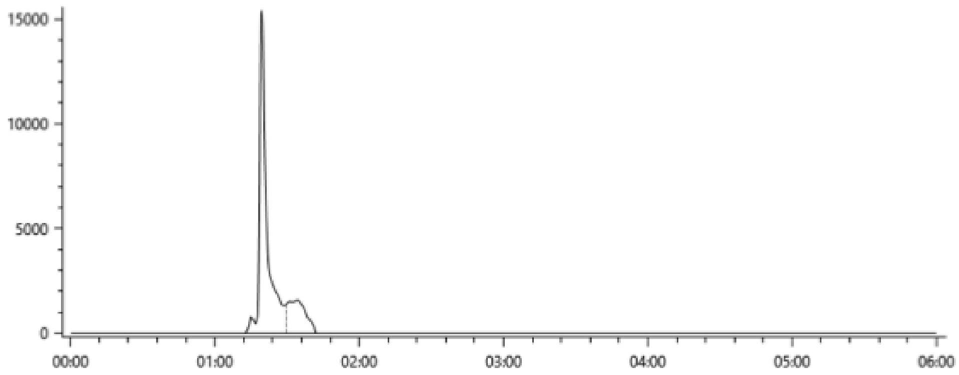

#	Compound	Retention Time	Area	% Area	Height	Conc.
1		01:19.8	69449.7	95.6	14760.07	0.000
2		01:34.2	14476.8	4.4	1606.27	0.000

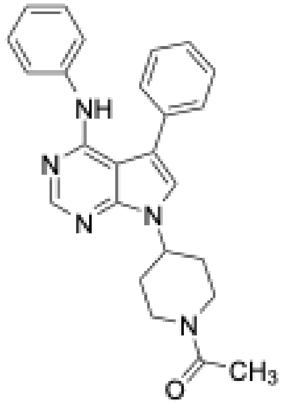

14a

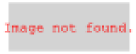

LC-UV Chromatogram

Method: Isocratic 80 20 for 10 min
Batch: jh
Sample: MSU-SMQ-4-106
User: Admin
S/N: 000LC10009
Date: 07/03/2025 1:31 PM

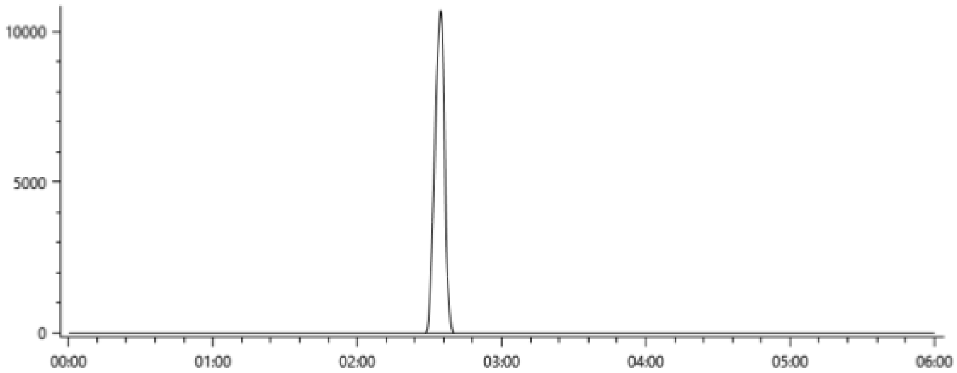

#	Compound	Retention Time	Area	% Area	Height	Conc.
1		02:34.6	51008.4	100.0	10711.93	0.000

Image not found.

LC-UV Chromatogram

Method: Isocratic 80 20 for 10 min
Batch: umn
Sample: MSU-SMQ-4-107
User: Admin
S/N: 000LC10009
Date: 07/03/2025 12:21 PM

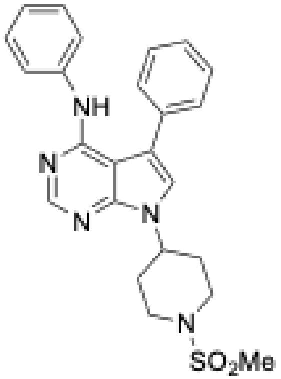

14b

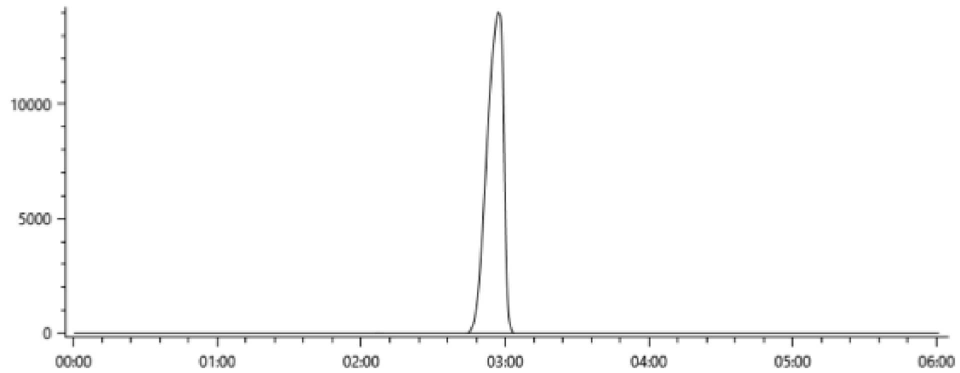

#	Compound	Retention Time	Area	% Area	Height	Conc.
1		02:57.0	113314.7	100.0	14030.70	0.000

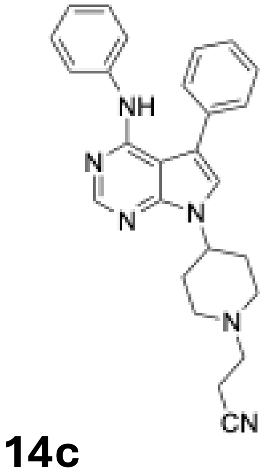

14c

Image not found.

LC-UV Chromatogram

Method: Isocratic 80 20 for 10 min
Batch: nmh
Sample: MSU-SMQ-4-120
User: Admin
S/N: 000LC10009
Date: 07/15/2025 8:27 PM

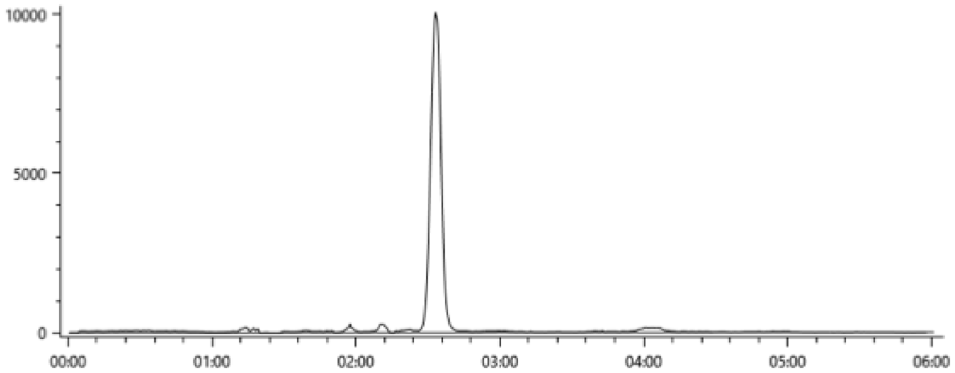

#	Compound	Retention Time	Area	% Area	Height	Conc.
1		02:33.4	66160.5	100.0	10122.63	0.000

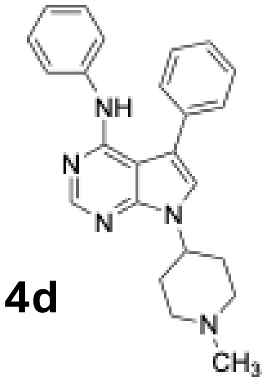

14d

Image not found.

LC-UV Chromatogram

Method: Isocratic 80 20 for 10 min
Batch: nmh
Sample: MSU-SMQ-4-108
User: Admin
S/N: 000LC10009
Date: 07/15/2025 8:20 PM

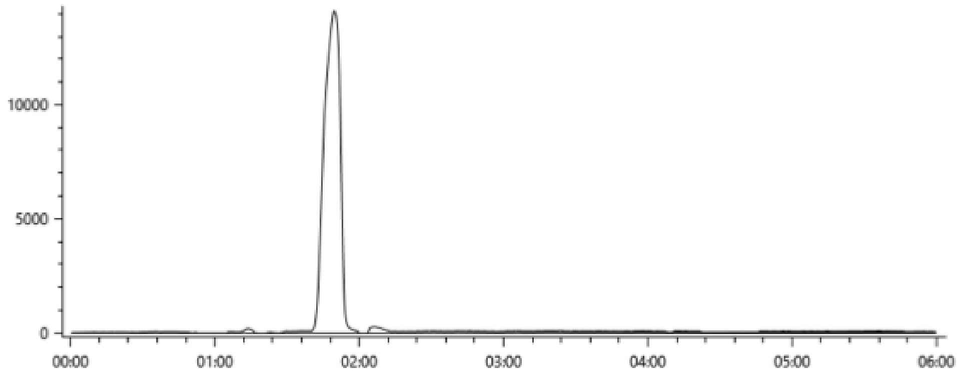

#	Compound	Retention Time	Area	% Area	Height	Conc.
1		01:49.6	114761.4	98.8	14160.23	0.000
2		02:06.4	22045.9	1.2	277.30	0.000

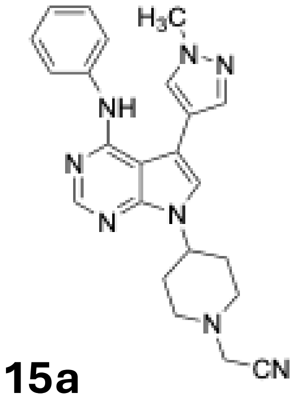

15a

Image not found.

LC-UV Chromatogram

Method: Isocratic 80 20 for 10 min
Batch: uy
Sample: MSU-SMQ-4-152
User: Admin
S/N: 000LC10009
Date: 07/15/2025 7:09 PM

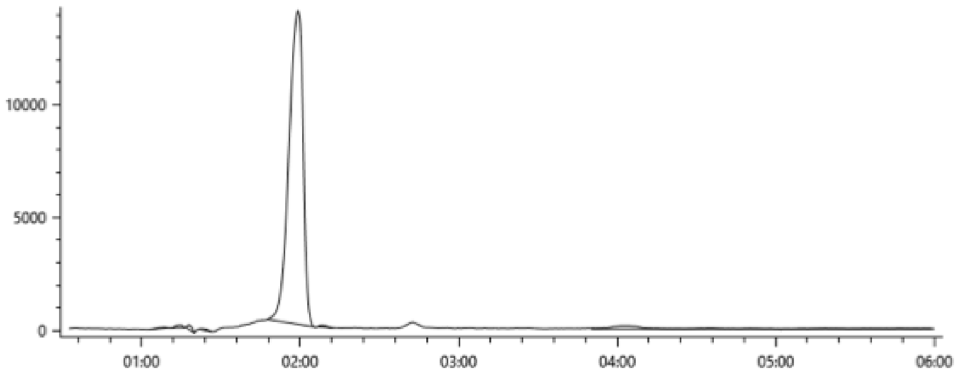

#	Compound	Retention Time	Area	% Area	Height	Conc.
1		01:08.2	165.9	0.2	266.03	0.000
2		01:14.2	373.1	0.4	355.11	0.000
3		01:18.0	569.6	0.6	370.42	0.000
4		01:22.8	180.5	0.2	199.04	0.000
5		01:59.0	86329.2	96.8	14257.91	0.000
6		02:08.2	295.2	0.3	312.24	0.000
7		04:02.2	2724.9	1.5	148.14	0.000

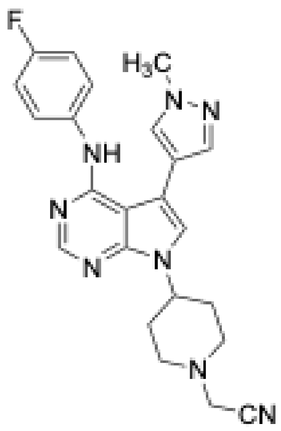

15b

Image not found.

LC-UV Chromatogram

Method: Isocratic 80 20 for 10 min
Batch: io
Sample: MSU-SMQ-4-162
User: Admin
S/N: 000LC10009
Date: 07/02/2025 3:58 PM

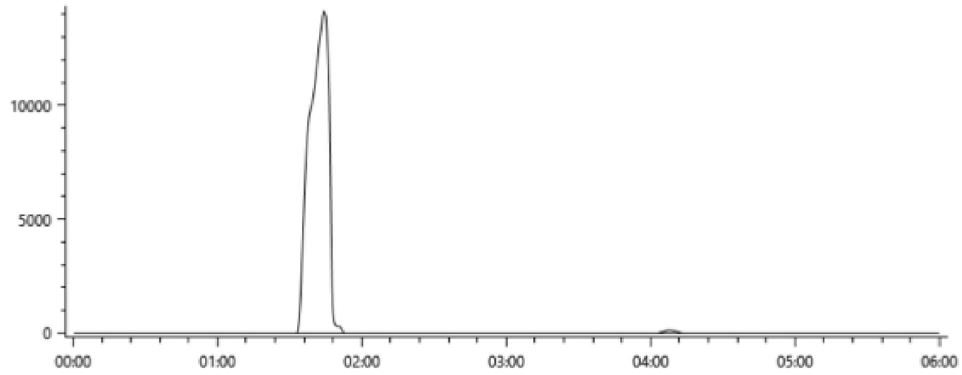

#	Compound	Retention Time	Area	% Area	Height	Conc.
1		01:44.2	133922.1	100.0	14145.97	0.000

Image not found.

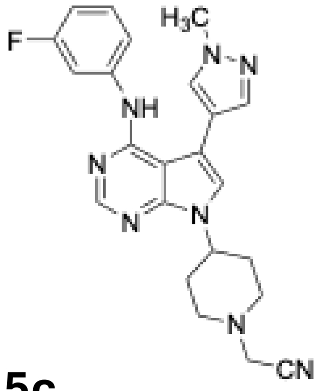

15c

LC-UV Chromatogram

Method: Isocratic 80 20 for 10 min
Batch: II
Sample: MSU-SMQ-4-167
User: Admin
S/N: 000LC10009
Date: 07/12/2025 7:27 PM

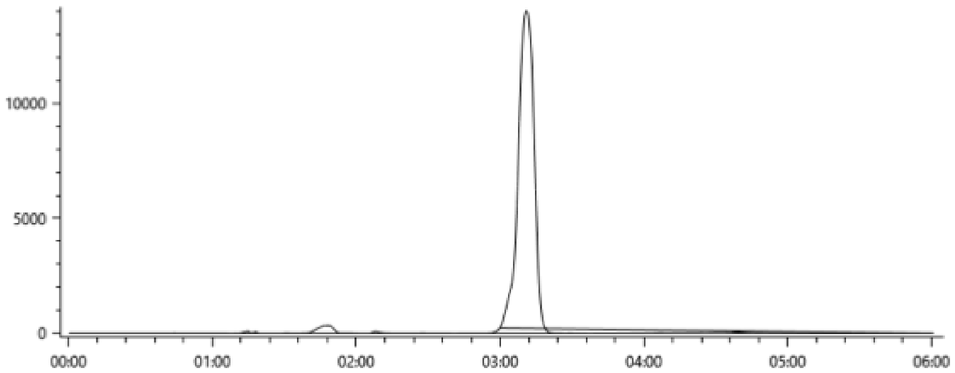

#	Compound	Retention Time	Area	% Area	Height	Conc.
1		01:47.6	3914.4	3.1	348.66	0.000
2		02:08.4	270.9	0.3	82.90	0.000
3		03:11.0	90346.5	96.6	14069.57	0.000

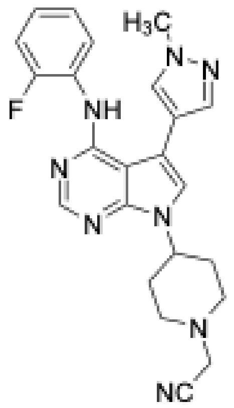

15d

LC-UV Chromatogram

Method: Isocratic 80 20 for 10 min
Batch: jk
Sample: MSU-SMQ-5-036
User: Admin
S/N: 000LC10009
Date: 07/02/2025 4:46 PM

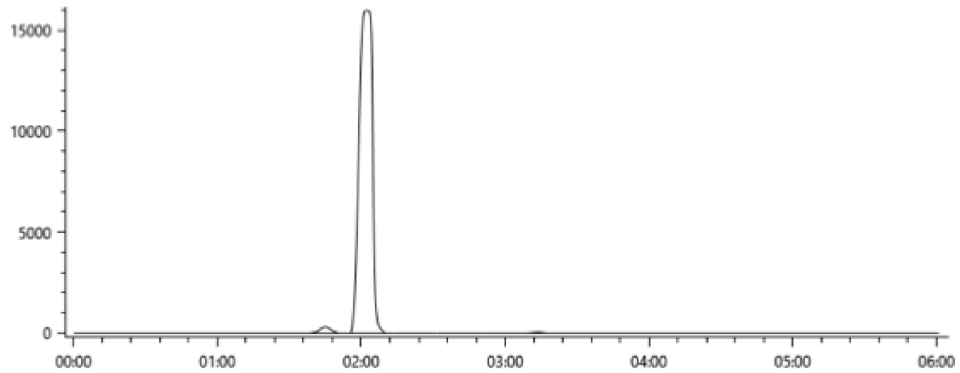

#	Compound	Retention Time	Area	% Area	Height	Conc.
1		01:45.0	1807.8	1.7	315.81	0.000
2		02:02.4	102523.1	98.0	15990.86	0.000
3		03:13.6	327.5	0.3	61.91	0.000

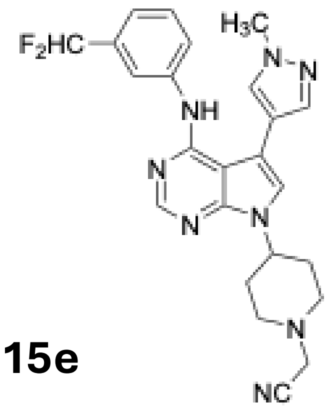

15e

LC-UV Chromatogram

Method: Isocratic 80 20 for 10 min
Batch: jh
Sample: MSU-SMQ-5-045
User: Admin
S/N: 000LC10009
Date: 07/03/2025 1:24 PM

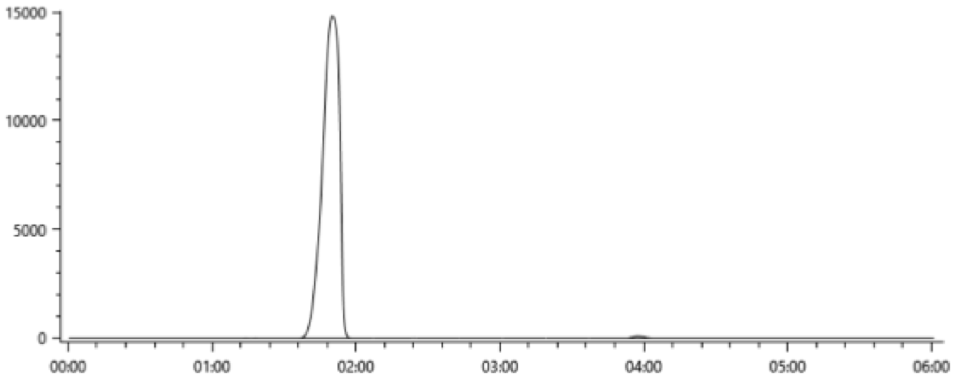

#	Compound	Retention Time	Area	% Area	Height	Conc.
1		01:50.4	122759.8	99.5	14866.44	0.000
2		03:57.6	543.5	0.5	101.88	0.000

Image not found.

LC-UV Chromatogram

Method: Isocratic 80 20 for 10 min
Batch: uy
Sample: MSU-SMQ-5-120
User: Admin
S/N: 000LC10009
Date: 07/15/2025 7:15 PM

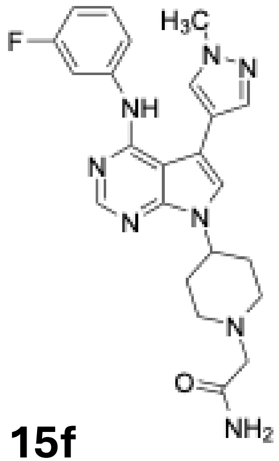

15f

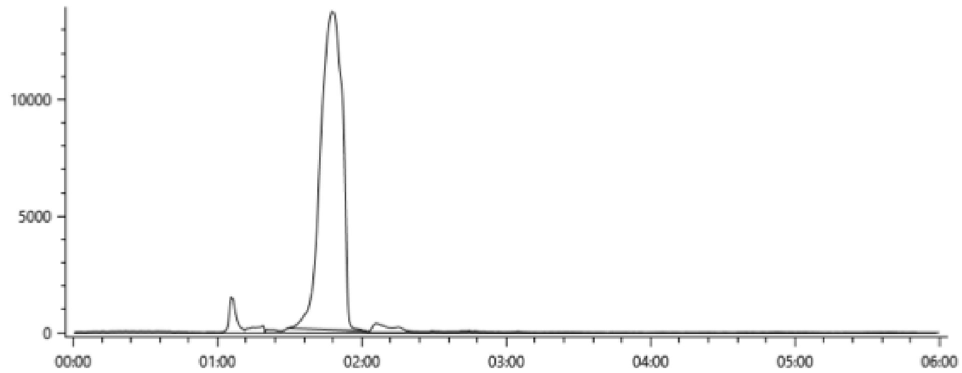

#	Compound	Retention Time	Area	% Area	Height	Conc.
1		01:06.2	9241.1	2.6	1616.34	0.000
2		01:47.8	142655.7	96.6	13847.53	0.000
3		02:06.4	6358.1	0.9	389.13	0.000

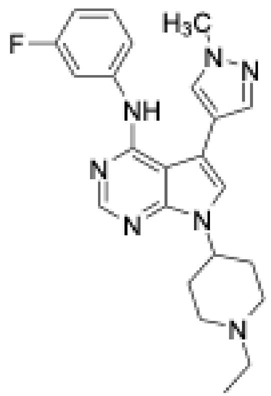

15g

Image not found.

LC-UV Chromatogram

Method: Isocratic 80 20 for 10 min
Batch: II
Sample: MSU-SMQ-5-145
User: Admin
S/N: 000LC10009
Date: 07/12/2025 7:40 PM

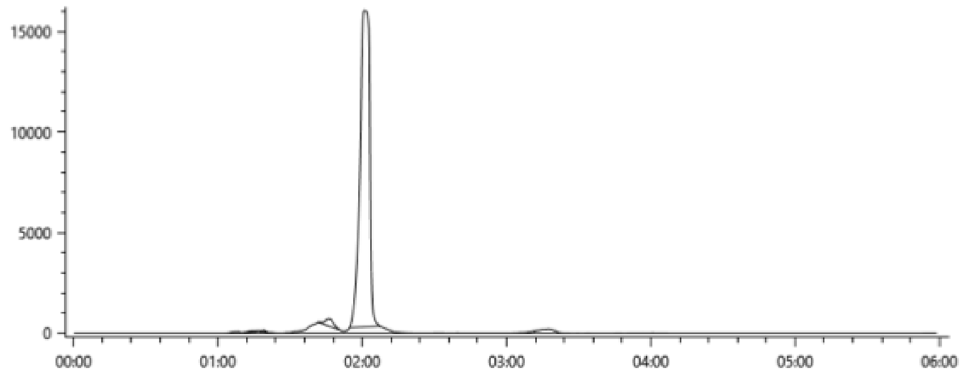

#	Compound	Retention Time	Area	% Area	Height	Conc.
1		01:07.2	445.7	-0.8	81.36	0.000
2		01:14.2	79.2	0.1	115.66	0.000
3		01:18.4	81.6	0.1	93.82	0.000
4		01:46.0	1554.0	2.0	735.35	0.000
5		02:01.2	74459.7	97.5	16061.50	0.000
6		03:17.2	1567.6	1.1	207.34	0.000

Image not found.

LC-UV Chromatogram

Method: Isocratic 80 20 for 10 min
Batch: umn
Sample: MSU-SMQ-5-105
User: Admin
S/N: 000LC10009
Date: 07/03/2025 12:47 PM

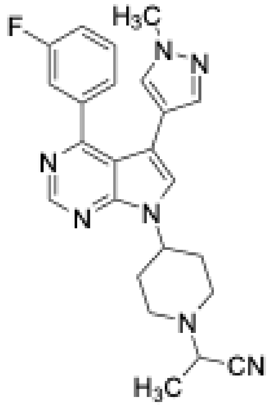

15h

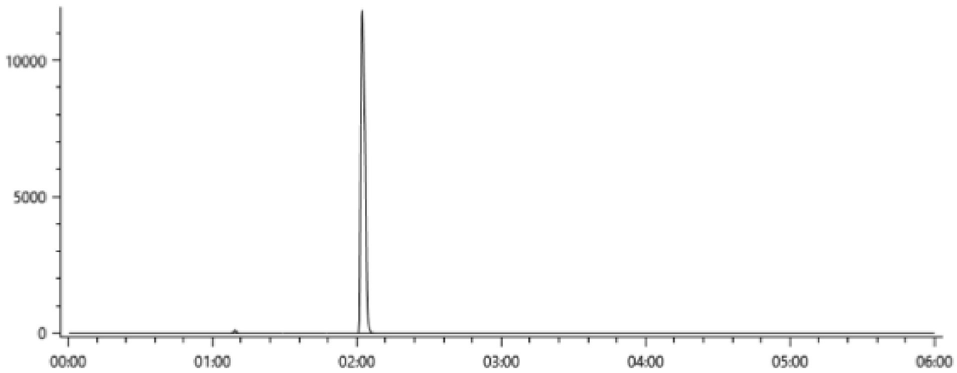

#	Compound	Retention Time	Area	% Area	Height	Conc.
1		01:09.4	120.9	0.5	124.99	0.000
2		02:02.4	24423.3	99.5	11381.50	0.000

LC-UV Chromatogram

Method: Isocratic 80 20 for 10 min
Batch: MSU-RK-5-073
Sample: MSU-RK-5-073
User: Admin
S/N: 000LC10009
Date: 10/07/2025 1:05 PM

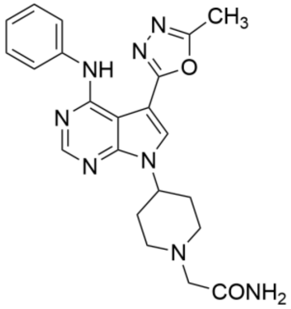

18

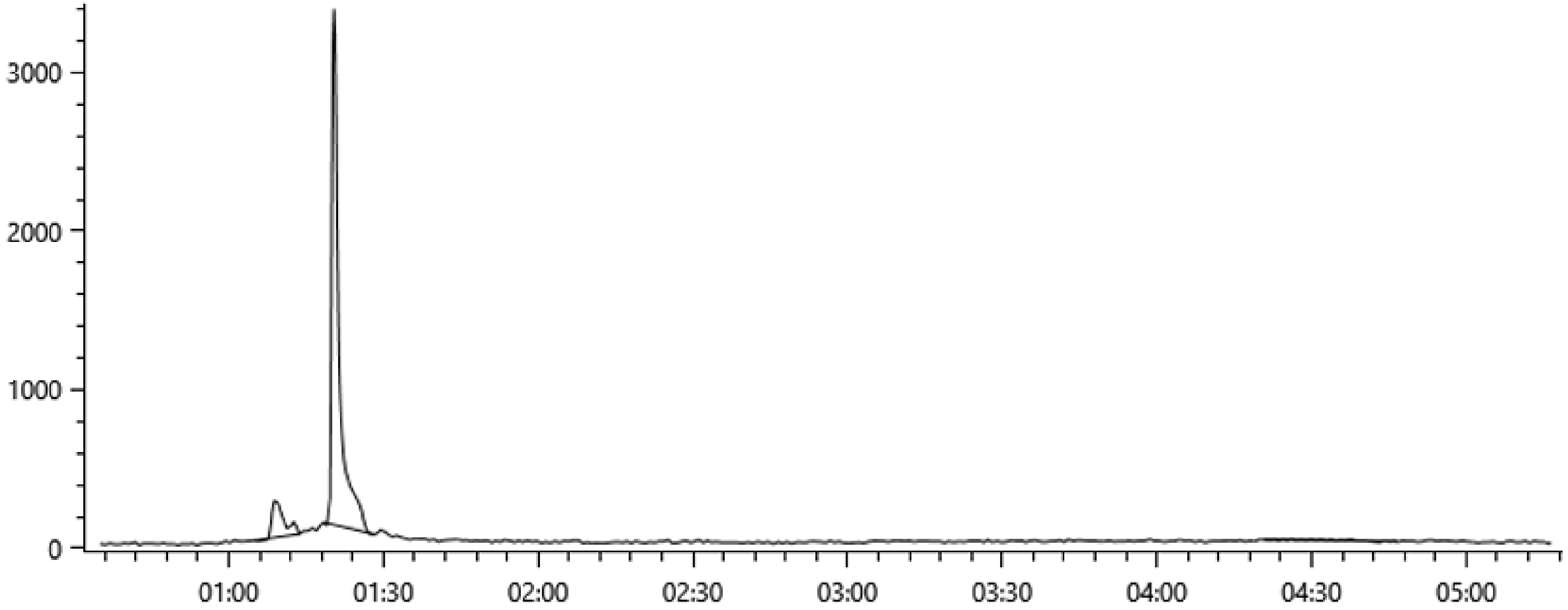

#	Compound	Retention Time	Area	% Area	Height	Conc.
1		01:09.4	612.0	10.3	310.52	0.000
2		01:20.6	5306.6	89.5	3267.99	0.000
3		04:20.4	8.9	0.2	76.67	0.000

LC-UV Chromatogram

Method: Isocratic 80 20 for 10 min
Batch: MSU-RK-5-085
Sample: MSU-RK-5-085
User: Admin
S/N: 000LC10009
Date: 09/10/2025 12:34 PM

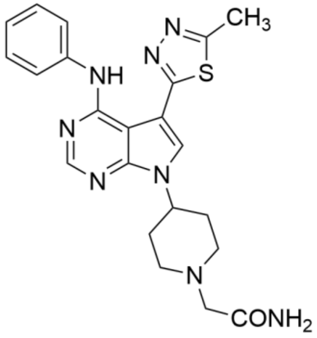

19

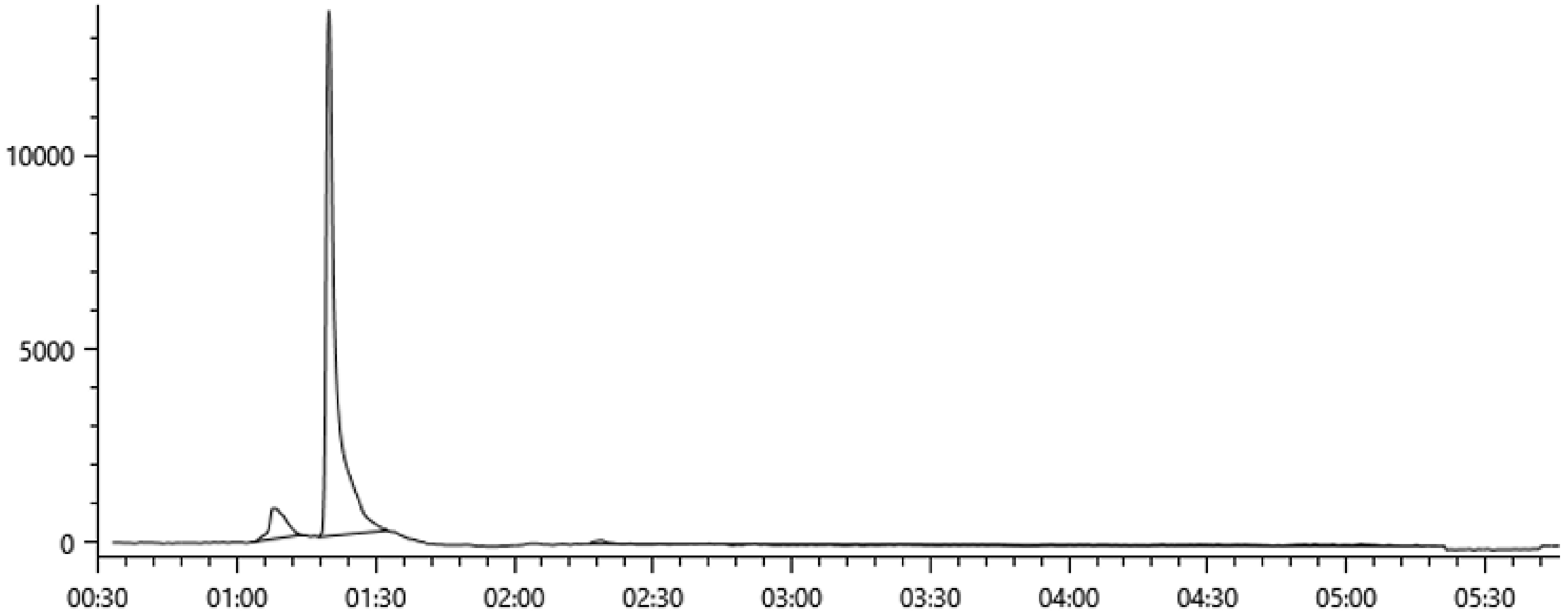

#	Compound	Retention Time	Area	% Area	Height	Conc.
1		01:08.6	3269.1	8.8	950.25	0.000
2		01:20.2	33839.2	90.7	12598.42	0.000
3		02:18.2	-117.5	0.5	183.76	0.000

LC-UV Chromatogram

Method: Isocratic 80 20 for 10 min
Batch: MSU-RK-5-100
Sample: MSU-RK-5-100
User: Admin
S/N: 000LC10009
Date: 10/22/2025 2:54 PM

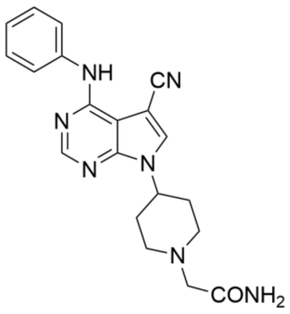

22

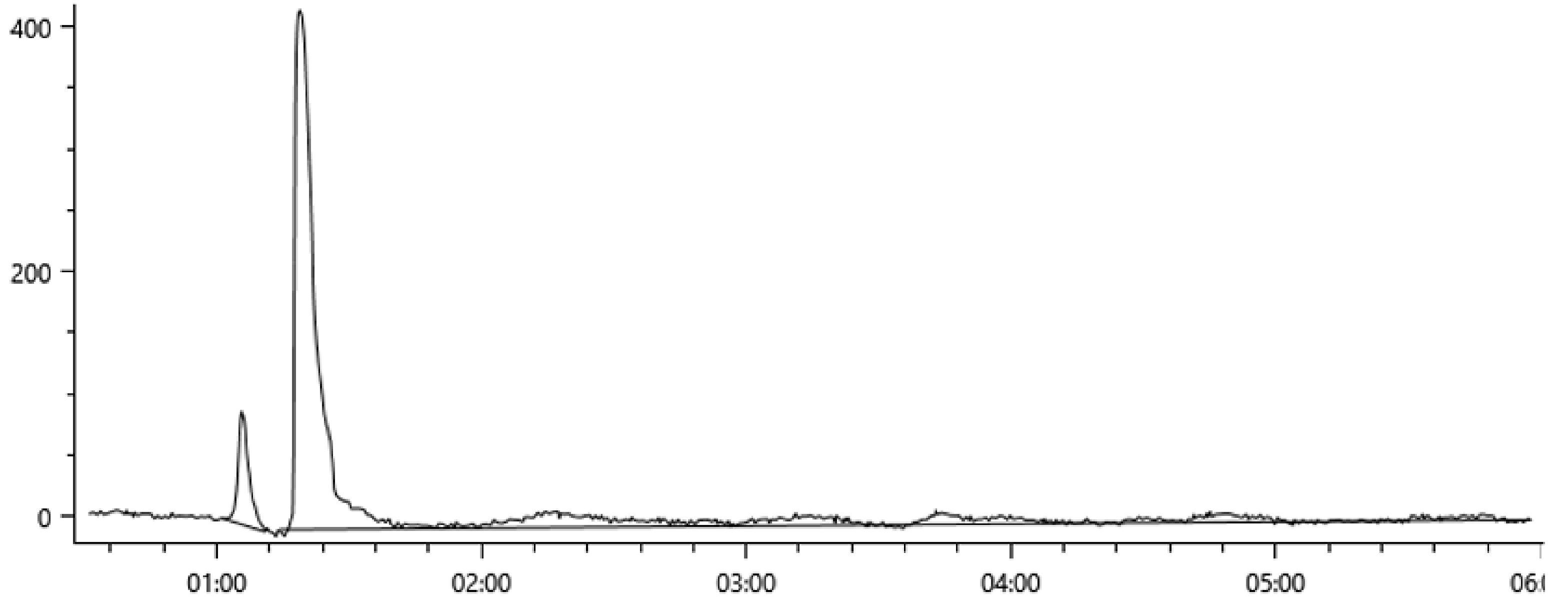

#	Compound	Retention Time	Area	% Area	Height	Conc.
1		01:06.0	270.8	14.5	103.49	0.000
2		01:19.4	3139.1	85.5	422.79	0.000
